# Supplementary material for: Structural dynamics and allosteric communication of a SARS-like bat coronavirus spike glycoprotein
Source: Biophys J. 2026 Apr 22;125(16):4420–36. doi: 10.1016/j.bpj.2026.04.023 (PMC13507448; doi:10.1016/j.bpj.2026.04.023)
Supplement: Supplementary file 1 [file mmc1.pdf]

Biophysical Journal, Volume *125*

Supplemental information

Structural dynamics and allosteric communication of a SARS-like bat coronavirus spike glycoprotein

Toheeb A. Balogun, Fiona L. Kearns, Carla Calvó-Tusell, Alexandra L. Tse, Cory M. Acreman, Lorenzo Casalino, Gorka Lasso, Emily Happy Miller, Kartik Chandran, Jason S. McLellan, and Rommie E. Amaro

Supplementary Information for

Structural Dynamics and Allosteric Communication of a SARS-Like Bat Coronavirus Spike Glycoprotein

Toheeb A. Balogun[1], Fiona L. Kearns[1,2], Carla Calvó-Tusell[1], Alexandra L. Tse[3], Cory M. Acreman[4,5], Lorenzo Casalino[1], Gorka Lasso[3,6], Emily Happy Miller[3,7], Kartik Chandran[3], Jason S. McLellan[4], Rommie E. Amaro[1#]

1. Department of Molecular Biology, University of California, San Diego, La Jolla, California, United States of America

2. Current Address: Institute of Physical Chemistry, University of Freiburg, Albertstrasse 21, 79104 Freiburg, Germany

3. Department of Microbiology & Immunology, Albert Einstein College of Medicine, Bronx, New York, New York, United States of America

4. Department of Molecular Biosciences, The University of Texas at Austin, Austin, Texas, United States of America

5. Current Address: Division of Biology and Biological Engineering, California Institute of Technology, Pasadena, California, United States of America

6. Current Address: Division of Infection and Immunity, University College London, London, UK.

7. Department of Medicine, Albert Einstein College of Medicine, Bronx, New York, New York, United States of America

[#]Corresponding author: ramaro@ucsd.edu

Contents

Figure S1: Modeling of missing regions of SHC014 GP
Figure S2: Root mean square deviation (RMSD) of SHC014 variant simulations
Figure S3: Trimeric area calculation for NTD, RBD, RBM, SD2 and FPPR
Figure S4: RBD trimeric area
Figure S5: RBD-core distances
Figure S6: Accessible surface area of SHC014 spike domains
Figure S7: F294/L294-K266 contacts
Figure S8: F294/L294-K288 contacts
Figure S9: F294/L294-D275 contacts
Figure S10: A835/D835-R830 contacts
Figure S11: R830-D555 contacts
Figure S12: A835/D835-D555 contacts
Figure S13: Salt-bridge formation and A835-R830-D555 area

Figure S14: R830-N270 glycan contacts
Figure S15: R830-N590 glycan
Figure S16: Conformational flexibility of the 620-loop in SHC014 WT and mutants
Figure S17: Domain-domain communication
Figure S18: Allosteric communication pathways in SHC014 variants with non-primary pathways

Table S1: Complete details of SHC014 computational models and simulation information
Table S2: Summary of the full-length S protein all-atom MD simulations
Table S3. Glycan compositions for chains A, B, and C.
Table S4: Summary of average trimeric domain areas (Å²) for NTD, RBM, SD2, and FPPR in SHC014 WT and mutants.
Table S5: Statistical analysis of FPPR trimeric area differences across SHC014 spike variants

Movie S1: Contact among A835, D555, and R830.
Movie S2: Contact among D835, D555, and R830.
Movie S3: Contact among F294, K266, K288, and D275.
Movie S4: Contact among L294, K266, K288, and D275.
Movie S5: Dynamics of the DM 620-loop relative to the WT spike.

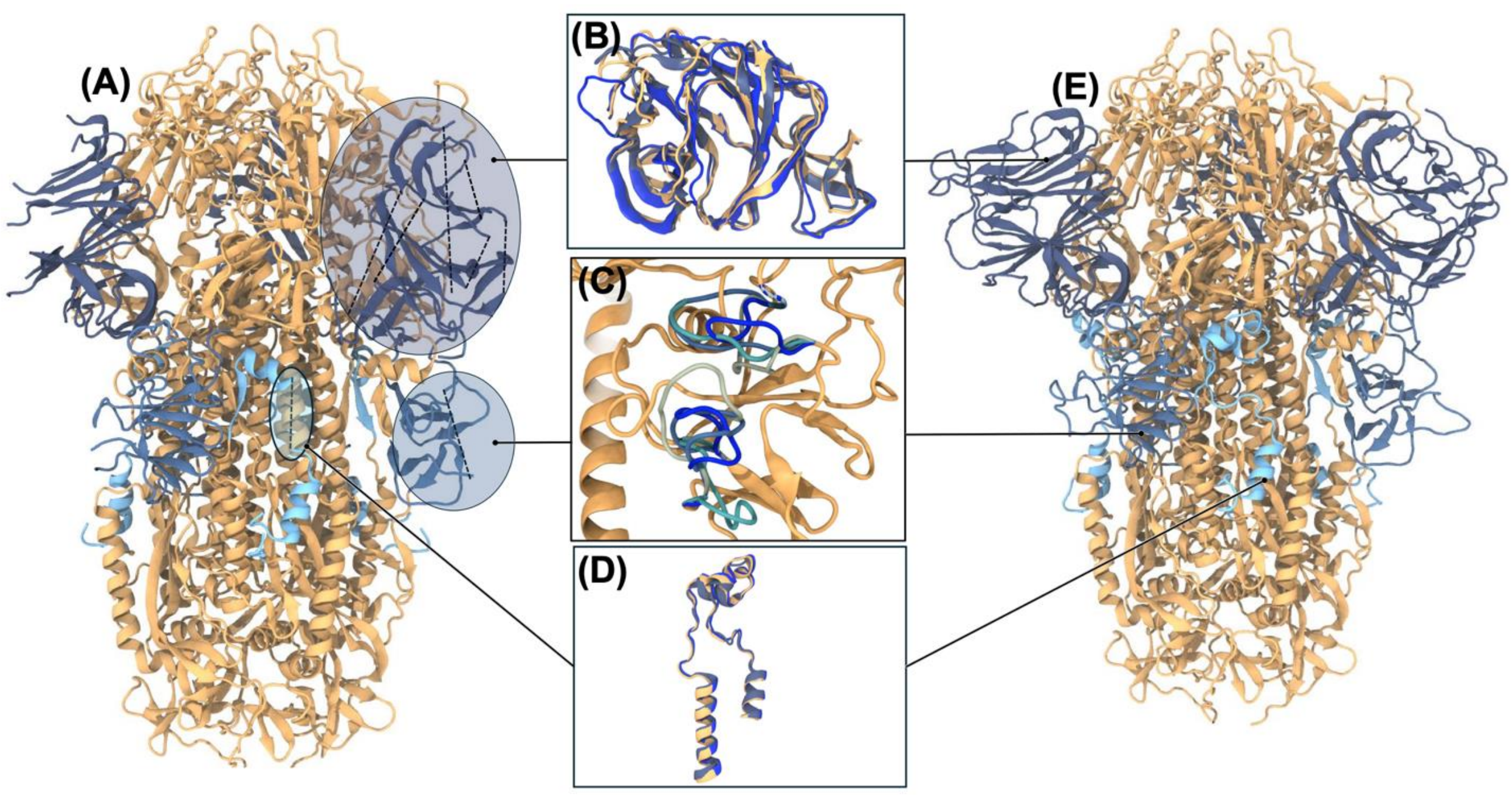

Figure S1: Modeling of missing regions of SHC014 GP (A) Wildtype cryoEM structure of SCH01with missing regions highlighted in broken lines with light filled sphere (B) NTD modeling using multiple protein structure prediction tools such as Alphafold (darkblue), Prime (blue) and SwissModel (Gold) (C) Modeling of missing loops in SD2 domain using ChimeraX (D) FPPR modeling using PDB 7JJI as template shown in blue, SwissModel (Gold) and Alphafold (darkblue) (E) SHC014 GP ectodomain with missing regions filled.

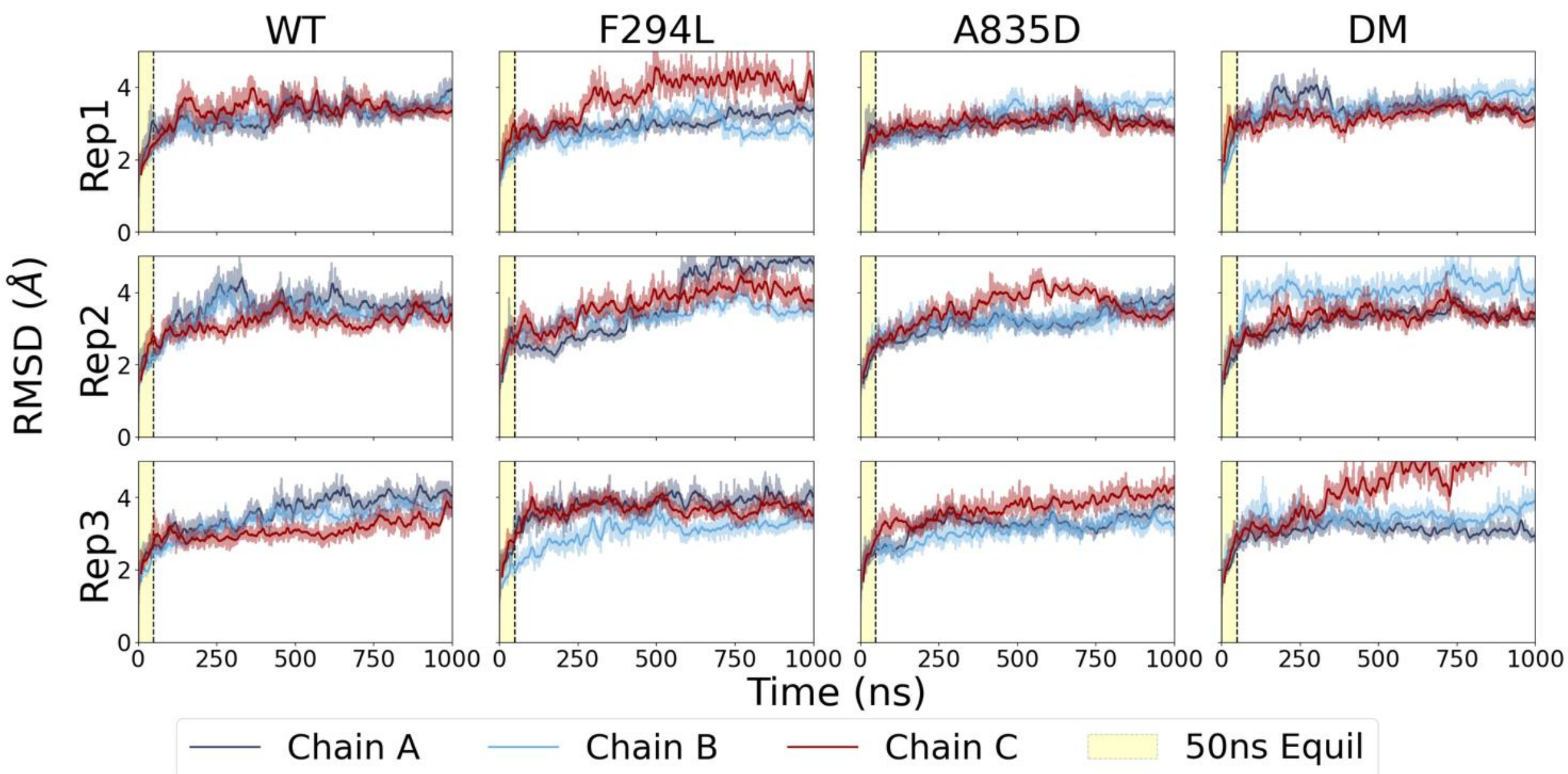

Figure S2: Root mean square deviation (RMSD) of SHC014 variants, the 50ns equilibration step before production run is shown in yellow.

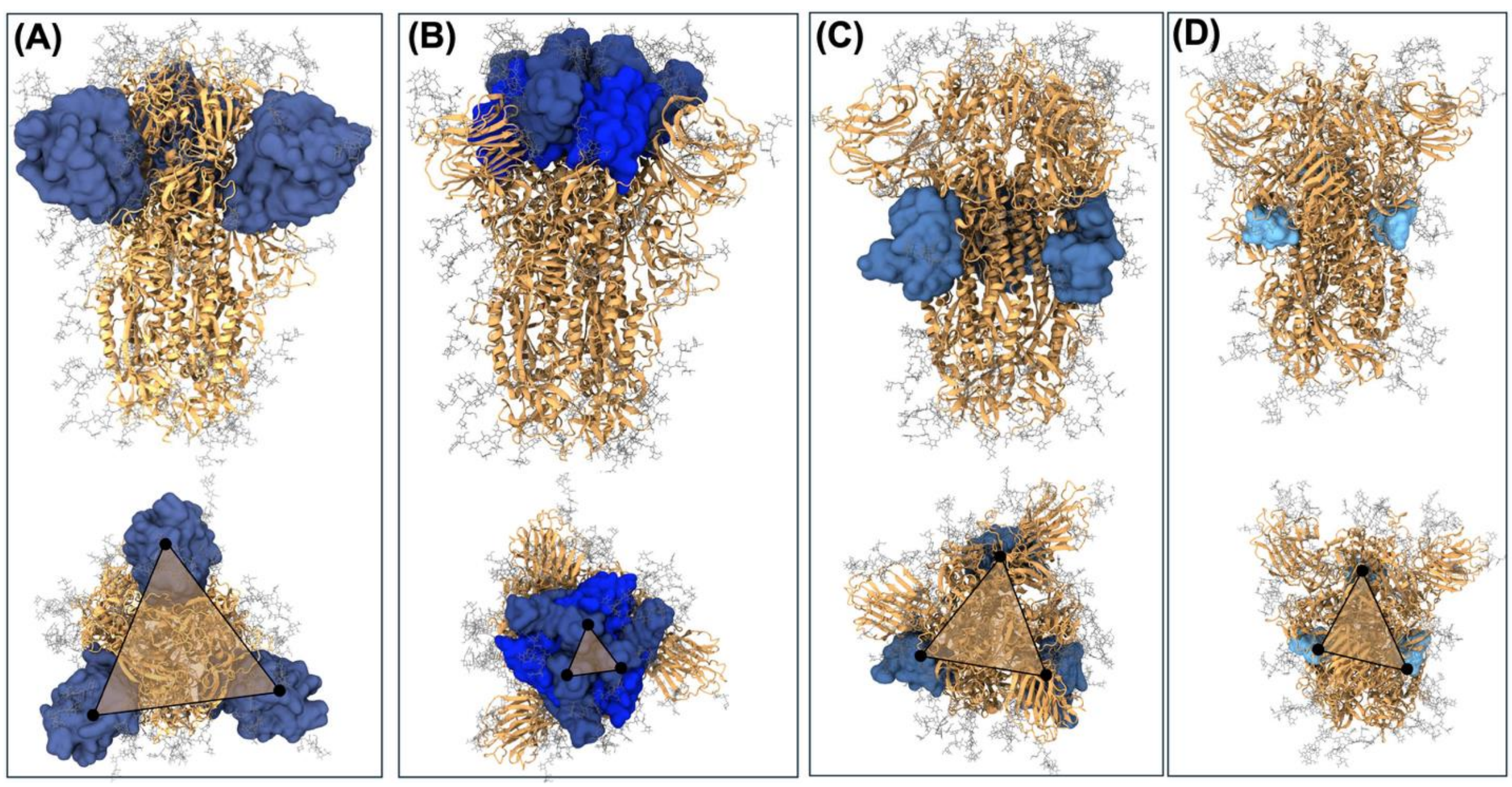

Figure S3: Trimeric area calculations of (A) NTD; N-terminal domain (B) RBM; Receptor binding domain (C) SD2; Sub-domain 2 (D) FPPR; Fusion Peptide Proximal Region

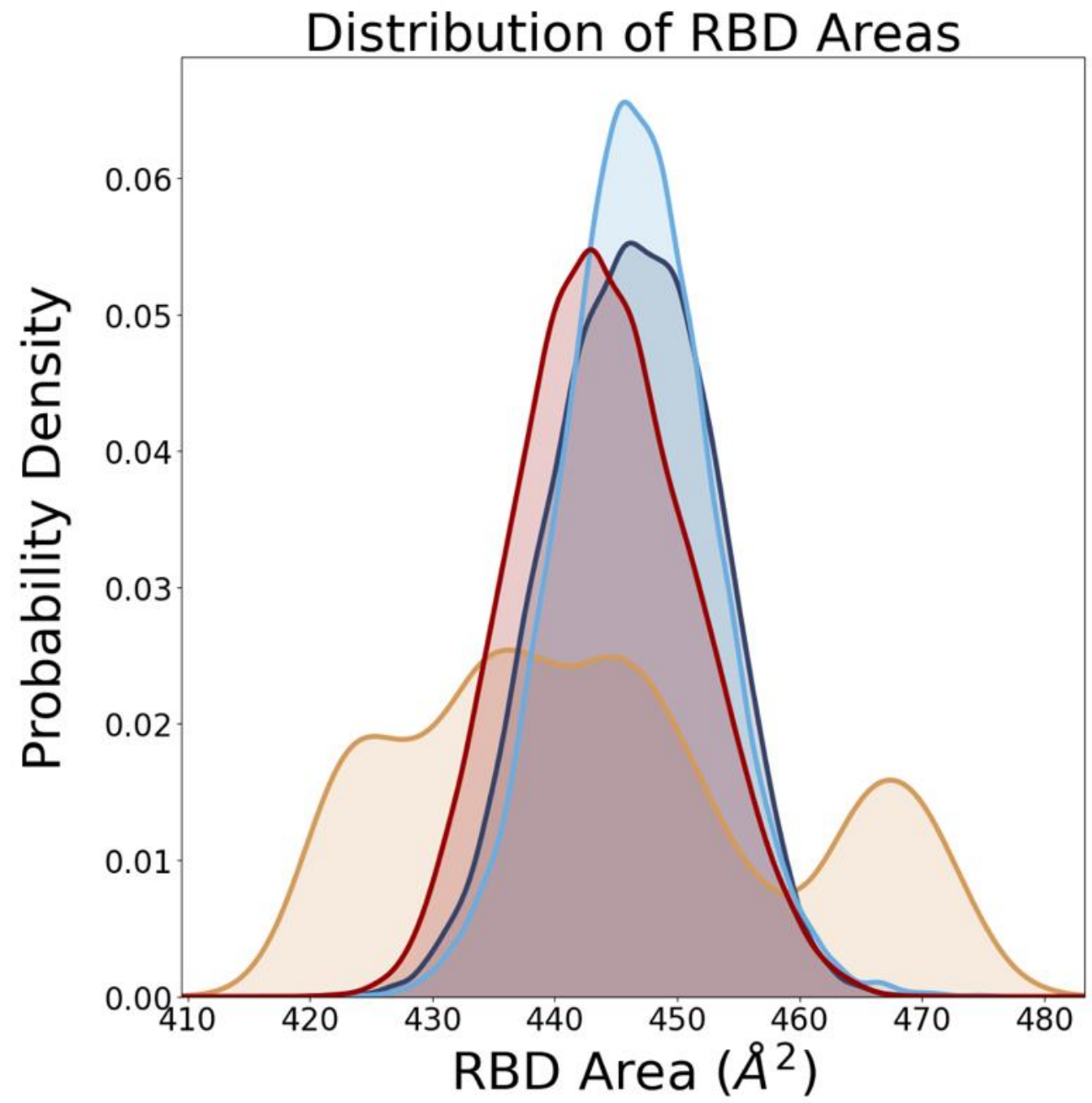

Figure S4: RBD trimeric area over the course of the simulations. WT is shown in gold, F294L in dark blue, A835D in cyan, and DM in red.

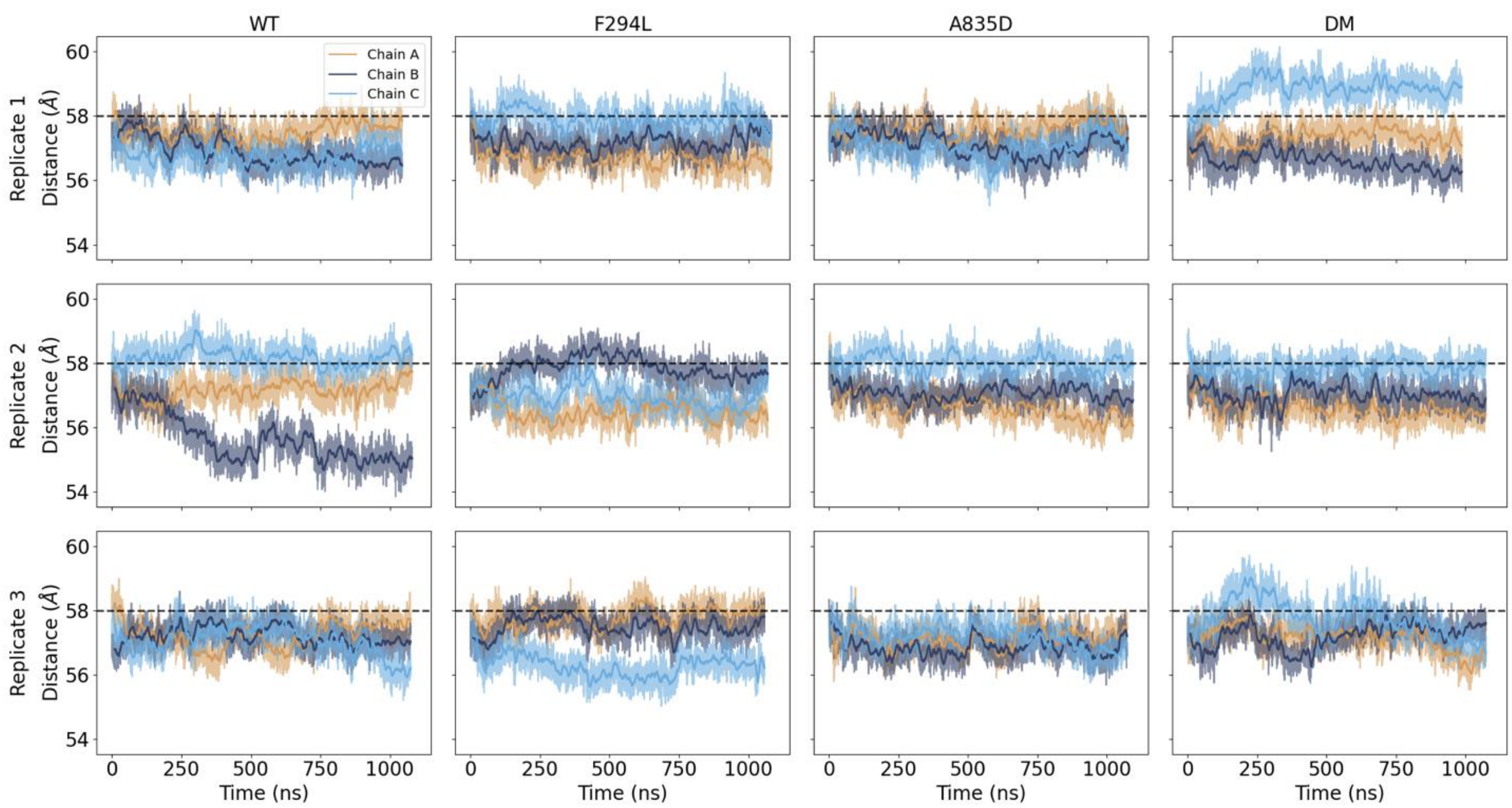

Figure S5: Time series analysis of RBD-core distances per replica across the variants depicting the propensity of RBD opening in each chain. Chain A is shown in gold, chain B in dark blue and chain C in cyan.

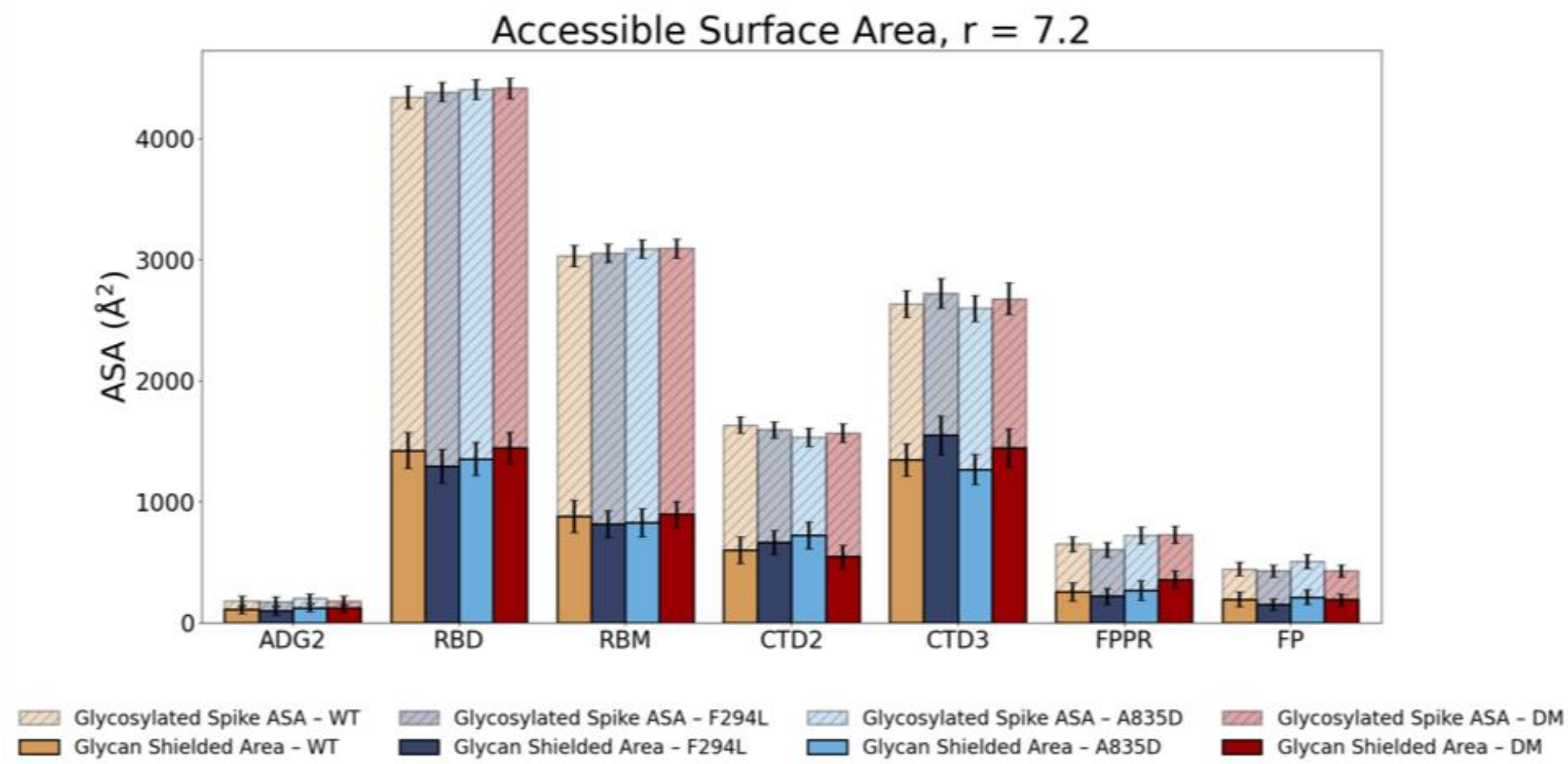

Figure S6. Accessible surface area of SHC014 spike domains with a probe radius of 7.2Å. ADG2 referred to the ADG2 binding site.

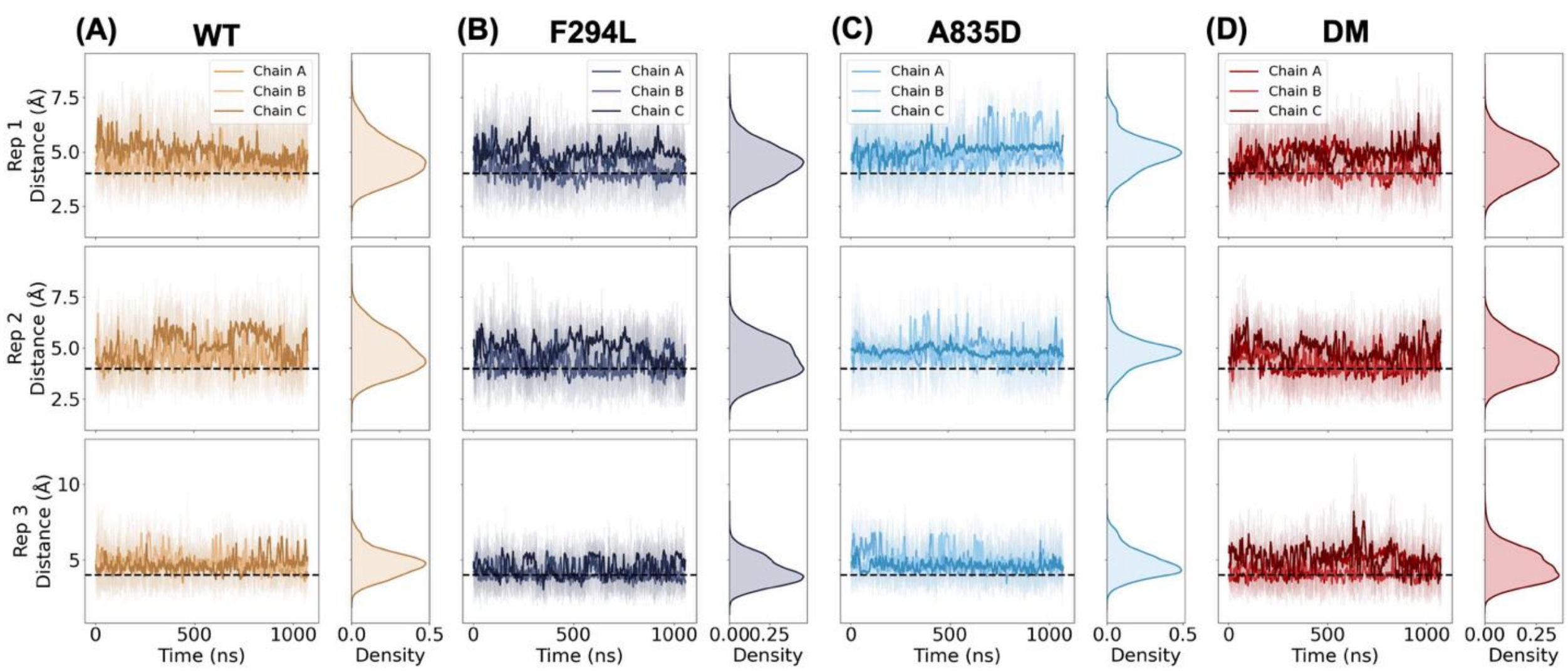

Figure S7: F294/L294-K266 contact analysis across the variants with a distance threshold of 4Å shown as dashed lines.

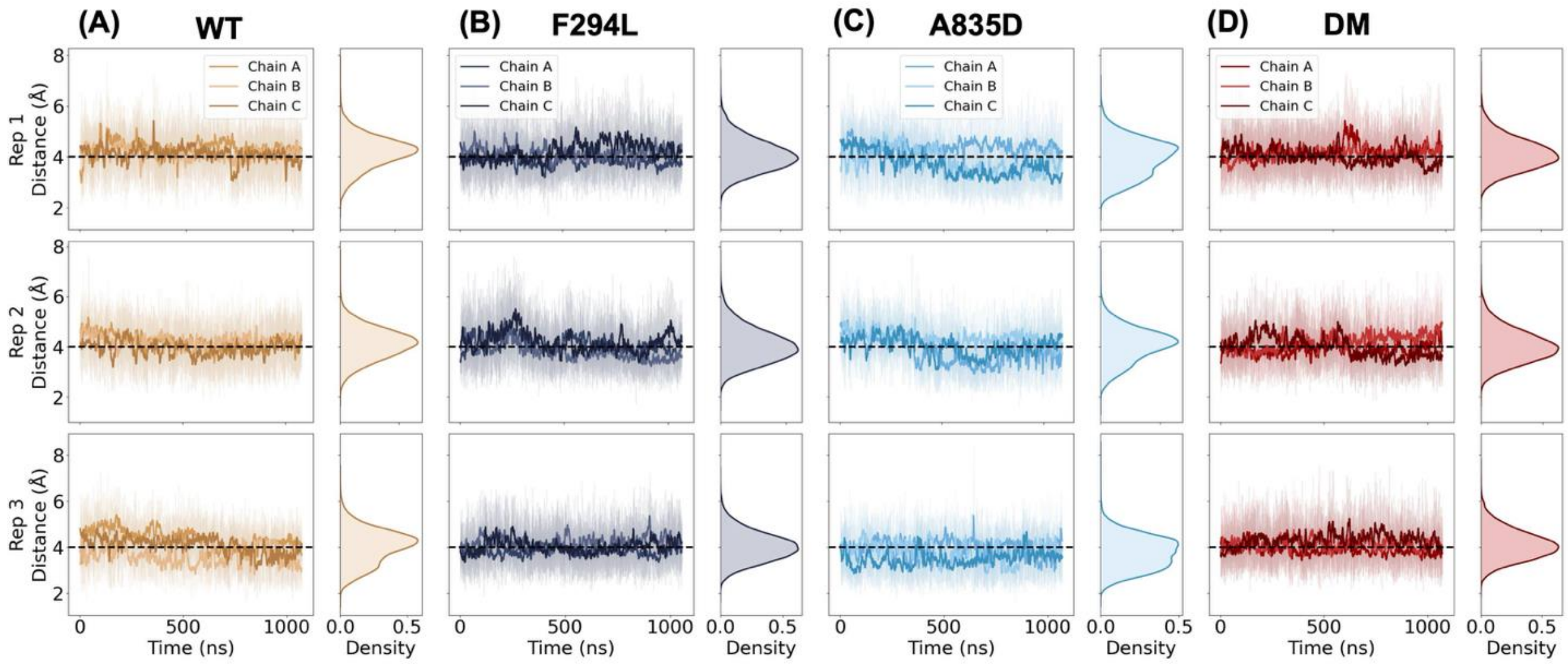

Figure S8: F294/L294-K288 contact analysis across the variants with a distance threshold of 4Å shown as dashed lines

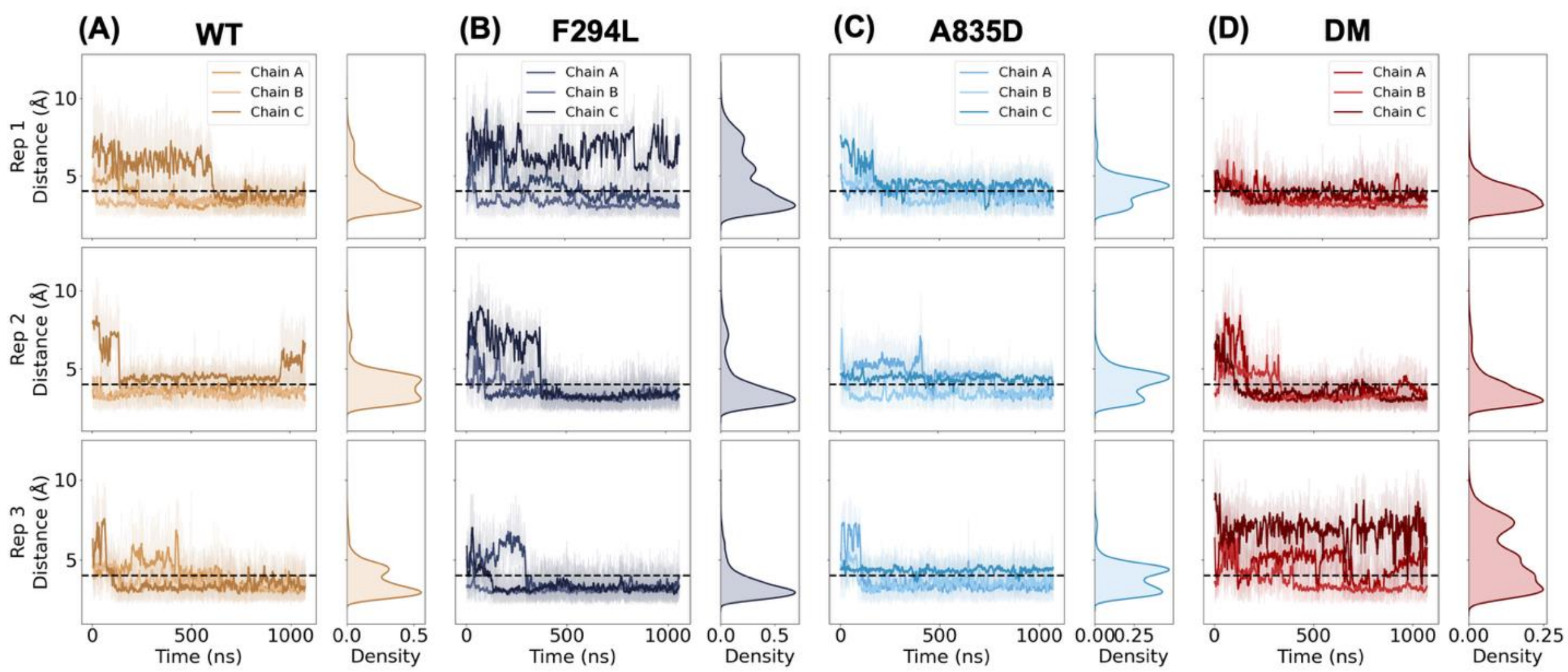

Figure S9: F294/L294-D275 contact analysis across the variants with a distance threshold of 4Å shown as dashed lines.

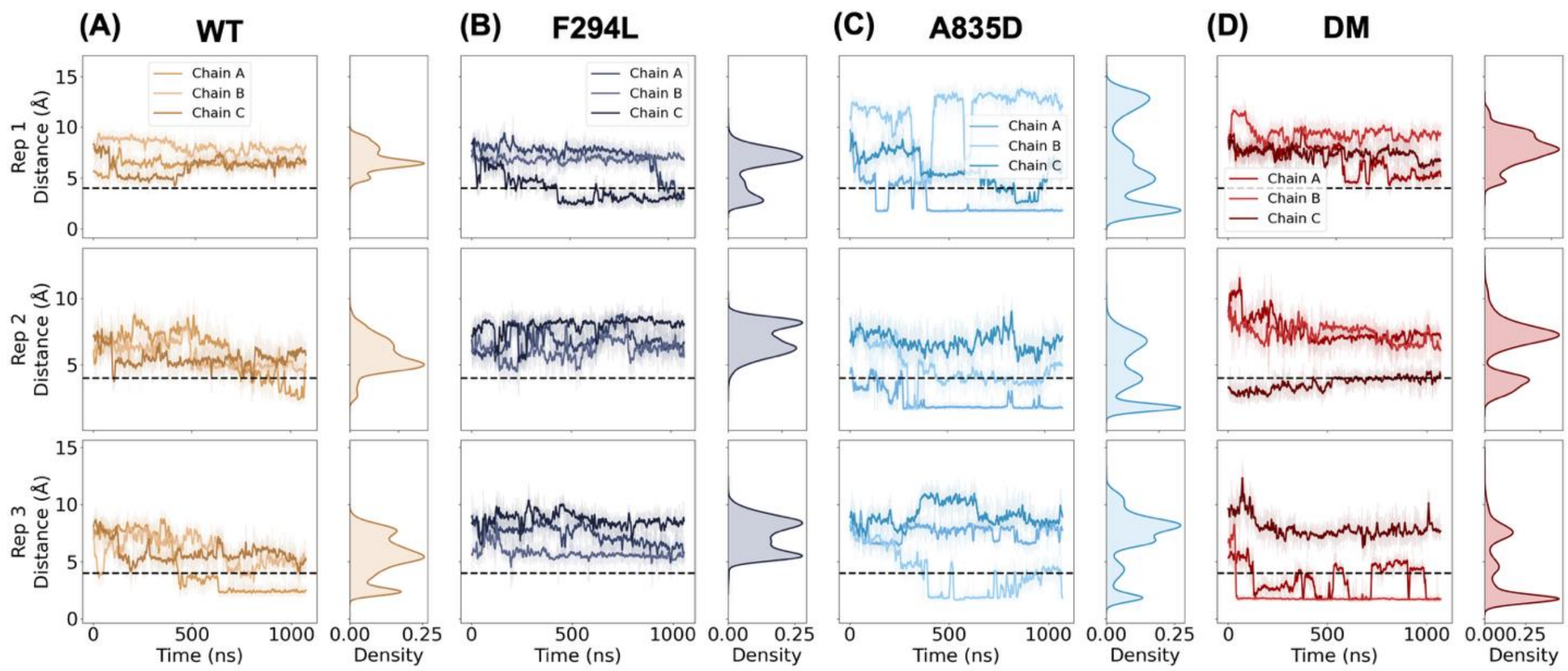

Figure S10: A835/D835-R830 contact analysis across the variants with a distance threshold of 4Å shown as dashed lines.

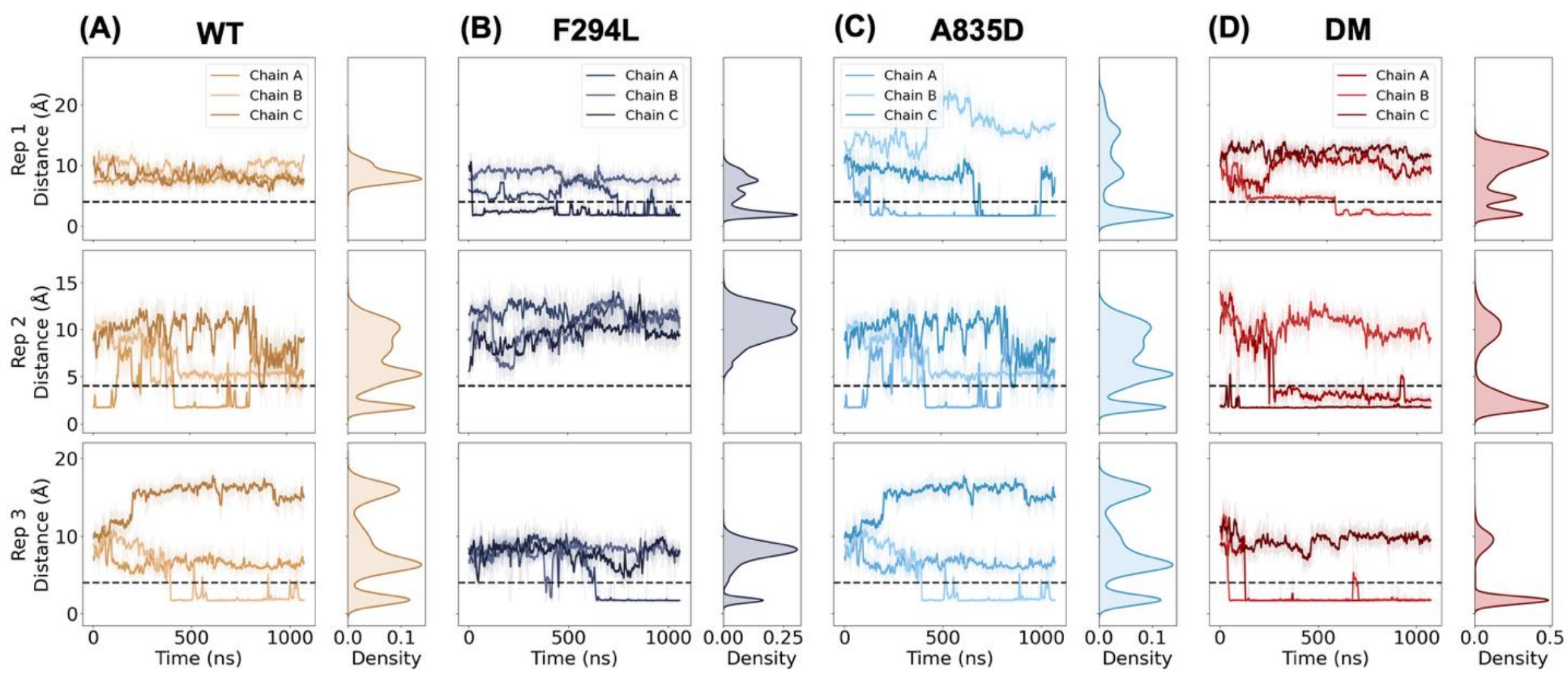

Figure S11: R830-D555 contact analysis across the variants with a distance threshold of 4Å shown as dashed lines.

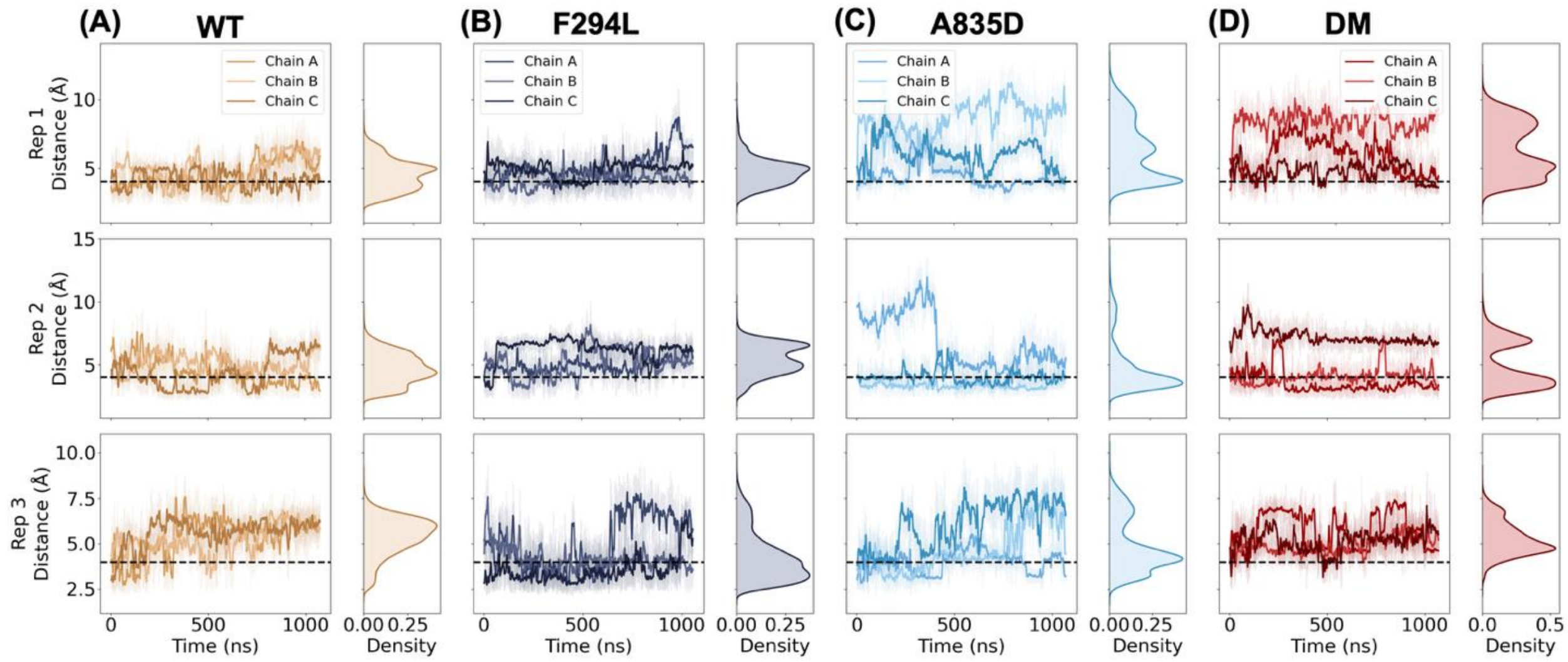

Figure S12: A835/D835-D555 contact analysis across the variants with a distance threshold of 4Å shown as dashed lines.

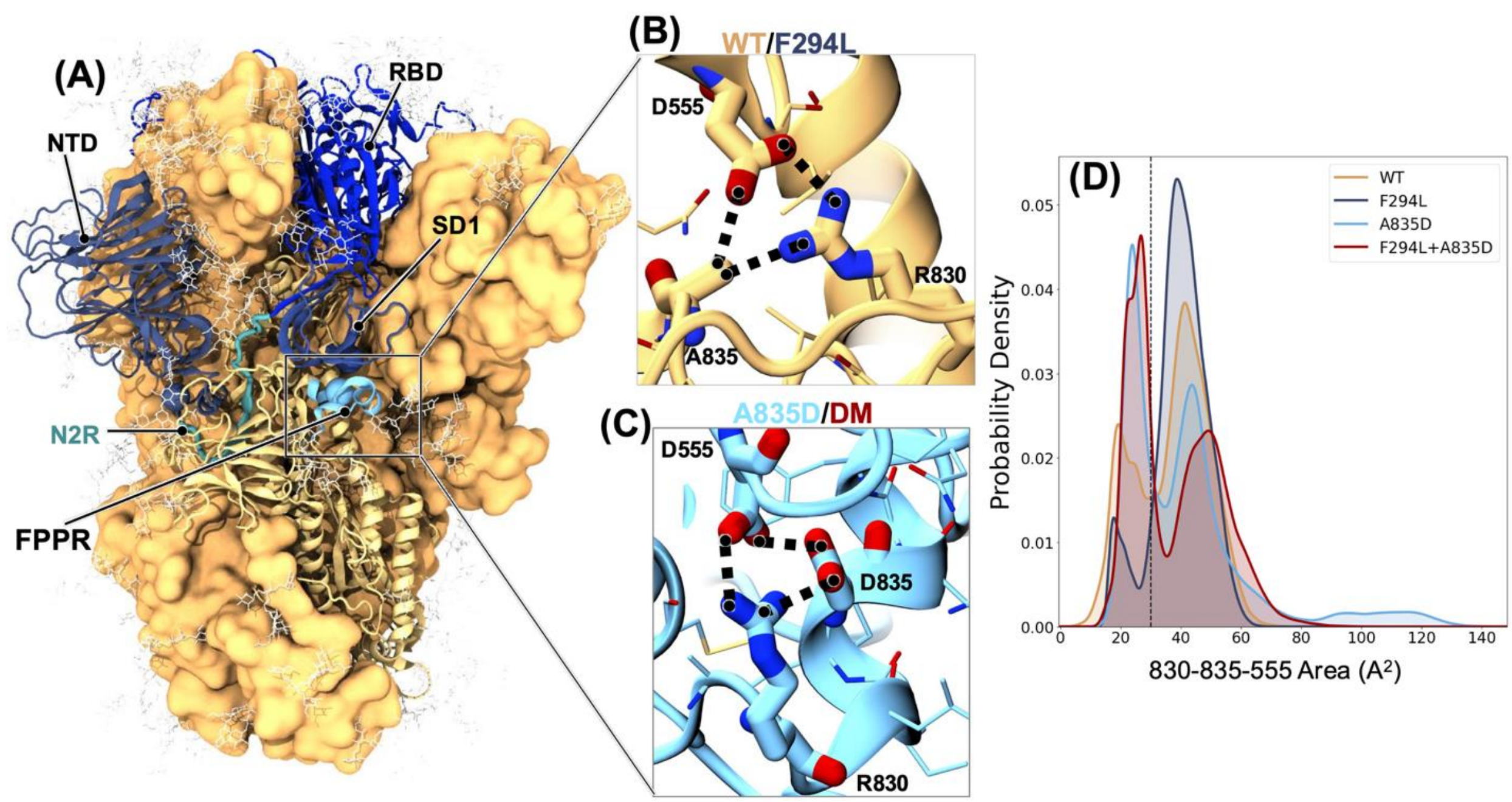

Figure S13. Salt bridge formation (A) Highlight of different domains in the SHC014 spike ectodomain, which includes the NTD: N-terminal domain; RBD: Receptor binding domain; SD1: Subdomain-1; N2R Linker and FPPR: Fusion peptide proximal region. Position 835 is shown as a cyan sphere matching the A835D color (B). Close view of A835-R830-D555 area (C) Close view of D835-R830-D555 area (D). Formation and persistence of the salt-bridge triad between R830-D555-X835 with a tight salt-bridge having a threshold of <30Å^2 shown as a broken line. X = A835 or D835.

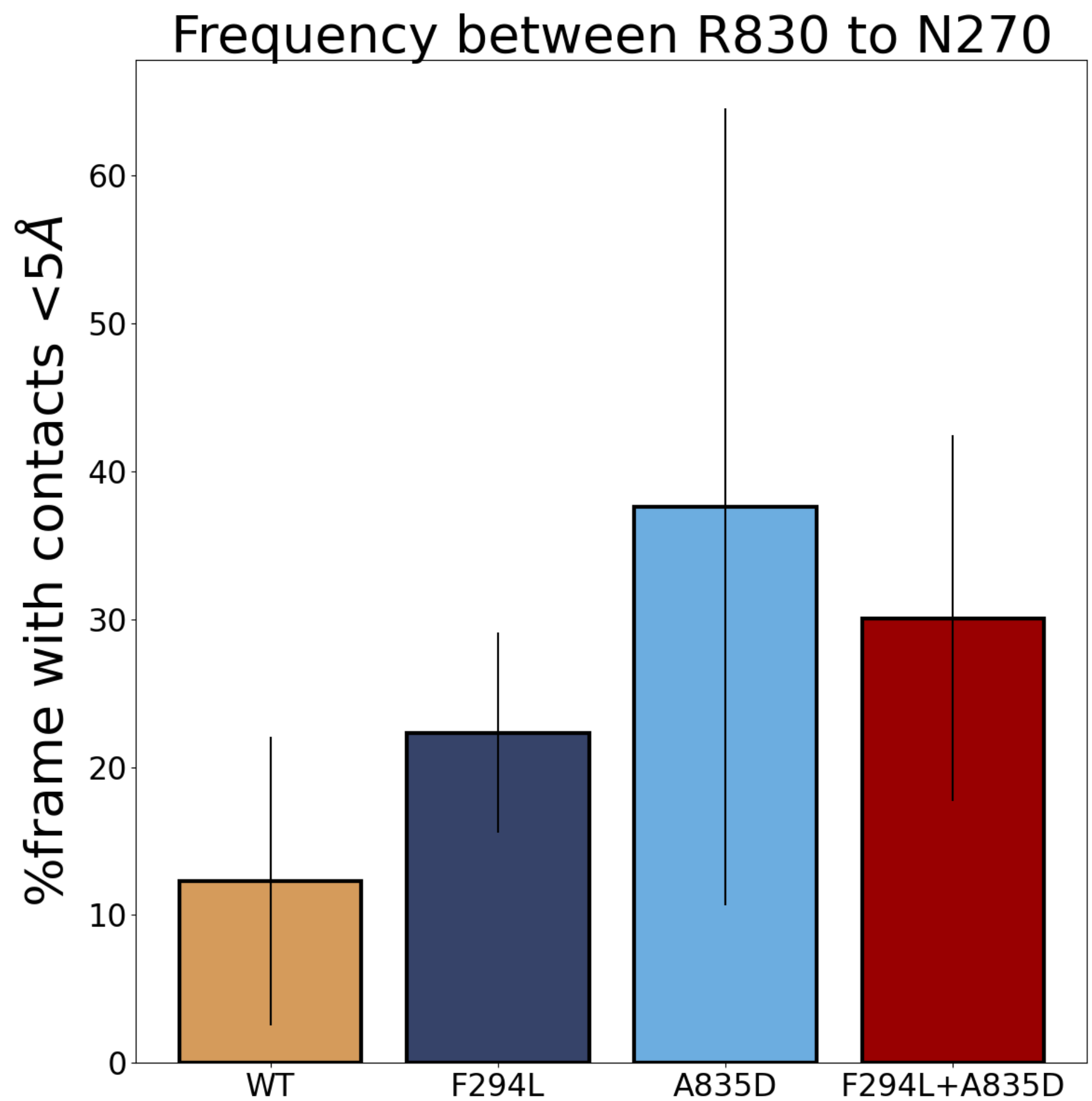

Figure S14. Frequency of contact across variants between R830 and glycan N270.

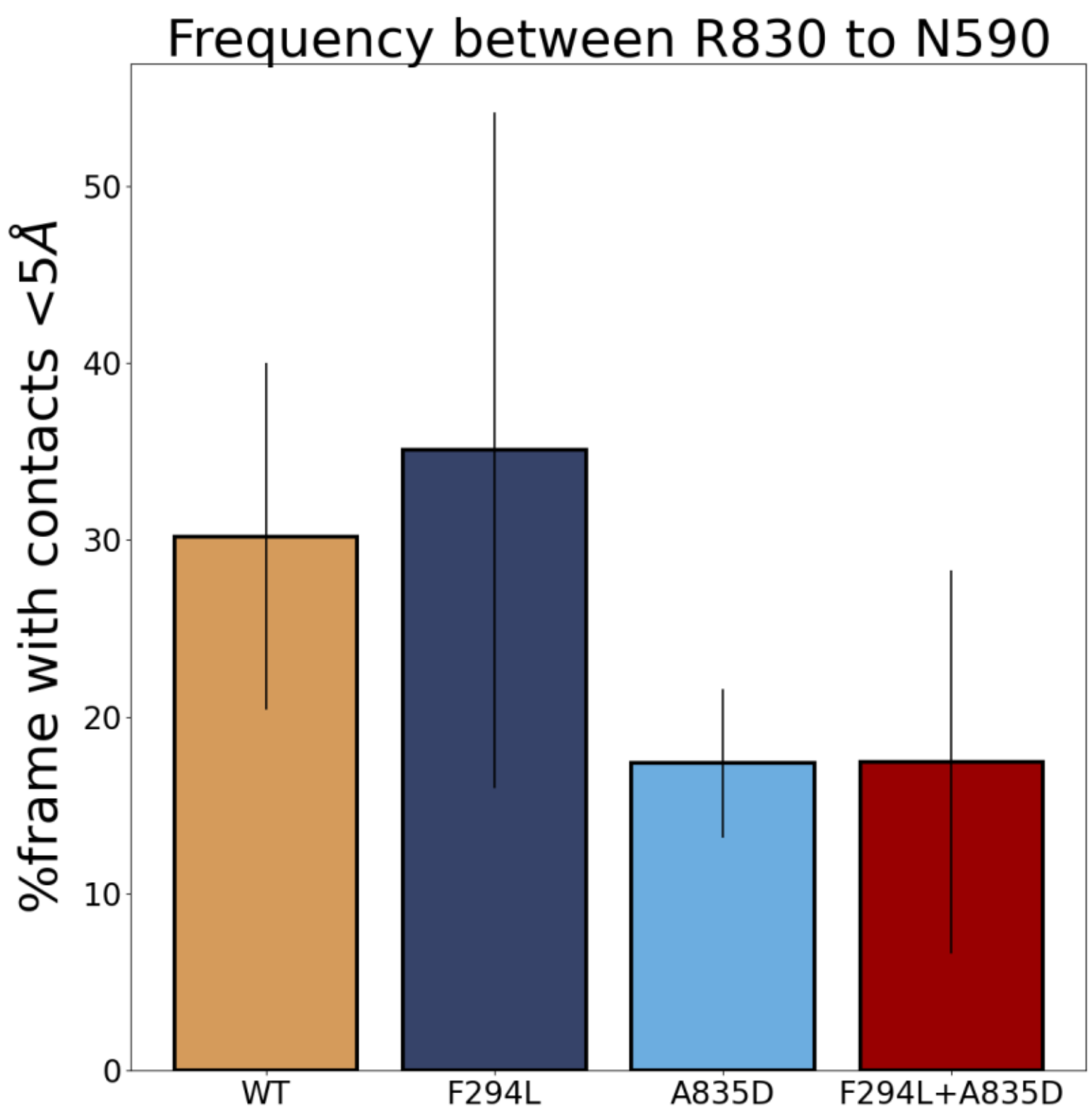

Figure S15. Frequency of contact across variants between R830 and glycan N590.

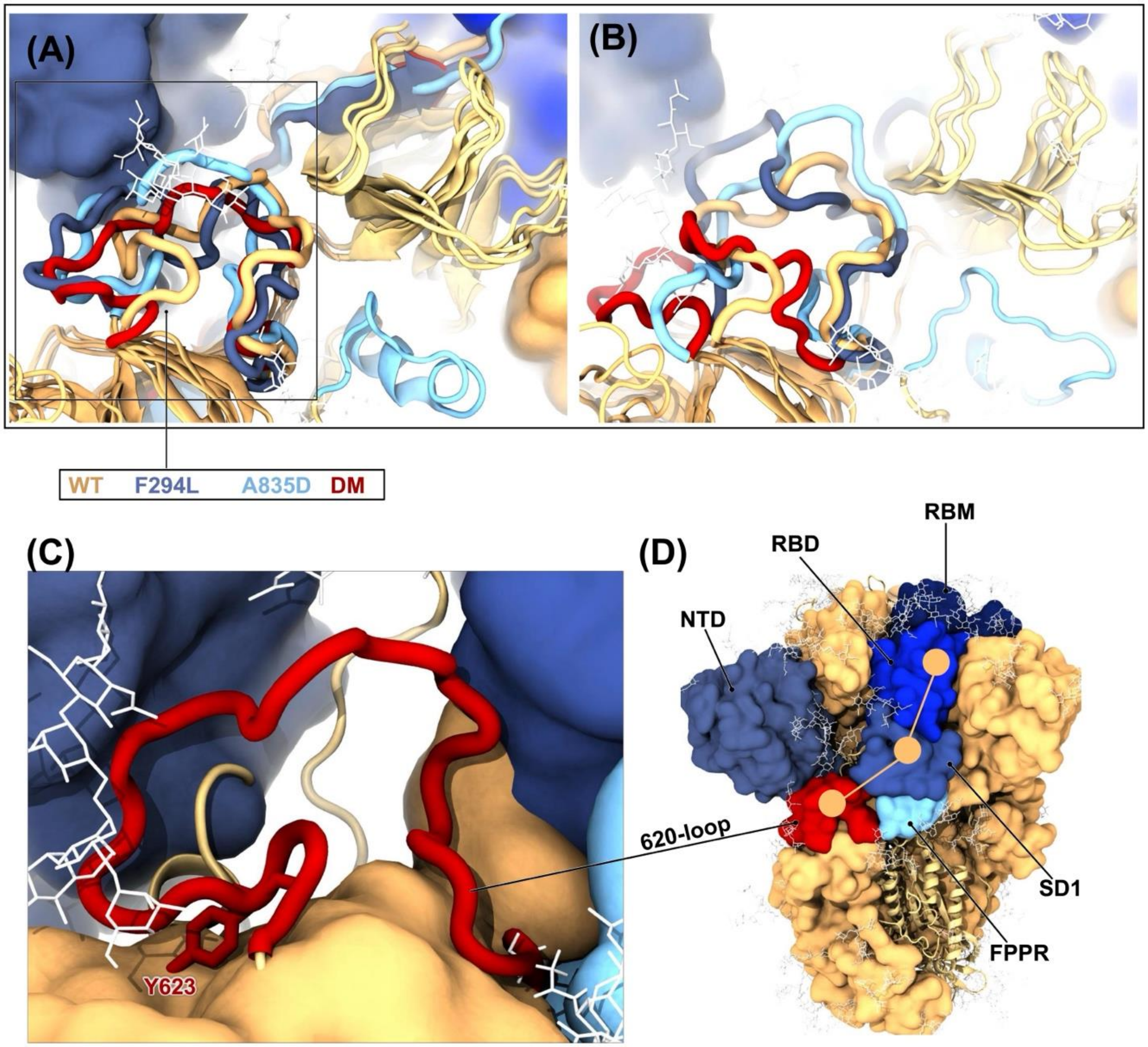

Figure S16. Conformational flexibility of the 620-loop across the variants (A). Similar conformation of the 620-loop across the variants (B). Varying degrees in the conformational dynamics of the 620-loop with the double mutant (shown in red), 620-loop showing the highest degree of flexibility (C). A close view of Y623 within the 620loop (D). A proposed model of allosteric communication is depicted by connecting circles from the 620loop through sub-domain-1 to the RBD.

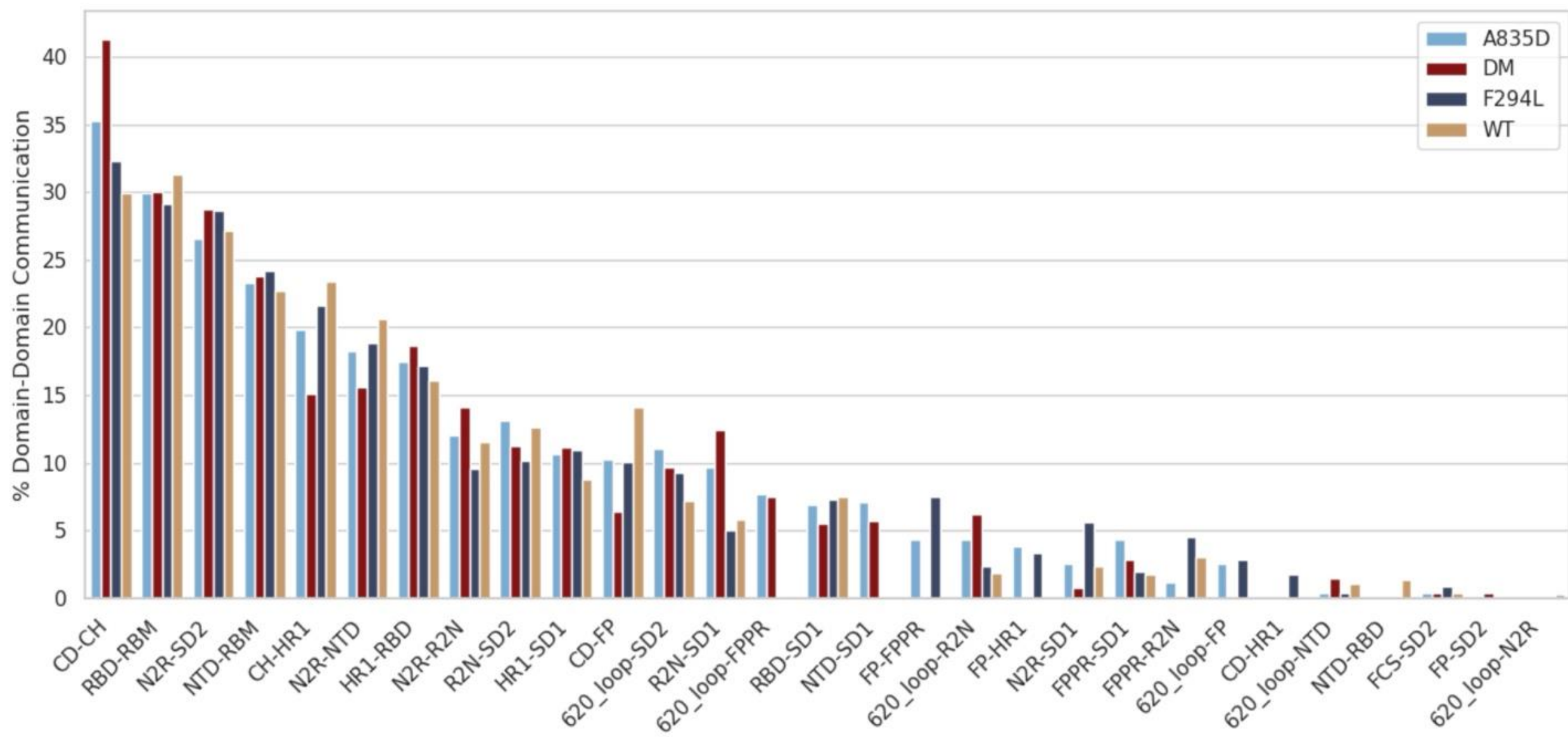

Figure S17. Domain-domain communication across the SHC014 spike variants.

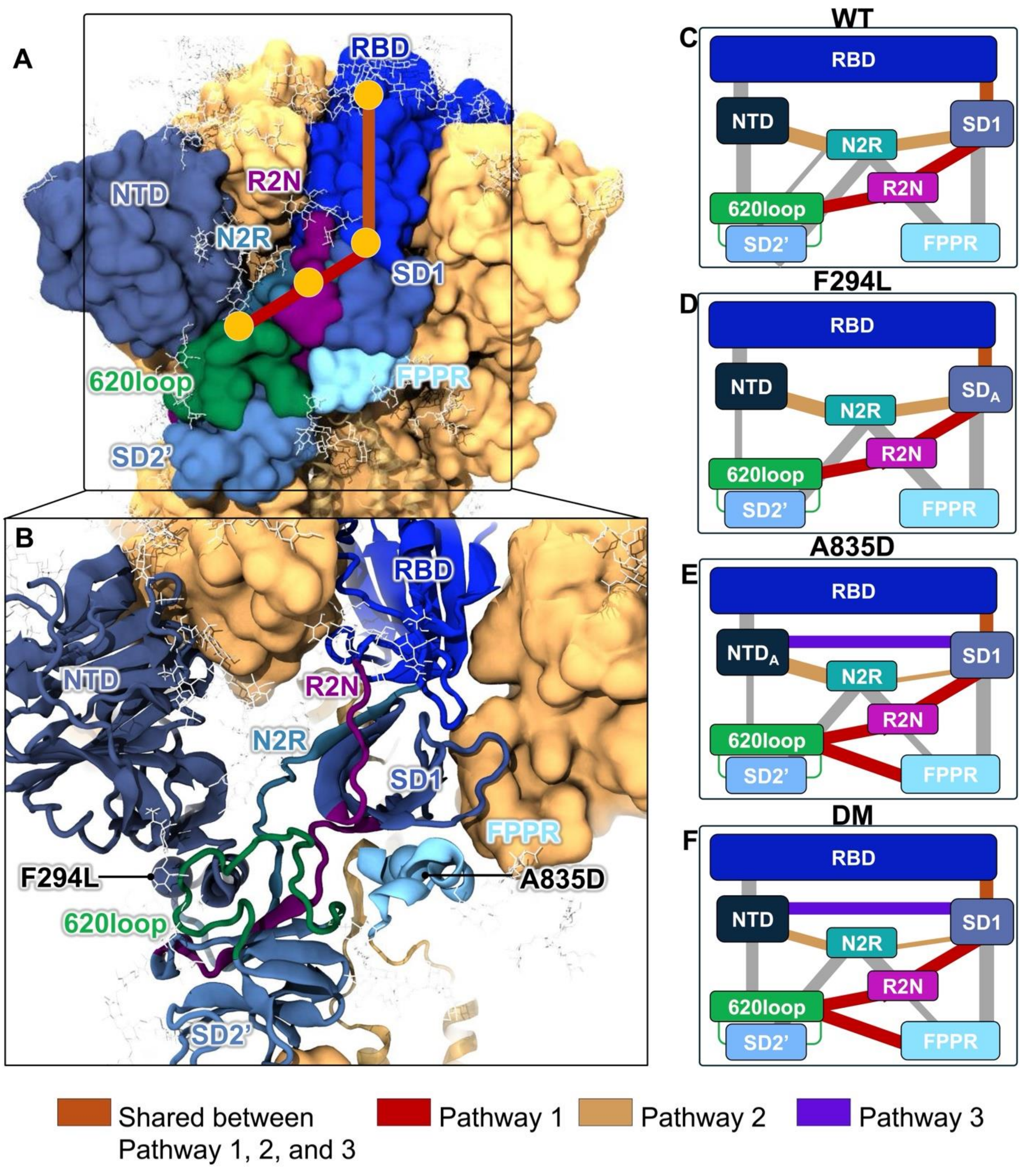

Figure S18. Allosteric communication pathways in SHC014 variants. Non-primary pathways are shown in gray in C-F.

Supplementary Tables

Table S1: Complete details of SHC014 computational models and simulation information:

	**WT**	**F294L**	**A835D**	**DM**
Total #atoms	972591	972585	972600	972588
#protein atoms/residues	50928 / 308316	50925 / 308315	50934 / 308319	50931 / 308316
#glycan atoms/glycans	11415 / 57	11415 / 57	11415 / 57	11415 / 57
#water atoms/residues	908598 / 302866	908595 / 302865	908598 / 302866	908589 / 302863
#Na/Cl ions	843 / 807	843 / 807	846 / 807	846 / 807
Disulfides per monomer/ Total disulfide bonds	15 / 45	15 / 45	15 / 45	15/45
System Dimensions (ÅxÅxÅ)	215.00 X 214.99 X 214.98	215.00 X 214.99 X 214.98	215.00 X 214.99 X 214.98	215.00 X 214.99 X 214.98

Table S2: Summary of the full-length S protein all-atom MD simulations.

**Variant**	**Rep1 (ns)**	**Rep2 (ns)**	**Rep3 (ns)**
WT	1042.3	1078.3	1074.2
F294L	1080.3	1068.8	1056.8
A835D	1073.8	1093.2	1072
DM	1075.6	1072	1073.1

Table S3. Glycan compositions for chains A, B, and C.

CHAIN A

#	SITE	TYPE	STRUCTURE	SEQUENCE
G1	N66	M5	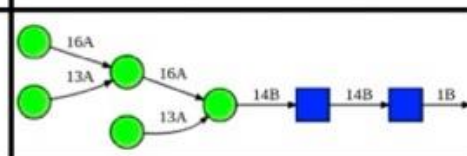	aDMan(1→6)[aDMan(1→3)]aDMan(1→6)<br>[aDMan(1→3)]bDMan(1→4)bDGlcNAc(1→4)bDGlcNAc(1→)PROA-66
G2	N110	FA2G2S1	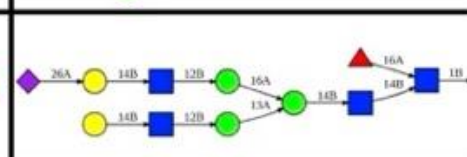	aDNeu5Ac(2→6)bDGal(1→4)bDGlcNAc(1→2)aDMan(1→6)<br>[bDGal(1→4)bDGlcNAc(1→2)aDMan(1→3)]bDMan(1→4)bDGlcNAc(1→4)<br>[aLFuc(1→6)]bDGlcNAc(1→)PROA-110
G3	N120	M5	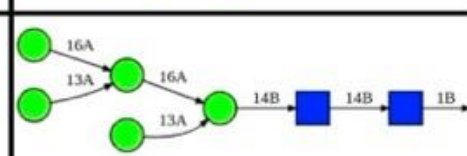	aDMan(1→6)[aDMan(1→3)]aDMan(1→6)<br>[aDMan(1→3)]bDMan(1→4)bDGlcNAc(1→4)bDGlcNAc(1→)PROA-120
G4	N145	FA2G2S1	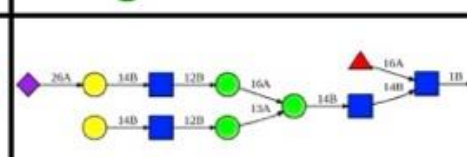	aDNeu5Ac(2→6)bDGal(1→4)bDGlcNAc(1→2)aDMan(1→6)<br>[bDGal(1→4)bDGlcNAc(1→2)aDMan(1→3)]bDMan(1→4)bDGlcNAc(1→4)<br>[aLFuc(1→6)]bDGlcNAc(1→)PROA-145
G5	N159	FA2G2S2	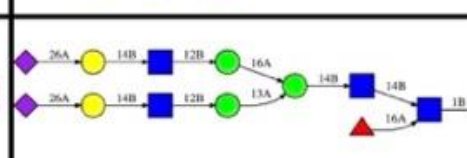	aDNeu5Ac(2→6)bDGal(1→4)bDGlcNAc(1→2)aDMan(1→6)<br>[aDNeu5Ac(2→6)bDGal(1→4)bDGlcNAc(1→2)aDMan(1→3)]bDMan(1→4)bDGlcNAc(1→4)<br>[aLFuc(1→6)]bDGlcNAc(1→)PROA-159
G6	N228	M8	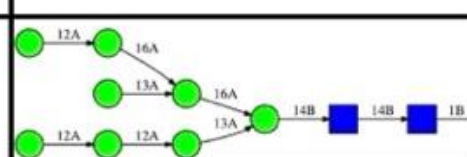	aDMan(1→2)aDMan(1→6)[aDMan(1→3)]aDMan(1→6)<br>[aDMan(1→2)aDMan(1→2)aDMan(1→3)]bDMan(1→4)bDGlcNAc(1→4)bDGlcNAc(1→)PROA-228
G7	N270	FA3	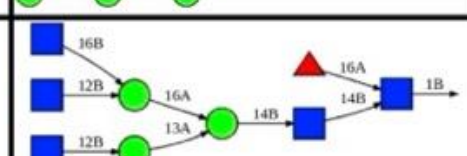	bDGlcNAc(1→6)[bDGlcNAc(1→2)]aDMan(1→6)<br>[bDGlcNAc(1→2)aDMan(1→3)]bDMan(1→4)bDGlcNAc(1→4)[aLFuc(1→6)]bDGlcNAc(1→)PROA-270
G8	N319	FA2	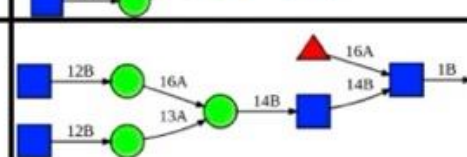	bDGlcNAc(1→2)aDMan(1→6)[bDGlcNAc(1→2)aDMan(1→3)]bDMan(1→4)bDGlcNAc(1→4)<br>[aLFuc(1→6)]bDGlcNAc(1→)PROA-319
G9	N331	FA2	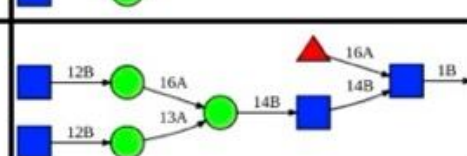	bDGlcNAc(1→2)aDMan(1→6)[bDGlcNAc(1→2)aDMan(1→3)]bDMan(1→4)bDGlcNAc(1→4)<br>[aLFuc(1→6)]bDGlcNAc(1→)PROA-331
G10	N358	FA2G2S1	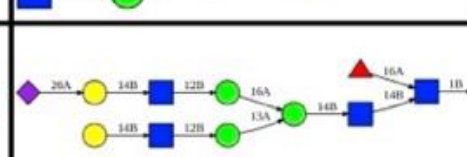	aDNeu5Ac(2→6)bDGal(1→4)bDGlcNAc(1→2)aDMan(1→6)<br>[bDGal(1→4)bDGlcNAc(1→2)aDMan(1→3)]bDMan(1→4)bDGlcNAc(1→4)<br>[aLFuc(1→6)]bDGlcNAc(1→)PROA-358
G11	N590	FA2	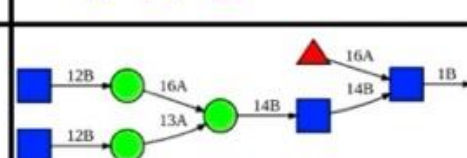	bDGlcNAc(1→2)aDMan(1→6)[bDGlcNAc(1→2)aDMan(1→3)]bDMan(1→4)bDGlcNAc(1→4)<br>[aLFuc(1→6)]bDGlcNAc(1→)PROA-590
G12	N603	A2	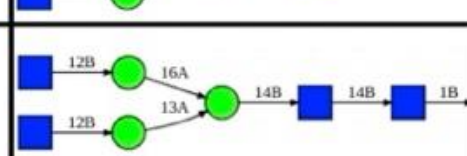	bDGlcNAc(1→2)aDMan(1→6)<br>[bDGlcNAc(1→2)aDMan(1→3)]bDMan(1→4)bDGlcNAc(1→4)bDGlcNAc(1→)PROA-603
G13	N692	M6	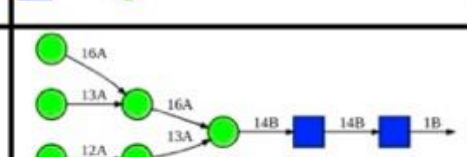	aDMan(1→6)[aDMan(1→3)]aDMan(1→6)<br>[aDMan(1→2)aDMan(1→3)]bDMan(1→4)bDGlcNAc(1→4)bDGlcNAc(1→)PROA-692
G14	N700	Hybrid G1	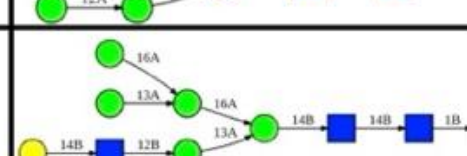	bDGal(1→4)bDGlcNAc(1→2)aDMan(1→3)[aDMan(1→6)<br>[aDMan(1→3)]aDMan(1→6)]bDMan(1→4)bDGlcNAc(1→4)bDGlcNAc(1→)PROA-700
G15	N784	M6	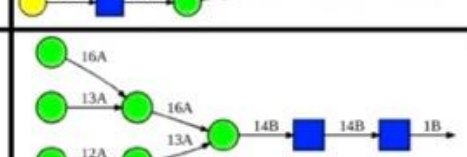	aDMan(1→6)[aDMan(1→3)]aDMan(1→6)<br>[aDMan(1→2)aDMan(1→3)]bDMan(1→4)bDGlcNAc(1→4)bDGlcNAc(1→)PROA-784
G16	N1057	FA2G2S1	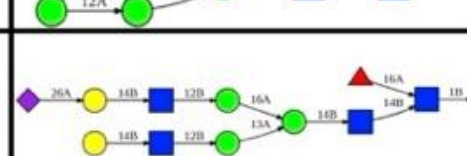	aDNeu5Ac(2→6)bDGal(1→4)bDGlcNAc(1→2)aDMan(1→6)<br>[bDGal(1→4)bDGlcNAc(1→2)aDMan(1→3)]bDMan(1→4)bDGlcNAc(1→4)<br>[aLFuc(1→6)]bDGlcNAc(1→)PROA-1057
G17	N1081	FA2	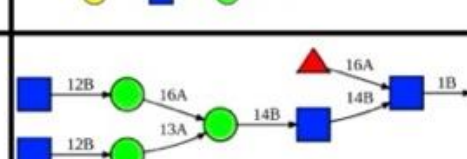	bDGlcNAc(1→2)aDMan(1→6)[bDGlcNAc(1→2)aDMan(1→3)]bDMan(1→4)bDGlcNAc(1→4)<br>[aLFuc(1→6)]bDGlcNAc(1→)PROA-1081
G18	N1117	FA1	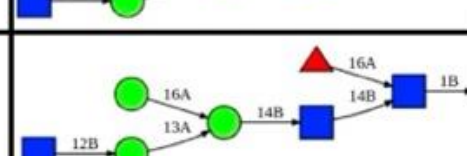	bDGlcNAc(1→2)aDMan(1→3)[aDMan(1→6)]bDMan(1→4)bDGlcNAc(1→4)<br>[aLFuc(1→6)]bDGlcNAc(1→)PROB-1117
G19	S311	O-glycan	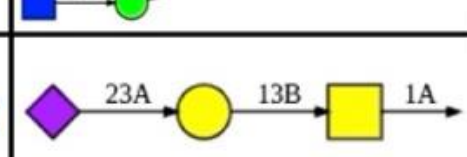	aDNeu5Ac(2→3)bDGal(1→3)aDGalNAc(1→)PROA-311

CHAIN B

#	SITE	TYPE	STRUCTURE	SEQUENCE
G20	N66	M5	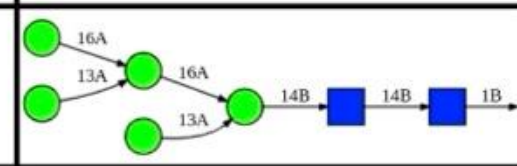	aDMan(1→6)[aDMan(1→3)]aDMan(1→6) [aDMan(1→3)]bDMan(1→4)bDGlcNAc(1→4)bDGlcNAc(1→)PROB-66
G21	N110	FA2G2S2	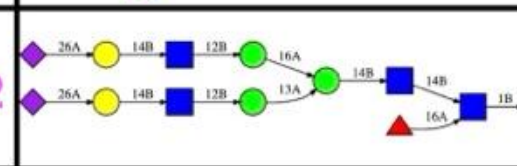	aDNeu5Ac(2→6)bDGal(1→4)bDGlcNAc(1→2)aDMan(1→6) [aDNeu5Ac(2→6)bDGal(1→4)bDGlcNAc(1→2)aDMan(1→3)]bDMan(1→4)bDGlcNAc(1→4) [aLFuc(1→6)]bDGlcNAc(1→)PROB-110
G22	N120	FA2	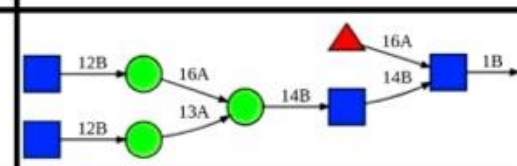	bDGlcNAc(1→2)aDMan(1→6)[bDGlcNAc(1→2)aDMan(1→3)]bDMan(1→4)bDGlcNAc(1→4) [aLFuc(1→6)]bDGlcNAc(1→)PROB-120
G23	N145	FA3	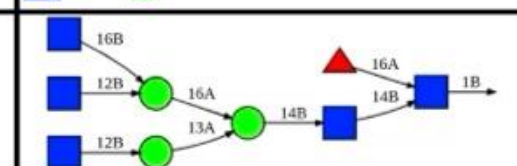	bDGlcNAc(1→6)[bDGlcNAc(1→2)]aDMan(1→6) [bDGlcNAc(1→2)aDMan(1→3)]bDMan(1→4)bDGlcNAc(1→4)[aLFuc(1→6)]bDGlcNAc(1→)PROB-145
G24	N159	M5	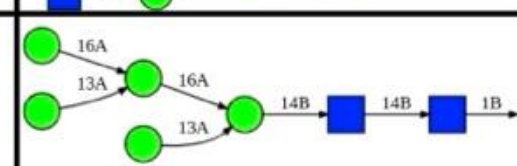	aDMan(1→6)[aDMan(1→3)]aDMan(1→6) [aDMan(1→3)]bDMan(1→4)bDGlcNAc(1→4)bDGlcNAc(1→)PROB-159
G25	N228	M9	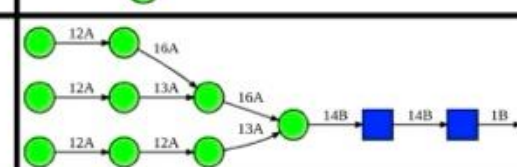	aDMan(1→2)aDMan(1→6)[aDMan(1→2)aDMan(1→3)]aDMan(1→6) [aDMan(1→2)aDMan(1→2)aDMan(1→3)]bDMan(1→4)bDGlcNAc(1→4)bDGlcNAc(1→)PROB-228
G26	N270	FA3G3S1	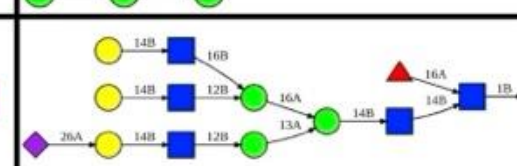	aDNeu5Ac(2→6)bDGal(1→4)bDGlcNAc(1→2)aDMan(1→3)[bDGal(1→4)bDGlcNAc(1→6) [bDGal(1→4)bDGlcNAc(1→2)]aDMan(1→6)]bDMan(1→4)bDGlcNAc(1→4) [aLFuc(1→6)]bDGlcNAc(1→)PROB-270
G27	N319	FA2	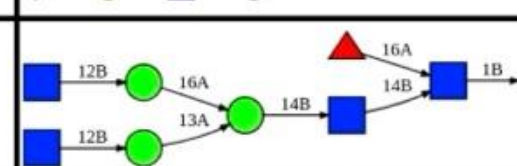	bDGlcNAc(1→2)aDMan(1→6)[bDGlcNAc(1→2)aDMan(1→3)]bDMan(1→4)bDGlcNAc(1→4) [aLFuc(1→6)]bDGlcNAc(1→)PROB-319
G28	N331	FA1	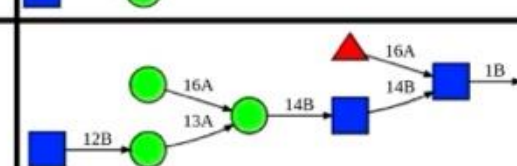	bDGlcNAc(1→2)aDMan(1→3)[aDMan(1→6)]bDMan(1→4)bDGlcNAc(1→4) [aLFuc(1→6)]bDGlcNAc(1→)PROB-331
G29	N358	FA2G2S2	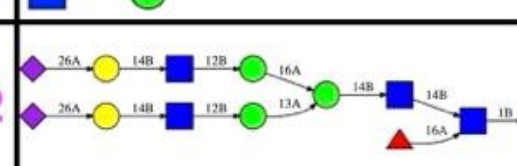	aDNeu5Ac(2→6)bDGal(1→4)bDGlcNAc(1→2)aDMan(1→6) [aDNeu5Ac(2→6)bDGal(1→4)bDGlcNAc(1→2)aDMan(1→3)]bDMan(1→4)bDGlcNAc(1→4) [aLFuc(1→6)]bDGlcNAc(1→)PROB-358
G30	N590	M5	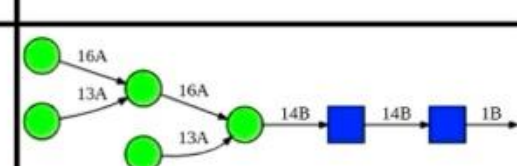	aDMan(1→6)[aDMan(1→3)]aDMan(1→6) [aDMan(1→3)]bDMan(1→4)bDGlcNAc(1→4)bDGlcNAc(1→)PROB-590
G31	N603	FA2	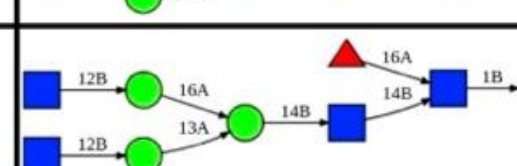	bDGlcNAc(1→2)aDMan(1→6)[bDGlcNAc(1→2)aDMan(1→3)]bDMan(1→4)bDGlcNAc(1→4) [aLFuc(1→6)]bDGlcNAc(1→)PROB-603
G32	N692	M5	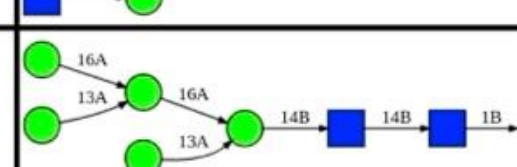	aDMan(1→6)[aDMan(1→3)]aDMan(1→6) [aDMan(1→3)]bDMan(1→4)bDGlcNAc(1→4)bDGlcNAc(1→)PROB-692
G33	N700	M5	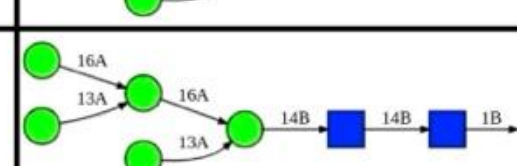	aDMan(1→6)[aDMan(1→3)]aDMan(1→6) [aDMan(1→3)]bDMan(1→4)bDGlcNAc(1→4)bDGlcNAc(1→)PROB-700
G34	N784	M7	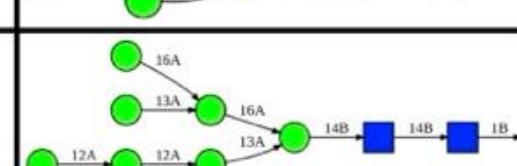	aDMan(1→2)aDMan(1→2)aDMan(1→3)[aDMan(1→6) [aDMan(1→3)]aDMan(1→6)]bDMan(1→4)bDGlcNAc(1→4)bDGlcNAc(1→)PROB-784
G35	N1057	M5	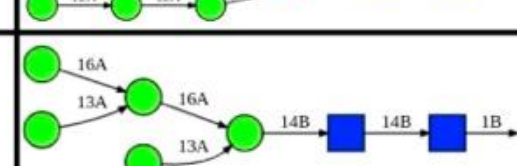	aDMan(1→6)[aDMan(1→3)]aDMan(1→6) [aDMan(1→3)]bDMan(1→4)bDGlcNAc(1→4)bDGlcNAc(1→)PROB-1057
G36	N1081	A2	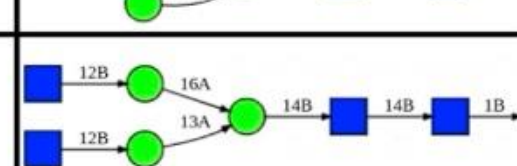	bDGlcNAc(1→2)aDMan(1→6) [bDGlcNAc(1→2)aDMan(1→3)]bDMan(1→4)bDGlcNAc(1→4)bDGlcNAc(1→)PROB-1081
G37	N1117	FA3	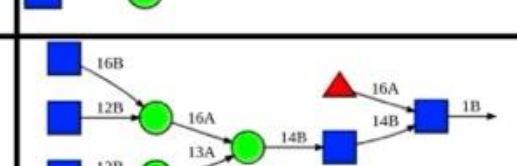	bDGlcNAc(1→6)[bDGlcNAc(1→2)]aDMan(1→6) [bDGlcNAc(1→2)aDMan(1→3)]bDMan(1→4)bDGlcNAc(1→4)[aLFuc(1→6)]bDGlcNAc(1→)PROB-1117
G38	S311	O-glycan	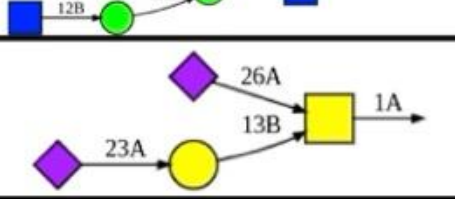	aDNeu5Ac(2→3)bDGal(1→3)[aDNeu5Ac(2→6)]aDGalNAc(1→)PROB-311

CHAIN C

#	SITE	TYPE	STRUCTURE	SEQUENCE
G39	N66	M5	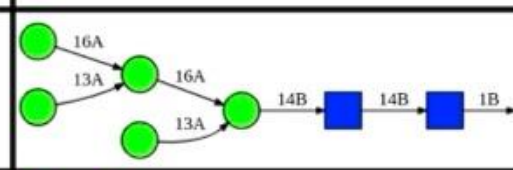	aDMan(1→6)[aDMan(1→3)]aDMan(1→6)[aDMan(1→3)]bDMan(1→4)bDGlcNAc(1→4)bDGlcNAc(1→)PROC-66
G40	N110	M8	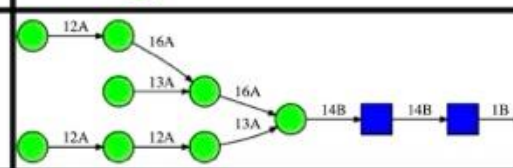	aDMan(1→2)aDMan(1→6)[aDMan(1→3)]aDMan(1→6)[aDMan(1→2)aDMan(1→2)aDMan(1→3)]bDMan(1→4)bDGlcNAc(1→4)bDGlcNAc(1→)PROC-110
G41	N120	M5	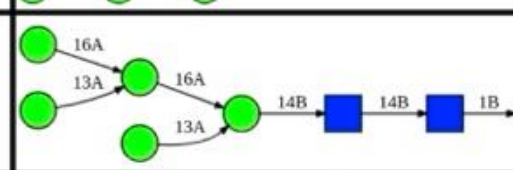	aDMan(1→6)[aDMan(1→3)]aDMan(1→6)[aDMan(1→3)]bDMan(1→4)bDGlcNAc(1→4)bDGlcNAc(1→)PROC-120
G42	N145	FA2	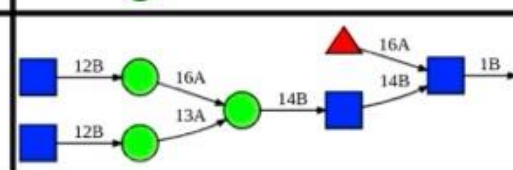	bDGlcNAc(1→2)aDMan(1→6)[bDGlcNAc(1→2)aDMan(1→3)]bDMan(1→4)bDGlcNAc(1→4)[aLFuc(1→6)]bDGlcNAc(1→)PROC-145
G43	N159	FA2G2S1	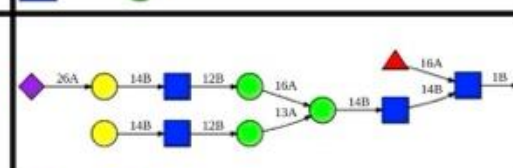	aDNeu5Ac(2→6)bDGal(1→4)bDGlcNAc(1→2)aDMan(1→6)[bDGal(1→4)bDGlcNAc(1→2)aDMan(1→3)]bDMan(1→4)bDGlcNAc(1→4)[aLFuc(1→6)]bDGlcNAc(1→)PROC-159
G44	N228	M9	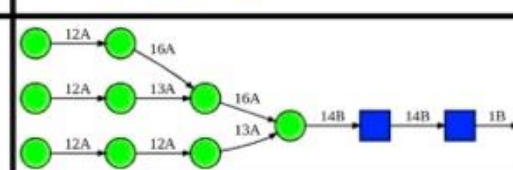	aDMan(1→2)aDMan(1→6)[aDMan(1→2)aDMan(1→3)]aDMan(1→6)[aDMan(1→2)aDMan(1→2)aDMan(1→3)]bDMan(1→4)bDGlcNAc(1→4)bDGlcNAc(1→)PROC-228
G45	N270	A2	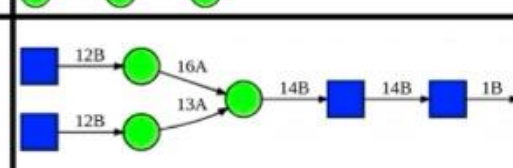	bDGlcNAc(1→2)aDMan(1→6)[bDGlcNAc(1→2)aDMan(1→3)]bDMan(1→4)bDGlcNAc(1→4)bDGlcNAc(1→)PROC-270
G46	N319	FA3G3S1	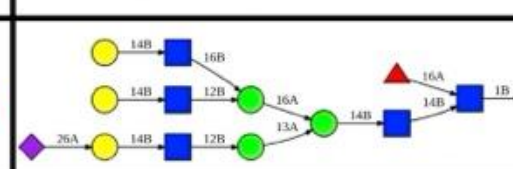	aDNeu5Ac(2→6)bDGal(1→4)bDGlcNAc(1→2)aDMan(1→3)[bDGal(1→4)bDGlcNAc(1→6)[bDGal(1→4)bDGlcNAc(1→2)]aDMan(1→6)]bDMan(1→4)bDGlcNAc(1→4)[aLFuc(1→6)]bDGlcNAc(1→)PROC-319
G47	N331	FA2	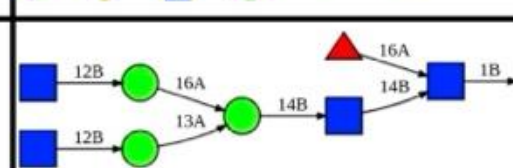	bDGlcNAc(1→2)aDMan(1→6)[bDGlcNAc(1→2)aDMan(1→3)]bDMan(1→4)bDGlcNAc(1→4)[aLFuc(1→6)]bDGlcNAc(1→)PROC-331
G48	N358	FA3G3S1	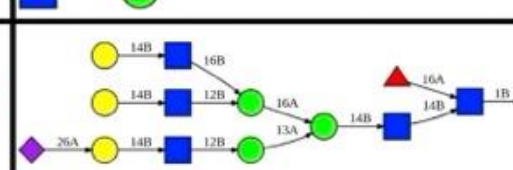	aDNeu5Ac(2→6)bDGal(1→4)bDGlcNAc(1→2)aDMan(1→3)[bDGal(1→4)bDGlcNAc(1→6)[bDGal(1→4)bDGlcNAc(1→2)]aDMan(1→6)]bDMan(1→4)bDGlcNAc(1→4)[aLFuc(1→6)]bDGlcNAc(1→)PROC-358
G49	N590	M5	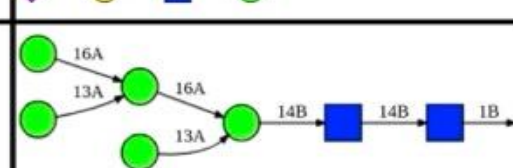	aDMan(1→6)[aDMan(1→3)]aDMan(1→6)[aDMan(1→3)]bDMan(1→4)bDGlcNAc(1→4)bDGlcNAc(1→)PROC-590
G50	N603	FA2	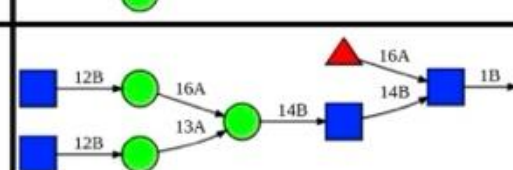	bDGlcNAc(1→2)aDMan(1→6)[bDGlcNAc(1→2)aDMan(1→3)]bDMan(1→4)bDGlcNAc(1→4)[aLFuc(1→6)]bDGlcNAc(1→)PROC-603
G51	N692	M5	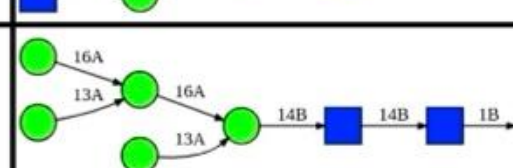	aDMan(1→6)[aDMan(1→3)]aDMan(1→6)[aDMan(1→3)]bDMan(1→4)bDGlcNAc(1→4)bDGlcNAc(1→)PROC-692
G52	N700	M6	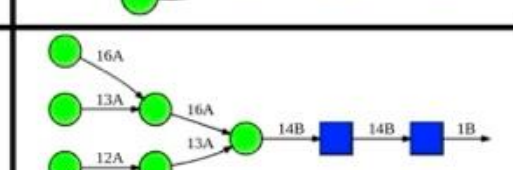	aDMan(1→6)[aDMan(1→3)]aDMan(1→6)[aDMan(1→2)aDMan(1→3)]bDMan(1→4)bDGlcNAc(1→4)bDGlcNAc(1→)PROC-700
G53	N784	M5	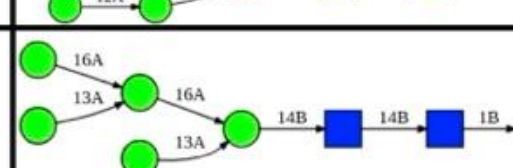	aDMan(1→6)[aDMan(1→3)]aDMan(1→6)[aDMan(1→3)]bDMan(1→4)bDGlcNAc(1→4)bDGlcNAc(1→)PROC-784
G54	N1057	M5	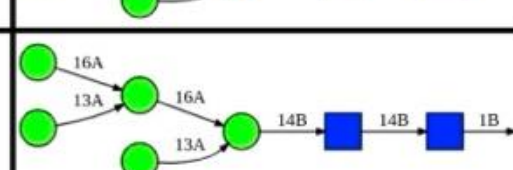	aDMan(1→6)[aDMan(1→3)]aDMan(1→6)[aDMan(1→3)]bDMan(1→4)bDGlcNAc(1→4)bDGlcNAc(1→)PROC-1057
G55	N1081	Hybrid G1S1	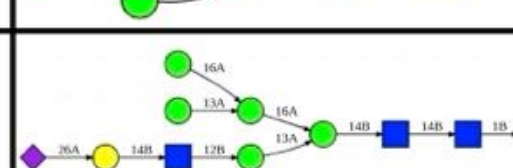	aDNeu5Ac(2→6)bDGal(1→4)bDGlcNAc(1→2)aDMan(1→3)[aDMan(1→6)[aDMan(1→3)]aDMan(1→6)]bDMan(1→4)bDGlcNAc(1→4)bDGlcNAc(1→)PROC-1081
G56	N1117	FA2	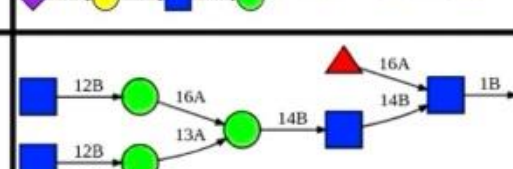	bDGlcNAc(1→2)aDMan(1→6)[bDGlcNAc(1→2)aDMan(1→3)]bDMan(1→4)bDGlcNAc(1→4)[aLFuc(1→6)]bDGlcNAc(1→)PROC-1117
G57	S311	O-glycan	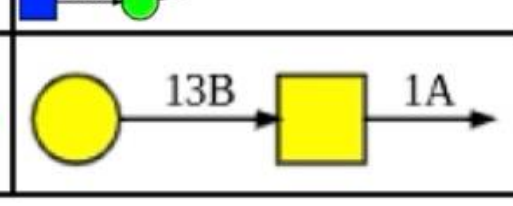	bDGal(1→3)aDGalNAc(1→)PROC-323

Table S4: Summary of average trimeric domain areas (Å²) for NTD, RBM, SD2, and FPPR in SHC014 WT and mutans. Values represent mean ± standard deviation across three independent 1-μs simulation replicates.

Variant	Domains			
	NTD (A^2)	RBM (A^2)	SD2 (A^2)	FPPR (A^2)
WT	2772.9 ± 36.0	661.3 ± 46.0	1640.1 ± 21.9	1233.1 ± 42.9
F294L	2766.8 ± 49.5	667.3 ± 7.5	1649.0 ± 15.0	1262.9 ± 38.7
A835D	2746.0 ± 40.1	674.7 ± 15.4	1626.5 ± 38.1	1380.8 ± 57.9
DM	2716.4 ± 29.3	663.2 ± 6.5	1670.4 ± 37.7	1351.8 ± 23.4

Table S5: Statistical analysis of FPPR trimeric area differences across SHC014 spike variants. One-way ANOVA results ($F = 8.2078$, $p = 0.0080$) indicate overall differences among variants. Pairwise comparisons were performed using Welch's t-test, and corresponding p-values are reported.

FPPR One Way ANOVA: F = 8.2078; p-value = 0.0080	
Variant	FPPR Domain (T-test p-value)
WT vs F294L	0.4225
WT vs A835D	0.0273
WT vs DM	0.0231
F294L vs A835D	0.0506
F294L vs DM	0.0366
A835D vs DM	0.4878
