# Supplementary material for: Structural dynamics and allosteric communication of a SARS-like bat coronavirus spike glycoprotein
Source: Biophys J. 2026 Apr 22;125(16):4420–36. doi: 10.1016/j.bpj.2026.04.023 (PMC13507448; doi:10.1016/j.bpj.2026.04.023)
Supplement: Supplementary file 2 [file mmc7.pdf]

Biophysical Journal
Article

Structural dynamics and allosteric communication of a SARS-like bat coronavirus spike glycoprotein

Toheeb A. Balogun,[1] Fiona L. Kearns,[1,5] Carla Calvó-Tusell,[1] Alexandra L. Tse,[2] Cory M. Acreman,[3,6] Lorenzo Casalino,[1] Gorka Lasso,[2,7] Emily Happy Miller,[2,4] Kartik Chandran,[2] Jason S. McLellan,[3] and Rommie E. Amaro[1,*]
[1]Department of Molecular Biology, University of California, San Diego, La Jolla, CA, USA; [2]Department of Microbiology & Immunology, Albert Einstein College of Medicine, Bronx, New York, NY, USA; [3]Department of Molecular Biosciences, The University of Texas at Austin, Austin, TX, USA; and [4]Department of Medicine, Albert Einstein College of Medicine, Bronx, New York, NY, USA
[5]Present address: Institute of Physical Chemistry, University of Freiburg, Freiburg, Germany
[6]Present address: Division of Biology and Biological Engineering, California Institute of Technology, Pasadena, CA, USA
[7]Present address: Division of Infection and Immunity, University College London, London, UK

ABSTRACT SARS-like bat coronaviruses (CoVs) pose ongoing public health risks due to their zoonotic potential, making it important to understand the molecular pathways that drive their evolution. We recently showed that SHC014-CoV can infect human cell lines in an angiotensin-converting enzyme 2 (ACE2)-dependent manner after acquiring two spike ectodomain mutations (F294L and A835D). However, how the wild-type (WT) SHC014 spike differs dynamically from these mutants remains unclear. Here, we built fully glycosylated ectodomain models of WT and three mutants (F294L, A835D, and the double mutant [DM]) and performed triplicate 1-μs all-atom molecular dynamics (MD) simulations for each variant. The two mutations exhibit epistasis, altering structural rearrangements relative to the WT. Notably, the DM receptor-binding domain (RBD) begins sampling the open conformation in our conventional MD. At the atomic level, the DM spike mitigates the dense negative packing introduced by A835D through a salt-bridge network, while F294L disrupts π-mediated interactions, together enhancing RBD opening propensity, which is critical for viral entry. Increased flexibility of the subdomain-2 "620-loop" further modulates DM RBD openness. Dynamical network analysis identified three allosteric communication pathways. In WT and F294L, "pathway 1" forms the baseline route linking the 620-loop to the RBD, whereas in A835D and the DM, it extends to the fusion peptide proximal region (FPPR), reshaping long-range communication. "Pathway 2" is conserved across variants but is most prominent in WT and F294L. "Pathway 3" appears only in A835D and the DM, compensating for reduced communication along pathway 2. Overall, this work provides an atomistic perspective on SHC014 molecular adaptation during host-to-host transmission and highlights mechanistic features that may inform future therapeutic and pandemic-preparedness efforts.

SIGNIFICANCE Bat coronaviruses are an important source of future pandemic threats, but we still know little about how small genetic changes help them infect humans. In this study, we used detailed computer simulations to watch how tiny mutations in a bat coronavirus spike protein change its motion and shape. We found that two specific mutations work together to make the spike more likely to open, a step required for the virus to enter human cells. By revealing how these molecular changes increase infection potential, our work helps improve understanding of coronavirus evolution and may guide strategies to prepare for future outbreaks.

INTRODUCTION

Bats harbor SARS-like coronaviruses (SL-CoVs) with zoonotic potential that continue to pose emerging viral threats to human health.[1] There have been multiple outbreaks of CoVs, including severe acute respiratory syndrome CoV (SARS-CoV), Middle East respiratory syndrome CoV (MERS-CoV), and SARS-CoV-2, with their origins hypothesized to stem from bats.[2,3] Several SL-CoVs have been identified[4–7] in nature, such as RatG13, RpYN06, BANAL-52, RmYN02, and WIV1, that share close genome identity with SARS-CoV-2, the human infectious agent of the COVID-19 pandemic. Furthermore, various studies have shown that the spike protein of certain bat CoVs can

Submitted November 27, 2025, and accepted for publication April 20, 2026.
*Correspondence: ramaro@ucsd.edu
Editor: B. Montgomery Pettitt.
https://doi.org/10.1016/j.bpj.2026.04.023
© 2026 The Author(s). Published by Elsevier Inc. on behalf of Biophysical Society.
This is an open access article under the CC BY license (http://creativecommons.org/licenses/by/4.0/).

utilize the human angiotensin-converting enzyme 2 (ACE-2) and its orthologs for cell entry, thereby priming SL-CoV to mediate infections in humans.[8–12]

A metagenomics study identified seven strains of SL-CoVs from Chinese horseshoe bats in Yunnan Province, China, including two novel pre-emergent strains called RsSHC014 and Rs3367, and elucidated their full-length genome sequences.[7] Although the authors were unable to recover a replication-competent RsSHC014, hereafter referred to as SHC014, they were able to characterize the first replication-competent SL-CoV, WIV-1-CoV, which is closely related to Rs3367. A follow-up study by Menachery and colleagues[13] showed that pseudotyped SHC014 is unable to utilize the human cellular receptor ACE2. Surprisingly, a chimeric virus containing the SHC014 spike glycoprotein (GP) was found to be replication competent by using multiple orthologs of the ACE2 receptor, and it can infect human airways and cause disease in mice, depicting SHC014's zoonotic potential to cause spillover events from bats to other mammals, including humans.

In SARS-CoV, SARS-CoV-2, and SHC014, the spike GP (Figure 1) is a class 1 fusion homotrimer protein that covers the surface of CoVs and plays a key role in viral entry.[14,15] Furin cleaves the transmembrane spike protein at the polybasic recognition site (PRRAR) known as the furin cleavage site (FCS). Cleavage at the FCS splits the spike sequence into two subunits, the S1 and the S2.[16–21] The S1 subunit consists of the N-terminal domain (NTD) and a receptor-binding domain (RBD), which contains the receptor-binding motif (RBM), the region that binds directly to ACE2 and mediates host cell entry.[22,23] S2 is responsible for the fusion of viral and host cell membranes and comprises the fusion peptide (FP), two heptad repeats (HR1 and HR2), a transmembrane domain (TM), and a palmitoylated cytoplasmic tail (CT) (Figure 1A).[15,24] Several studies have elucidated the structures of the SARS-CoV-2 spike protein in various conformational states.[25–28] Such SARS-CoV-2 spike conformational states are characterized by the number and degree of "up" or exposed RBDs: the closed spike conformation being one in which all three of the spike RBDs are "down" or highly shielded, and 1-up, 2-up, and 3-up states

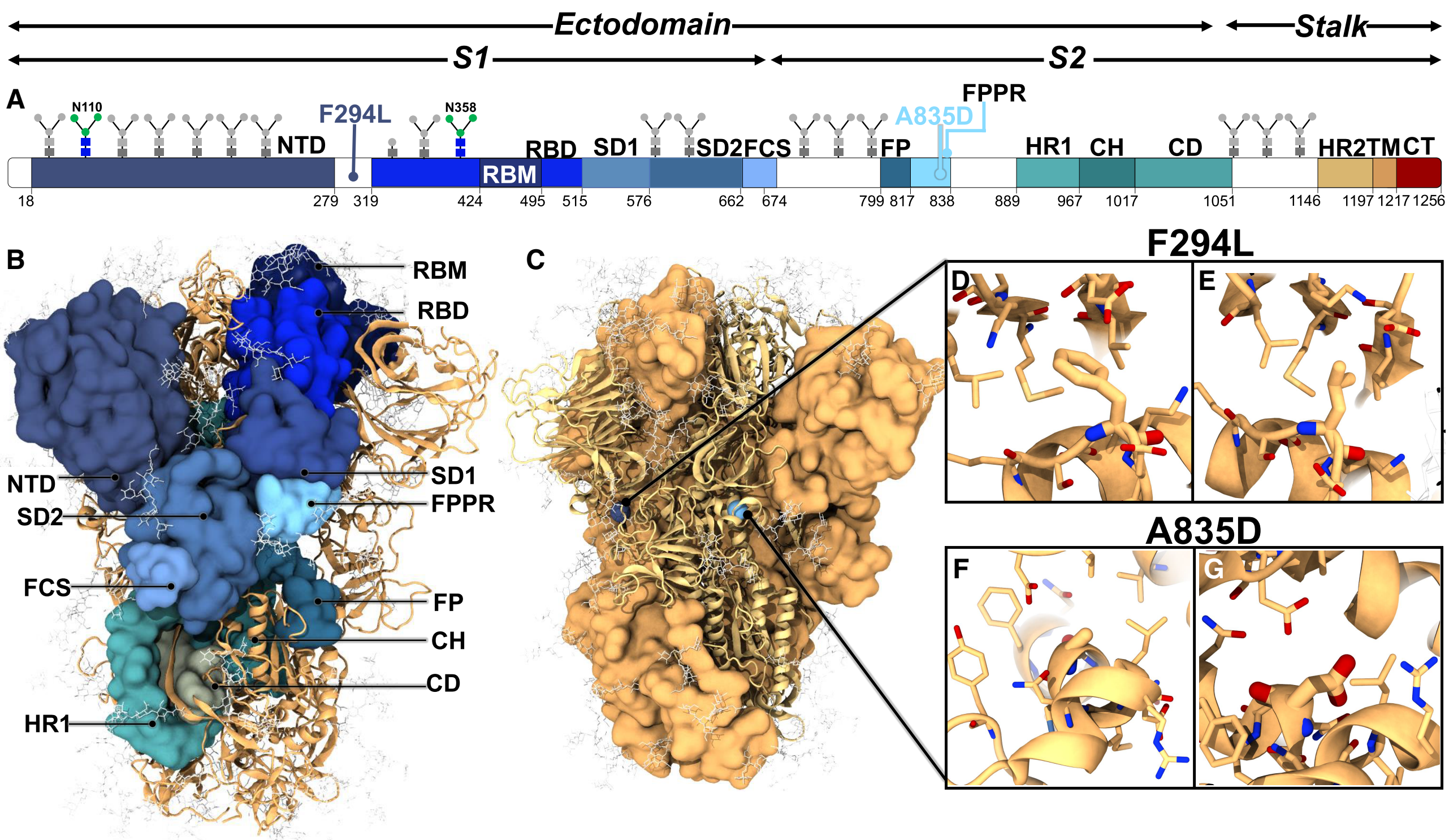

Figure 1. Modeling and system overview of the SHC014 spike GP. (A) Schematic of spike domains in a one-dimensional sequence of the spike protein, including the F294L and A835D mutations. Glycans at N110 and N358 (corresponding to N370 in SARS-CoV-2) are present in the SHC014 spike but are not found in the human SARS-CoV-2 spike. Domains are defined as such: ectodomain (18–1124), stalk (1125–1256), S1 (18–668), S2 (669–1256), N-terminal domain (NTD; 18–279), receptor-binding domain (RBD; 319–515), receptor-binding motif (RBM; 424–495), subdomain 1 (SD1; 515– 576), subdomain 2 (SD2; 577–662), furin cleavage site (FCS; 663–674), fusion peptide (FP; 799–817), fusion peptide proximal region (FPPR; 818–838), heptad repeat 1 (HR1; 889–967), central helix (CH; 968–1017), connecting domain (CD; 1018–1051), heptad repeat 2 (HR2; 1146–1197), transmembrane domain (TM; 1197–1216), and cytoplasmic tail (CT; 1217–1256). (B) The three-dimensional structure of the closed spike ectodomain highlights domains depicted in (A). Glycans are shown in light gray licorice. (C) Positions of F294L and A835D mutations are shown in dark blue and cyan, respectively, on the spike ectodomain structure. (D–G) Amino acid residues within 5 Å of the F294L and A835D mutations.

indicate conformations with one, two, or three up RBDs, respectively.[27,29,30] In the down state, the RBM within an RBD is highly shielded by neighboring protein surfaces and N-linked glycans; thus, down RBDs are considered to be inaccessible for ACE2 binding.[31] In an up or open state (more pronounced up states), the RBM is unshielded and can bind to ACE2.[27,30–32] During RBD opening, several other domains are known to also move including the NTD, two S1 subdomains (SD1 and SD2), and the FP proximal region (FPPR), as well as several glycans (Figures 1A and 1B).[17,33,34] Glycans are carbohydrate-based post-translational modifications anchored either to asparagines (N linked) or serines/threonines (O linked) that cover the spike GP, aiding in host cell immune evasion.[25,26,35–38] Our group has previously elucidated the structural role of several N-linked glycans, particularly those at positions N165 and N234, in stabilizing the human SARS-CoV-2 spike's RBD in the up position.[31] Similarly, we have previously described the gating role of the N343 glycan in facilitating RBD opening.[30] Therefore, interactions between spike subunits and glycans can modulate the conformational plasticity of spike RBDs, impacting accessibility to the ACE2 host cell receptor and therefore driving membrane fusion and viral entry.

Previous studies have demonstrated that mutations within the SARS-CoV-2 spike RBD, such as N501Y and T372A, impact infectivity and host-to-host transmission by modulating RBD conformations and ACE2 binding affinity.[39–44] Notably, a distal mutation far from the RBD, known as the D614G mutation (located ~75 Å from the RBD's center of mass in closed state), was associated with increased infectivity and rapidly became the predominant circulating strain during early stages of the COVID-19 pandemic.[28,45–49] Accumulating evidence predicts that the D614G mutation disrupts a key salt bridge to K854 within the neighboring chain's S2 FPPR, thereby enhancing the transition of the RBD from a closed to an up/open state.[28,50]

We have recently reported how a novel point mutation in the NTD (F294L) (Figures 1C–1E) of the SHC014 spike protein increases viral infectivity.[51] We further identified a second mutation in SHC014 spike's FPPR region (A835D) (Figures 1C, 1F, and 1G), which also enhances spike accessibility to ACE2 binding and increases viral entry into human host cells.[51] However, the molecular mechanism by which these two mutations alter SHC014 viral infectivity in human cells remains incompletely understood, which is the central goal of this current work. Herein, we investigated the conformational plasticity of the glycosylated SHC014 spike ectodomain using multiple μs-long, conventional molecular dynamics (MD) simulations. Our atomistic simulations reveal how F294L and A835D mutations led to structural rearrangements to enhance RBD opening by altering allosteric communication pathways. Altogether, our work deciphered mechanistic insights into the dynamics of an SL-CoV spike GP, the SHC014 spike GP, which may be used to understand species spillover events and the development of effective pandemic-preparedness countermeasures.

MATERIALS AND METHODS

Model system construction

The spike protein of SHC014 is a large, glycosylated trimeric structure with its ectodomain spanning residues 18–1124, which comprises the S1 and S2 subunits (Figure 1A).[14,15] We have previously reported the experimental structure of the SHC014 spike protein (PDB: 9CAS), as described in Tse et al.,[51] with all three RBDs in closed conformations used herein to construct the ectodomain, fully glycosylated models of the wild-type (WT) and three mutant spikes. Specifically, four models were constructed, including the WT, two single mutants—the first mutation is in the NTD, where phenylalanine at position 294 was mutated to leucine (F294L), and the second mutation is in the FPPR, where alanine was mutated to aspartate (A835D)—and a double mutant (DM; F294L + A835D). Thus, we employed a multi-step modeling approach to construct a complete model of the SHC014 spike ectodomain with fully resolved loops, N- and O-linked glycans, and reversion of stabilizing mutations.

Missing loops modeling

The resolved cryoelectron microscopy (cryo-EM) structure of SHC014 GP contains several unresolved sequence gaps corresponding to flexible loops in the NTD, SD2, and FPPR, resulting in 39 unresolved loops in the trimeric structure (Figure S1A). To generate a complete ectodomain WT construct, missing gaps were modeled as disordered loops using various protein structure prediction tools, including the Swiss Model,[52] AlphaFold2,[53] Prime,[54] and MODELLER.[55] The NTD (residues 18–279) contains several unresolved flexible loops (9 per monomer, 27 in the trimeric structure). Considering the poor density of these unresolved loops, we elected to employ Swiss Modeling, AlphaFold2, and Prime Homology Modeling and achieved consensus among the modeling techniques for these NTDs. Missing loops within the NTD were then grafted from the Swiss Model prediction—which was, again, highly similar to Prime- and AF2-predicted models—onto the cryo-EM structure in chains A, B, and C each (Figure S1B). To model the SD2's unresolved loops, 5 complete SD2 models per monomer were independently generated using MODELLER, from which the top per-chain SD2 model was chosen based on the *Z* score and taking care to avoid overlap with other domains. The resulting refined loops in each top-predicted SD2 model were visually inspected and grafted to the resolved cryo-EM structure (Figure S1C). To achieve the most accurate model of the FPPR, we identified the unresolved sections of the FPPR sequence from UniProt (residues 818–831) and predicted the structure of

this loop via homology modeling with Swiss Model using the human SARS-CoV-2 spike GP as a template structure (PDB: 7JJI) for the SHC014 sequence in this region[56] (Figure S1D). The complete FPPR-predicted region was then, as with the NTD, grafted onto the cryo-EM resolved regions of the SHC014 structure. In all cases of grafting, the joined positions between grafted predicted loops and resolved cryo-EM were visually inspected to ensure there were no clashes, overlaps, or ring penetrations. Notably, the S1/S2 FCS was modeled in an uncleaved state. UniProt and a comparison with the human SARS-CoV-2 spike system structure were used to identify necessary disulfide bonds (15 per monomer) as listed in Table S1.

Glycosylation/protonation/solvation/neutralization

SHC014 spike protein ectodomain glycosites were predicted using the NetNGlyc webserver[57] and the N-Glycosite tool[58] and further confirmed by previously reported SHC014 site-specific glycan analysis.[59] They comprise 18 N-glycan sequons (N-X-S/T) per monomer, including N110 and N358, but are not present in SARS-CoV-2. N110 and N358 correspond to N110 and N370, respectively, in the SARS-CoV-2 glycosylation profile. Our modeled constructs are fully N-/O glycosylated following a similar human glycoprofile to that used by Casalino et al.,[31] which was consistent with Watanabe et al.[25] Detailed glycosylation profiles of SHC014 GP per chain simulated herein are shown in Table S3. In summary, 57 glycans (18 × 3 monomer N-glycans and 1 × 3 monomer O-glycans) were incorporated into the WT spike system. Any protein/glycan clashes or ring penetration in each construct were remedied using VMD's manual structure manipulation tool, Molefacture, and VMD's site-specific minimization and equilibration MD simulation tool, AutoIMD.[60]

System preparation

It is important to note that in this study, the WT S1/S2 site was modeled in an uncleaved form. Consistent with Casalino et al.,[31] the S1 and S2 subunits within each protomer have been assigned to the same chain (referred to as A/B/C). Glycans were assigned segnames from G1 to G57, as reported in Table S3. Protonation states for all titratable residues (Asp, Glu, Tyr, Lys, Cys, and Arg) were assigned using PROPKA[61] at pH 7.4, and His states were assigned with Schrodinger's Protein Preparation Wizard tool.[62] All Asp and Glu were determined to be deprotonated and negatively charged, all Cys were determined to be involved in disulfide bonds and thus not titratable, all Tyr were determined to be protonated and neutral, and all Lys and Arg were determined to be protonated and positively charged. His numbered 38, 54, 59, 492, 662, 1041, and 1066 were modeled as neutral and singly protonated on the δ-nitrogen (HSD). His numbered 196, 612, 642, 1031, and 1042 were modeled as neutral and singly protonated on the ε-nitrogen (HSE). Protein structure files for the WT protein structure were generated via VMD's psfgen module according to CHARMM36 all-atom additive force fields for proteins and glycans.[63,64] The mutate command in VMD's psfgen module[65] was then used to modify the WT structure to F294L, A835D, and DM spike structures. The WT construct was fully solvated with explicit water molecules described using the TIP3P model[66] and neutralized with a 150 mM concentration of Na and Cl ions. See Table S1 for complete details of atom counts and system sizes.

MD simulations

The solvated and neutralized WT, F294L, A835D, and DM ectodomain models were minimized and briefly equilibrated in NAMD3[67] using the protocol outlined as follows. For all the following described protocols, system temperature and pressure were controlled with a Langevin thermostat[68] and a Langevin barostat,[69] respectively. Periodic boundary conditions were used to model infinite bulk conditions, and particle-mesh Ewald[70] was used to handle long-range electrostatic interactions. The SHAKE algorithm[71] was employed to fix covalent bonds involving all hydrogen atoms. van der Waals interactions and electrostatic interactions were computed with the following cutoff scheme: cutoff distance of 12 Å, pair list generated for all pairs within 13.5 Å, and switching function implemented for all pairs between 10 and 12 Å apart. Nonbonded interactions were excluded for bonded atoms within 1–4 bonds of each other.

Minimization and heating

First, waters and ions were subjected to 10,080 steps of conjugate gradient minimization, and protein and glycan atoms were held fixed by Lagrangian multiplier constraints. Next, an MD heating protocol was used to gradually introduce the systems to biologically relevant temperatures: protein and glycan atoms were held fixed during this step (Lagrangian multiplier constraints), and starting at 10 K, the temperature was incremented by 25 K every 10,080 MD steps (1 fs/step) until 310 K. At 310 K, 1 ns of short equilibration MD was performed.

Volume and pressure equilibration

Following the heating protocol, Lagrangian constraints were removed from protein and glycan atoms, and a short (2,520 steps) conjugate gradient minimization was performed wherein all water and ion atoms were allowed to freely move and protein and glycan atoms were lightly restrained with a force constant $k = 1$ kcal/mol/Å^2. Following this short minimization, 0.5 ns (2 fs/step) of pressure and volume equilibration were performed, wherein the periodic box was allowed to fluctuate in size but protein and glycan atoms

were still subjected to the light restraint (k = 1 kcal/mol/$Å^2$).

Free NVT equilibration

Following pressure and volume equilibration, the periodic box was held fixed (useFlexibleCell set to no, useConstantArea set to no) for ~1 ns (2 fs/step) of free equilibration in which all restraints and constraints were removed from protein and glycan atoms. See Table S2 for total simulation time.

Analysis methods

Protein root-mean-square fluctuations

The root-mean-square fluctuations (RMSFs) for the WT and mutant SHC014 spike GPs were calculated using the MDAnalysis[72,73] RMSF module. Each trajectory was aligned onto the initial coordinates using the C_α atoms of the entire protein to remove global translation and rotational degrees of freedom. We further calculated the RMSF of the glycans by aligning the glycosylated trajectory to the reference frame using the heavy atoms of the protein glycosite (i.e., Asn for N-linked glycans and Ser or Thr for O-glycans). After this, we calculated the RMSF for all C1 atoms in the glycan segment analogous to C_αs in protein segments and averaged those C1 RMSFs per frame over the whole simulation.

Residue-to-residue contact distance measurements

Contact analysis was carried out for every frame in each simulation across all chains of each variant using the distance module of MDAnalysis.[72,73] The minimum distance between heavy atoms was used to calculate distances for the following: A/D835 to D555, A/D835 to R830, R830 to D555, F/L294 to D275, F/L294 to K266, F/L294 to K288, R830 to N590 glycan, and R830 to N270 glycan. Only side chain-to-side chain contacts were considered for contact analysis involving F/L294 and its neighboring residues, as backbone atoms are already very close to one another.

RBD-core distances

Per-frame distances between the RBD in SHC014 spike and the spike core were computed using MDAnalysis[72,73] by measuring the distance between the center of mass of the RBD C_α atoms (residues 319–515) and that of the spike's central helices (C_α atoms from residues 968–1017). These calculations were performed per replica trajectories of each variant (WT, F294L, A835D, and DM).

620-core distances

As described above, we carried out a similar analysis between the center of mass of the 620-loop's C_α atoms (residues 601–627), and the spike core was computed using MDAnalysis[72,73] to determine the flexibility of the 620-loop.

Trimeric area calculation for NTD, RBD, RBM, SD2, and FPPR

MDAnalysis[72,73] was used to calculate distances and areas between trimeric domains of interest in the SHC014 spike protein. The calculation was done by selecting the center of mass of each domain of interest for each chain (A, B, and C), determining the distance between that particular domain per chain, and then calculating the area using the three-sided area formula. These calculations were performed per frame over all frames in all simulations and per variant. The residue numbers for each domain are as follows: NTD (18–279), RBD (319–515), RBM (424–495), SD2 (577–680), and FPPR (818–838).

ASA

The solvent-accessible surface area (SASA) was computed using the measure sasa command in VMD,[65] which implements the Shrake and Rupley method, with in-house Tcl scripts. A probe radius of 7.2 Å was used to assess the surface accessibility of the SHC014 spike protein. To estimate glycan shielding, the ASA of the protein with glycans was subtracted from that of the same protein lacking glycans. These calculations were carried out at 1-ns intervals across the trajectories, and the results were averaged over all replicas, with their respective standard deviations.

Dynamical network analysis using WISP

To understand the allosteric communication that may influence conformational changes in the RBD of SHC014 spike variants, we employed a dynamical network analysis approach using data from our conventional MD simulations. Then, we computed distance and cross-correlation matrices using the cpptraj module of Amber,[74,75] considering the C_α atoms of protein residues. The cross-correlation matrix, calculated using Pearson's correlation coefficient, quantifies residue pair correlations by measuring the linear relationship between atomic motions. The correlation coefficient values range from -1 to 1, where $+1$ indicates residues moving in the same direction (fully correlated motion), 0 represents uncorrelated motion, and -1 denotes residues moving in opposite directions (fully anti-correlated motion).

By utilizing the weighted implementation of suboptimal paths (WISP) method,[76] we constructed a network representation of the spike protein by mapping the N residues into N nodes of a weighted graph. Each residue was represented as a node, centered on its C_α, and an edge was established between two residues with a mean distance cutoff of 6 Å throughout the simulation. The edges were weighted based on the normalized cross-correlation matrix values using the transformation ($d_{ij} = -\log |C_{ij}|$), where Cij is the cross-correlation coefficient between residues i and j and

dij represents a functional correlation-based distance. This transformation ensures that strongly correlated residue pairs have shorter edge distances, reflecting the probability of information transfer based on their correlation. Dijkstra's algorithm,[77] implemented in the NetworkX Python Library, was employed to uncover critical allosteric communication pathways. This algorithm determines the shortest paths between residues by ranking edges with shorter distances, indicating the most frequently used and highly correlated pathways. The method identifies the highest-contributing edges to allosteric communication and the residues that are key to network connectivity. The top 1,000 edges and nodes in the dynamical networks that contribute to the allosteric pathways were visualized using VMD.[65] Finally, we extracted the edge-weighted domain-domain pairs averaged over all three chains of the spike trimer to map continuous pathways and reveal how the F294L and A835D mutations reshape the communication routes.

RESULTS AND DISCUSSION

To investigate how the F294L and A835D mutations rescue spike-ACE2 binding and SHC014 viral infectivity in human cell lines, we constructed fully glycosylated ectodomain models of the SHC014 spike GP for the WT, two single mutants (F294L and A835D), and the DM (F294L + A835D) (see materials and methods for model construction details and Figures S1A–S1E for modeling results). Following model construction, the WT and mutant spike ectodomains were equilibrated, and triplicate 1-μs all-atom production MD simulations were performed for each system (see Tables S1 and S2 for simulation details, Table S3 for the SHC014 glycosylation profile, and Figure S2 for root-mean-square deviation [RMSD] plots used to assess simulation stability).

Altered dynamics between SHC014 spike domains in WT and mutant strains

The spike RBD conformational landscape, including RBD opening pathways, correlates with intra- and inter-protomer motions such as distances between the three RBDs, distances between RBDs and neighboring NTDs, and motions of other SDs with the spike protein.[78] To delineate structural movement within the SHC014 spike, we analyzed the RMSF profile of the SHC014 spike protein across all the simulated models. We found that global RMSF profiles were broadly similar across all SHC014 spike variants (Figure 2A). However, we observed pronounced fluctuations within NTDs, RBDs, RBMs, SD2s, FCSs, and FPPRs. Based on the overall distribution of per-residue flexibilities (Figure 2A), we identified the domains with the

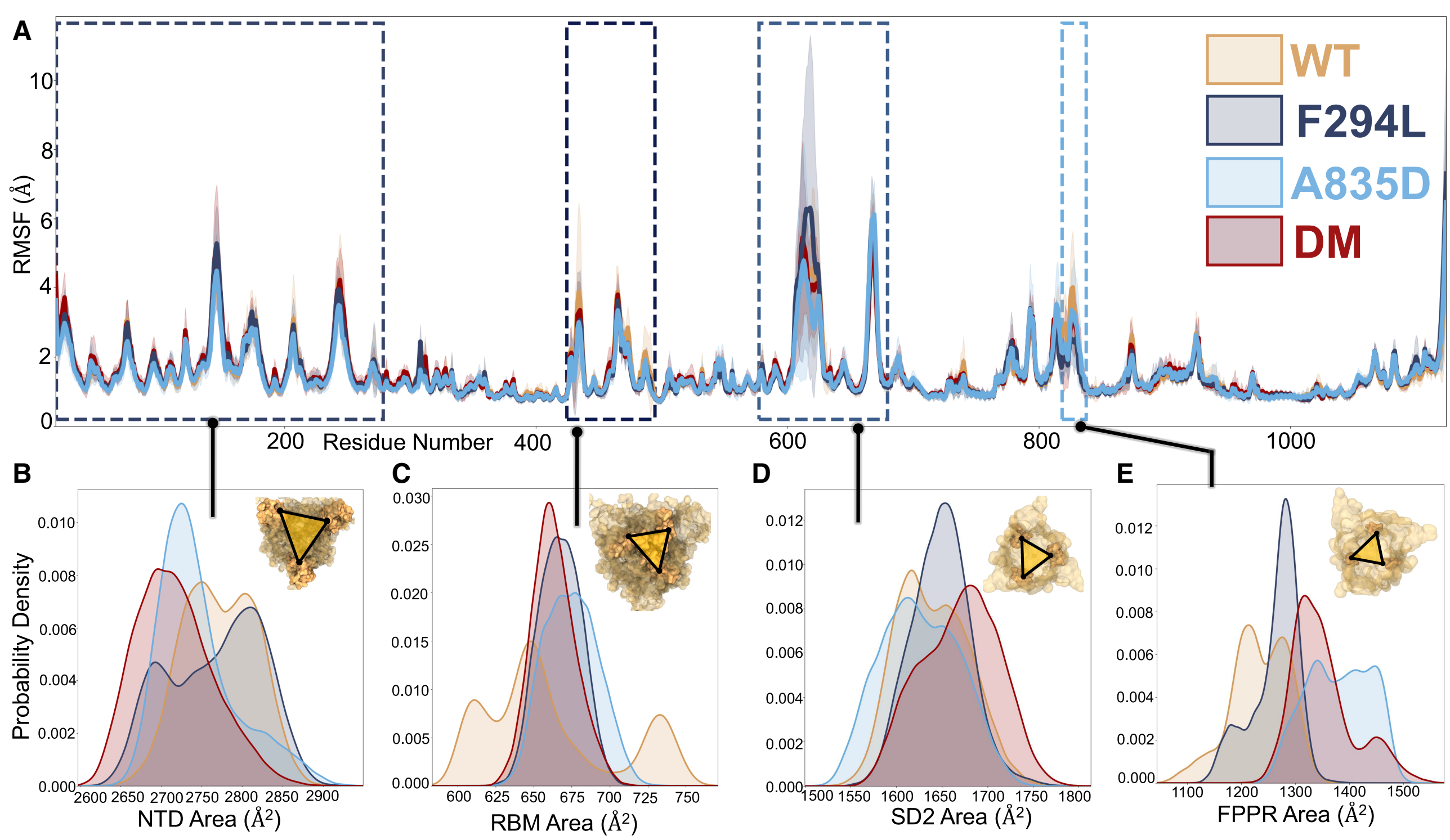

Figure 2. Characterization and flexibility of different domains of SHC014 spike GP. (A) Root-mean-square fluctuations of the simulated systems: WT, F294L, A835D, and DM. (B–E) Distribution of trimeric areas for domains (NTD, RBM, SD2, and FPPR) exhibiting pronounced flexibility. The distributions represent the concatenation of triplicate trajectories for a given domain for each variant. The *y* axis denotes probability density, while the *x* axis represents the distribution of trimeric area packing around the spike core. The variants are shown as WT in gold, F294L in dark blue, A835D in cyan, and DM in red.

most noticeable flexibility for more detailed dynamical analysis to unravel how their local motion influences global spike dynamics.

To investigate the relative mobility of each domain of interest in the trimeric SHC014 spike, we identified the center of mass of each domain and tracked the area between each domain's center of mass (Figure S3). This metric indicates spatial breathing between each of the trimetric domains: a larger area indicating that domains are more widely spaced from one another and smaller areas indicating tighter packing toward the spike core. Tracking these areas throughout all simulations allows us to assess the degree of breathing between domains, as reflected in the widths of the distributions (Table S4, summary of average trimeric domain areas for NTD, RBM, SD2, and FPPR). Thus, trimeric area analysis provides insight into inter-protomer compactness and preorganization of the spike trimer, which influence premature S1 shedding and the number of functional spikes that reach target cells. Figure 2B shows the distributions of areas between NTDs for the WT and mutant spikes simulated in this study. WT SHC014 spike NTDs sample a bimodal distribution of inter-NTD areas, whereas F294L NTDs exhibit a broad multimodal distribution. A835D displays a narrower distribution with a right skew toward lower inter-NTD areas, while the DM spike samples a left-shifted, broad distribution compared to WT, F294L, and A835D. The left-shifted distribution observed for DM, along with the narrower distribution for A835D, may reflect increased inter-protomer packing of the NTDs, potentially contributing to the rigidity of the prefusion trimer and limiting premature S1 shedding.

Similarly, the WT SHC014 spike RBMs exhibit a markedly different distribution of areas compared to the mutants (Figure 2C). WT RBMs show a broad trimodal distribution with substantial variability between replicates, indicating sampling of multiple conformational states (Figures 2C and S4 for RBD). The heterogeneous conformational sampling observed in WT RBMs may suggest more loosely packed RBDs and reduced structural compactness, which could lead to earlier dissociation of S1-S2 interactions and premature shedding of the S1 subunit, thereby potentially reducing the number of functional spikes capable of facilitating infection. This is consistent with our previous experimental findings, in which WT exhibited the lowest infectivity level.[51] In contrast, all mutant spikes, particularly the DM, exhibit relatively narrow, unimodal distributions of RBM areas compared to the WT. In the prefusion spike trimer, compactness and preorganization of inter-protomer RBDs are important determinants of spike fitness and infectivity.[78,79] Thus, the unimodal and narrow RBM area distributions observed in the mutants indicate more tightly packed and preorganized RBDs, as well as increased structural rigidity of the prefusion trimer. This may limit premature S1 shedding and enhance S1-S2 interactions, thereby increasing spike incorporation on virions to drive viral entry. This aligns with our previous study showing that the SHC014 spike mutants are more infectious than the WT, with the DM exhibiting the highest infectivity. Previous studies have shown that the SARS-CoV-2 D614G variant increases viral spread by enhancing S1-S2 interactions, preventing premature S1 shedding, and increasing the proportion of functional spikes during host-to-host transmission.[28,48,50,80,81] Similarly, the SARS-CoV-2 Omicron spike achieved high transmissibility with its tightly packed RBD interfaces and enhanced inter-protomer interactions, thereby reducing early S1 dissociation and rigidifying the prefusion trimer.[78–80] Furthermore, Berger and Schaffitzel[82] showed that the SARS-CoV-2 spike has evolved to enhance structural compactness of the prefusion trimer to avoid premature inactivation while balancing infectivity.

We also observe that SD2 exhibits variant-dependent differences in motion. In the SARS-CoV-2 spike, SD2 is a structural SD within the S1 region of the spike ectodomain and encompasses two regions previously implicated in membrane fusion: the FCS and the 630-loop. RMSF analysis (Figure 2A) shows that residues within SD2 display sequence-dependent flexibility, a result described in more detail in later sections. Accordingly, we also monitored the extent of SD2 "breathing" in the SHC014 spike (Figure 2D). The WT, A835D, and DM spikes are composed of multiple states, while the F294L exhibits a narrower unimodal distribution. In SARS-CoV-2 spikes, the FPPR has been reported to adopt altered conformations as a function of RBD openness and potentially serves to stabilize the RBD in the closed conformation[27]; thus, we also tracked areas between trimeric FPPRs over all simulations and per variant. All spike structures exhibited dramatically different degrees of FPPR breathing relative to one another. All FPPR trimeric area distributions are multimodal, indicating multiple conformations (Figure 2E). Considering that the A835D mutation is located within the FPPR (Figures 1F and 1G), we expect local packing in this region to be altered due to this mutation. Indeed, significant differences in FPPR trimeric areas were observed across variants. Pairwise comparisons showed that A835D and DM exhibited significantly larger FPPR areas relative to WT. DM FPPR trimeric area also differed significantly from F294L, whereas no differences were observed between WT and F294L or between A835D and DM FPPR areas (Table S5, statistical analysis of FPPR trimeric areas).

Collectively, these domain-based data reveal that the F294L and A835D mutations drive altered inter-domain dynamics. In particular, we see that WT spike RBMs are far more flexible in trimeric areas relative to variant spikes. Previously, we reported that the WT SHC014 has low to no infectivity in human cells compared to the mutant strains.[51] The WT RBM's sampling multiple conformations compared to the other mutant strains could indicate premature S1 shedding contributing to the observed decreased infectivity, as we hypothesized previously.[51] Moreover, the observed altered dynamics of spike NTDs, SD2s, and

FPPRs per spike variant indicate this sequence mutations could be impacting inherent spike allosteric networks.

DM SHC014 spike demonstrates increased propensity for RBD opening

The initial starting structure of the SHC014 spike GP (PDB: 9CAS) used herein was resolved with all three RBDs in the down state, i.e., a "closed" spike conformation.[51] Before the spike can bind to human ACE2, at least one of the RBDs needs to emerge from the down/shielded state into the up and open states to reveal the RBM.[30] However, the SARS-CoV-2 spike's RBM, and the RBD in general, is highly antigenic, eliciting the largest humoral immune response relative to any other domain within the spike protein.[83] Thus, probabilistic tuning of spike RBMs in down versus up conformations has been hypothesized to provide a replication advantage by balancing binding affinity and cell membrane fusion with immune recognition.[84] Therefore, another factor of viral fitness and spillover to humans lies in the rate of RBD opening and resulting equilibrium distributions of down versus up RBDs. Thus, we sought to identify whether WT or variant SHC014 spikes could be observed to initiate RBD opening in our μs-long conventional MD simulations. Experimentally determined RBD opening rates are reported to occur on a seconds-long timescale *in vitro*.[85] Therefore, observation of spike RBD opening via conventional MD is likely intractable due to rare event sampling requirements, especially for such a large GP.[30] However, we can use observations from conventional MD to inform on the tendency of spike RBDs toward opening. To track RBD position relative to that of an open RBD, we computed the distance between the center of mass of RBD β sheet C_α atoms and the center of mass of CH's C_α atoms (also known as the "spike core") for each of three RBDs per spike and for every frame in all simulations. We herein refer to such values as "RBD-core distances" (Figure S5), in keeping with our previous work,[30,31] where larger and smaller values indicate increased or decreased displacement of the RBD from the spike core, respectively.

WT RBDs demonstrate a bimodal distribution with a primary high-probability conformation and a distinct low-probability left-hand tail (Figure 3A), in which the RBDs are tightly compacted toward the spike core in a "locked down" state. The F294L and A835D distributions overlap substantially with the RBD-core distances observed for the primary WT conformation. However, F294L exhibits a multimodal distribution within this range. The RBD-core distance of the SARS-CoV-2 spike structure[30,86] has been reported within the following ranges: closed

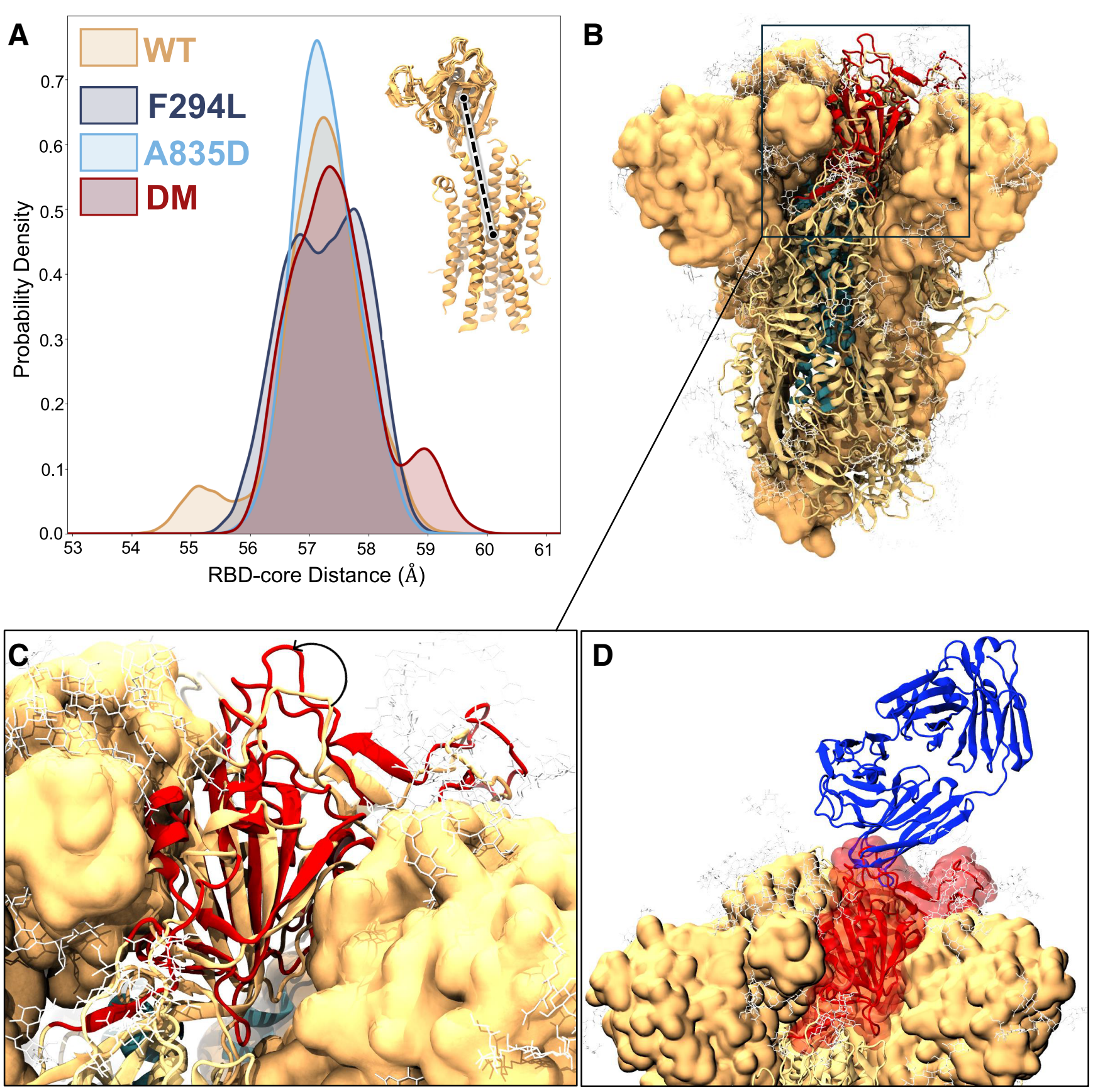

Figure 3. DM spike RBD initiates opening in conventional MD simulations. (A) RBD-core distance distribution calculated using the center of mass of the RBD to the central helices of the spike core. RBD-core distance thresholds: closed ≤ 57 Å, intermediate conformation (transition between closed and open) ~ 58–69 Å, and open state > 70 Å. The WT left-tail distribution represents the closed state, whereas the DM right-tail distribution represents sampling of intermediate conformations. (B and C) Close view of representative DM spike RBD conformations showing a modest increase in RBD opening. The curved arrow depicts the transition from the closed state to the intermediate conformation. WT is shown in gold and DM in red. (D) Complex of SARS-CoV-2 neutralizing antibody ADG2 (shown in blue) in complex with the DM spike RBD (shown in red).

state ≤ 57Å, intermediate (transition between closed and open) ~ 58–69 Å, and open state > 70 Å. Interestingly, the DM spike RBD samples a population of intermediate conformations with a slightly increased RBD-core distance, potentially exhibiting a modestly heightened propensity for opening (Figure 3A; see Figure S5 for the RBD-core distance time series). It has been shown that, for the SARS-CoV-2 spike to enter host cells, at least one RBD must transition from the closed state, exposing the RBM for receptor engagement. Therefore, sampling of intermediate conformations in SHC014 DM suggests that RBD exposure is likely needed for ACE2 binding. This probability is further supported by our previous experimental results, which show that DM and A835D increase viral infectivity in an ACE2-dependent manner. However, capturing large-scale RBD opening often occurs on timescales longer than those accessible to the conventional MD simulations used herein and may require extensive sampling or enhanced sampling methods. Our lab has previously characterized the gating role of the N343 glycan in facilitating RBD opening using weighted ensemble MD simulations.[30] Furthermore, the probable RBD opening in DM and A835D also renders the virus more susceptible to neutralizing antibodies (Figures 3B–3D). In our previous experimental study,[51] we reported that Adagio-2 (ADG2), an antibody that recognizes a conserved RBD epitope accessible only in the open spike conformation, neutralizes DM, A835D, and F294L, with minimal neutralization of WT. To further assess RBD accessibility, including the conserved ADG2 binding site and the effect of glycan shielding, we performed accessible surface area (ASA) calculations for the RBM and other domains using a probe radius of 7.2 Å. The ASA results indicate only subtle differences across variants (Figure S6). Altogether, we propose that the F294L and A835D mutations confer a fitness trade-off in SHC014-CoV by increasing RBD opening to facilitate cell entry while simultaneously enhancing susceptibility to neutralizing antibodies, thereby providing an avenue for countermeasure development to support pandemic preparedness efforts.

Mutant spike contact networks drive altered domain dynamics

We next sought to characterize the atomic-scale interactions affected by the F294L and A835D mutations and to predict how those contacts may impact spike domain motions, including RBD opening. Previous studies have shown that as the SARS-CoV-2 spike RBD transitions between down, up, and open conformations, several salt-bridging and hydrogen-bonding interactions form and break,[30] including within the FPPR.[86] As such, we identified key contacts between positions 294 and 835 and their neighboring amino acids and glycans, and we tracked such contacts (heavy atoms within 4 Å of one another) over the course of all simulations and for all studied spike variants (Figures 4 and S7–S15).

There are several Asp and Arg residues in the local region surrounding position 835, and we tracked contacts between these neighboring residues (Figures 4A–4G and S10–S12; Videos S1 and S2). We predicted in previous work that the introduction of another Asp in the FPPR region may result in marked rearrangement within that area to alleviate electrostatic clashes.[51] Our simulations have supported this hypothesis: D835 is very close to the neighboring chain's D555, which sits at the base of the SD1, close to the RBD (Figure 1B). We observed an ~20% increase in contact frequency between D835 and R830 relative to A835 to R830 contacts seen in WT and F294L variants (Figures 4F and S10). Moreover, we observe ~32%, ~26%, and ~18% increases in R830 to D555 contact frequency for the DM spike relative to WT, F294L, and A835D spikes, respectively (Figures 4G and S11). Interestingly, we see that although A835 or D835 and D555 are in close proximity in all simulations, with ~26%, ~29%, and ~32% contact frequency between A835 or D835 and D555 in WT, F294L, and A835D, respectively, there is a distinct decrease in D835 and D555 contact frequency for DM spikes to ~20% (Figures 4E and S12). To unravel the formation and persistence of the salt-bridge triad between R830-D555-D835, we computed the distance between the centers of mass of each residue throughout all simulations and for contacts spanning each of the protomeric interfaces. From these results, we see that 38% and 44% of simulation frames from A835D and DM spike variants, respectively, show tight salt-bridge triad formation (area <30 $Å^2$), which is absent in WT and F294L variants due to the presence of alanine at position 835 (Figure S13). These data suggest two potential mechanisms for alleviation of the densely packed negative charge: (1) recruitment of R830 to stabilize D835 and D555, as they neighbor each other, and (2) adjustment of RBD position to separate D835 and D555, as this was the only spike seen to begin initiation of RBD opening.

Around position 294, there are several positively charged Lys and negatively charged Asp residues (Figures 4H–4K; Videos S3 and S4). Because aromatic amino acids in this region could exploit cation-π or anion-π interactions for stability, we sought to track contacts between these charged residues and position 294 (Figures 4H–4N and S7–S9). Firstly, we see that contact between L294 and K266 is ~10%–15% more favorable than that between F294 and K266 (Figures 4L and S7). Secondly, we see that contacts between F294 or L294 and K288 are largely the same regardless of F/L at this position (Figures 4M and S8). Thirdly, we see that the A835D variant has a slightly decreased propensity for contact between F294 and D275 compared with the other variants (Figures 4N and S9).

Additionally, we observed protein-glycan contacts, particularly between R830, which mediates salt-bridge

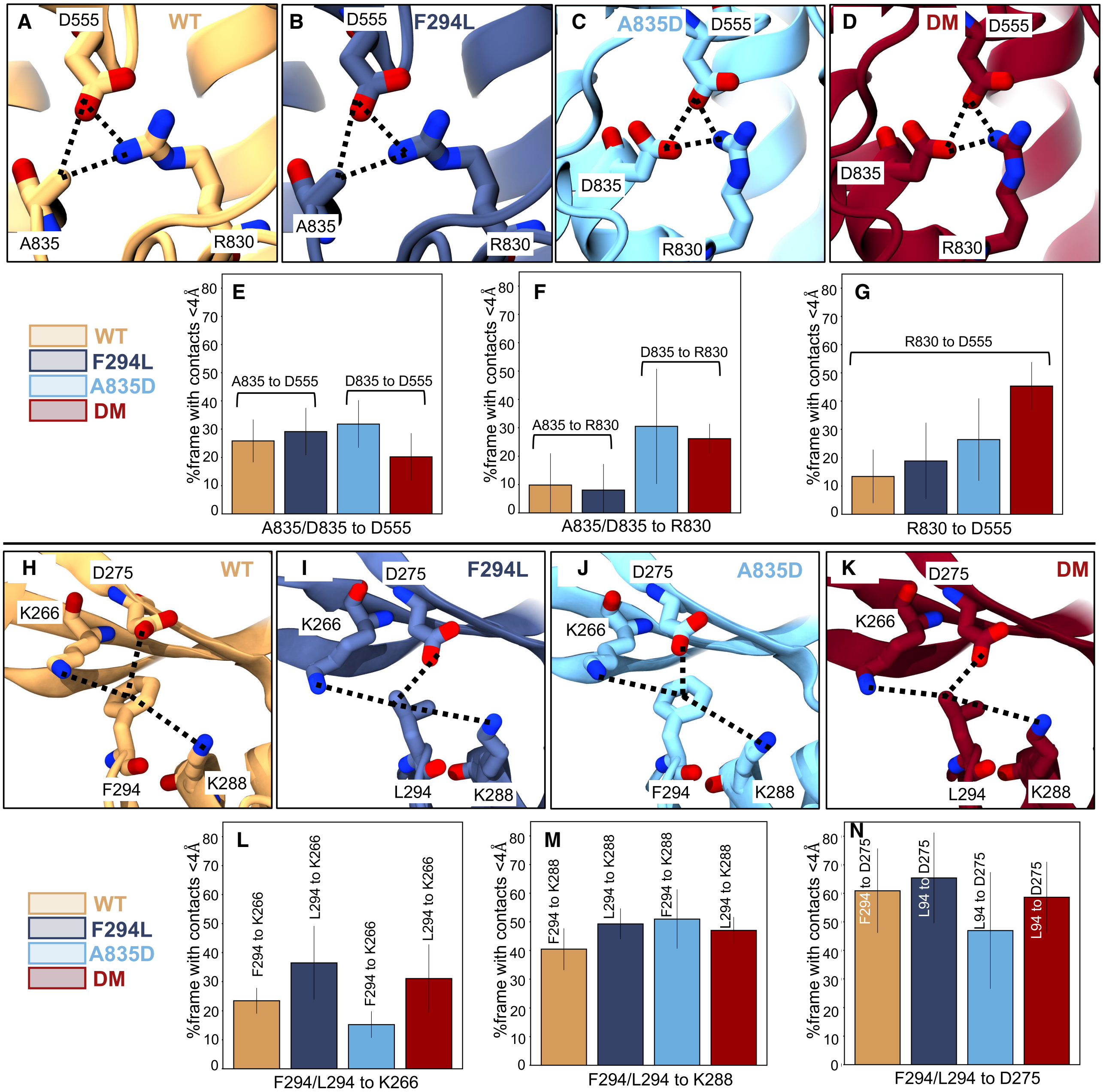

Figure 4. Contact analysis of atomic-level interactions in SHC014 WT and mutants. (A and B) Contacts between A835-D555-R830 in WT and F294L. (C and D) Salt-bridge interactions among D835, D555, and R830 in A835D and DM. (E–G) Contact frequency (<4 Å) between (E) A835 or D835 and D555, (F) A835 or D835 and R830, and (G) R830 and D555. (H) Double cation-π interactions among F294, K266, and K288 and anion-π interaction between F294 and D275 in WT. (I) Contacts among L294, K266, K288, and D275 in F294L. (J) Double cation-π interactions among F294, K266, and K288, and anion-π interaction between F294 and D275 in A835D. (K) Contacts among L294, K266, K288, and D275 in DM. (L–N) Contact frequency (<4 Å) between (L) F294 or L294 and K266, (M) F294 or L294 and K288, and (N) F294 or L294 and D275. Error bars represent mean ± standard deviation (SD).

interactions, and its neighboring glycans, N270 and N590, with differences across the variants (Figures S14 and S15). Taken together, we posit that the A835D mutation introduces dense packing of negative charge in the area immediately surrounding the FPPR and SD1/base of the RBD. To alleviate this negative charge, A835D and DM spikes recruit R830 to this region for better charge balance. We believe the F294L mutation contributes to a loosening of interactions at the base of the NTD, particularly at the base of a loop known as the N2R linker, which is responsible for passing allosteric information between the NTD and RBD. We believe the DM spike is therefore marginally more adept at alleviating negative charge density via opening its RBD, thus contributing to the slight

increase in proportion of DM RBDs initiating transition to the up state.

620-loop exhibited enhanced dynamics in the DM system

Dynamics and positioning of the "630-loop" in SARS-CoV-2 spike (residues 620–640) have been shown to correlate significantly with RBD up/down conformational states.[87] Moreover, perturbations in or near this loop, including the D614G mutation, to alter its flexibility/compactness have been shown to directly impact RBD conformations.[28,88] Based on these insights from the SARS-CoV-2 spike, we hypothesized that the structurally analogous region in the SHC014 spike, which we have called the "620-loop," may similarly modulate allosteric communication between the NTD, RBD, and FPPR. Per-residue flexibilities for the 620-loop (Figures 5A and 5B) as a function of spike variant indicate that while all spike 620-loops exhibit significant degrees of flexibility relative to other spike domains, the F294L variant demonstrates remarkable variance in flexibility in this region, Figure 5C.

To further characterize the behavior of the 620-loop in SHC014, we tracked how much the 620-loop deviates from the center of the SHC014 spike protein core, termed the 620-loop-core distance (Video S5). The 620-loop-core distances for all SHC014 variants occupy a similar right-skewed probability distribution. However, DM spike appears to sample larger 620-loop-core distances more frequently than the other spikes (Figures S16A and S16B), which may correlate with sampling of an intermediate RBD opening conformation observed for this variant. The Y623H mutation, positioned within the 620-loop of SD2 in SHC014 GP, has previously been shown to enhance viral entry, potentially driving the opening of the RBD.[89] While preparing this manuscript, Tse et al.[90] reported that the Y623H substitution may modulate the propensity of SHC014 RBD to sample an open conformation, thereby increasing viral infectivity by enhancing spike:ACE2 binding. Altogether, our work complements these findings by uncovering the varying degrees of flexibility in the 620-loop across the variants, which may allosterically alter RBD conformations (Figures S16C and S16D).

A835D mutation reshapes long-range allosteric communication

To understand how the F294L and the A835D mutations reshape allostery within the SHC014 spike, particularly in altering domain-domain communication critical for RBD opening, we carried out dynamical network analysis using the WISP method.[76] Using a weighted residue network,

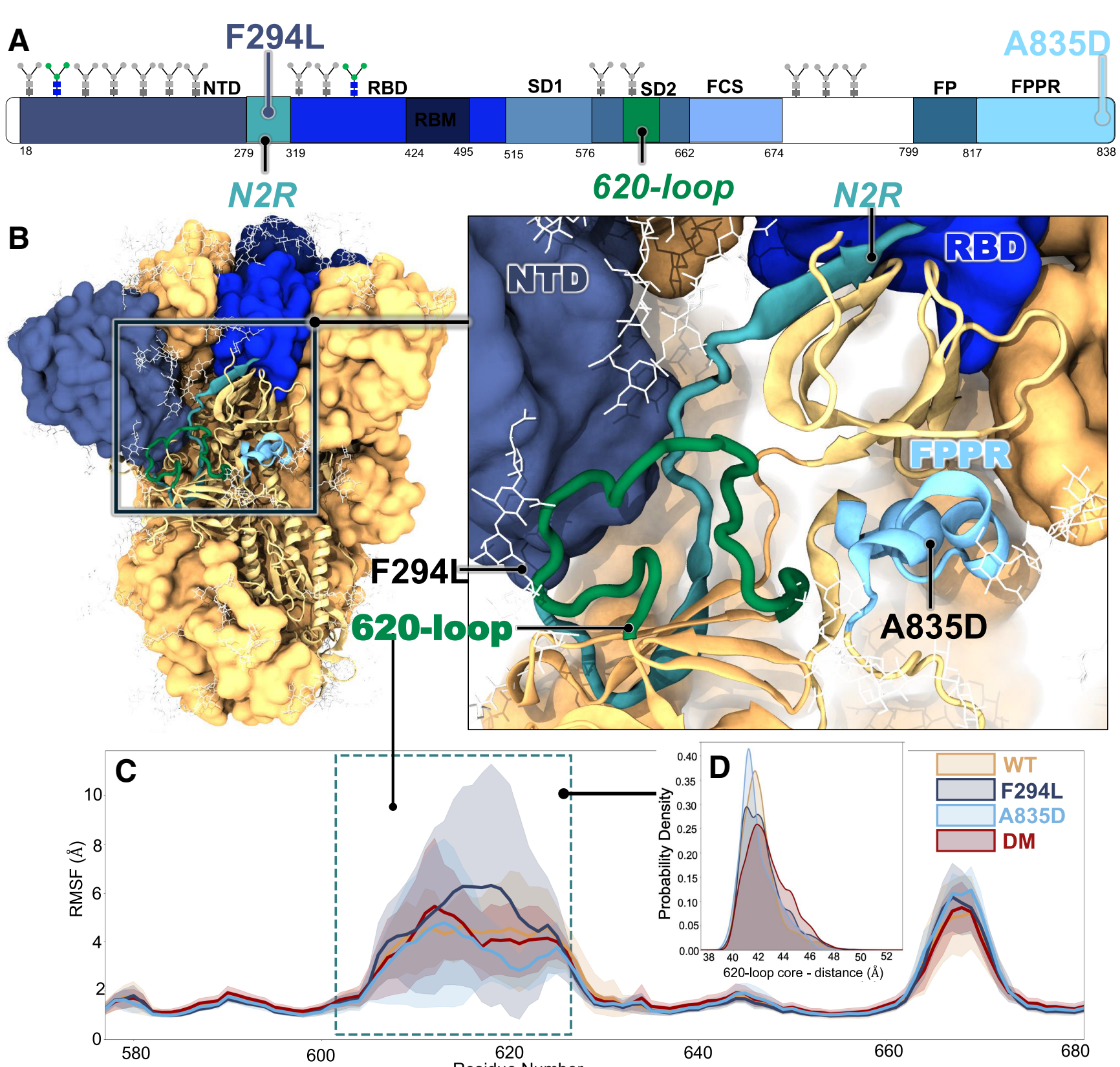

Figure 5. The 620-loop exhibited varying degrees of flexibility. (A) One-dimensional sequence of SHC014 spike protein from the NTD to the FPPR region only. The 620-loop is shown in green. (B) Close view of the 620-loop and the N2R linker. Position of the F294L mutation is shown as a dark blue sphere, while the A835D position is shown as a cyan sphere. (C) RMSF plot depicting per-residue flexibility within the SD2 domain with a particular focus on the 620-loop. (D) 620-loop-core distance plot calculated based on the center of mass of 620-loop to the central helices of the spike core.

we represented each residue as a node and connected pairs of residues (edges) with weights proportional to the strength of their correlated motions. This graph-based method not only uncovers how a group of nodes that form a domain can establish networks with distant regions within the spike but also how the two-point mutations studied herein can influence patterns in allosteric communications and structural rearrangements (Figures 6A and 6B). From our dynamical network analyses, we extracted edge-weighted domain-domain pairs (Figure S17) averaged over all three chains of the spike trimer to map a continuous allosteric pathway, and we noted differences in communication routes across the variants, including primary (Figures 6C–6F) and nonprimary (Figure S18) allosteric pathways. Interestingly, SD1, positioned at the base of the RBD (Figure 6B), serves as a critical allosteric hub where nearly all identified communication pathways converge, thereby prompting it to act like a "molecular junction" that relays information between functionally distinct regions of the spike. Just like a traffic junction that allows multiple roads to intersect, SD1 relays information from either the NTD or the SD2 to the RBD. Previous structural studies of the full-length SARS-CoV-2 spike suggested SD1 to be a structural relay for communication between RBD and FPPR, capable of sensing displacement on either side.[27] Similarly, computational modeling of the spike protein using coarse-grained simulations has reported SD1 as an allosteric center between RBD and FPPR.[91] Therefore, our findings corroborate the existing body of evidence that SD1 acts as an important allosteric region.

The R2N linker, which connects the RBD to the base of the NTD and SD2, has been shown to primarily facilitate allosteric communication in the Delta and Omicron SARS-CoV-2 spike variants.[86] Notably, both A835D and DM displayed a direct allosteric pathway from the FPPR via the SD2 to the R2N linker, then to the SD1, which acts as the allosteric bridge, ultimately relaying the information to the RBD (Figures 6E and 6F). The SD2 contains both the FCS and the 620-loop; therefore, to understand the role of the 620-loop in the allosteric networks, we selected both the 620-loop alone and the SD2 with the FCS, hereafter referred to as SD2'. We identified three main communication pathways to the RBD, named "pathway 1," "pathway 2," and "pathway 3." In the WT and F294L variant, pathway 1 shows baseline allosteric communication from the 620-loop to the RBD (Figures 6C and 6D). Notably, in the A835D and DM variants, pathway 1 starts at the FPPR, then proceeds to the 620-loop, and ultimately to the RBD,

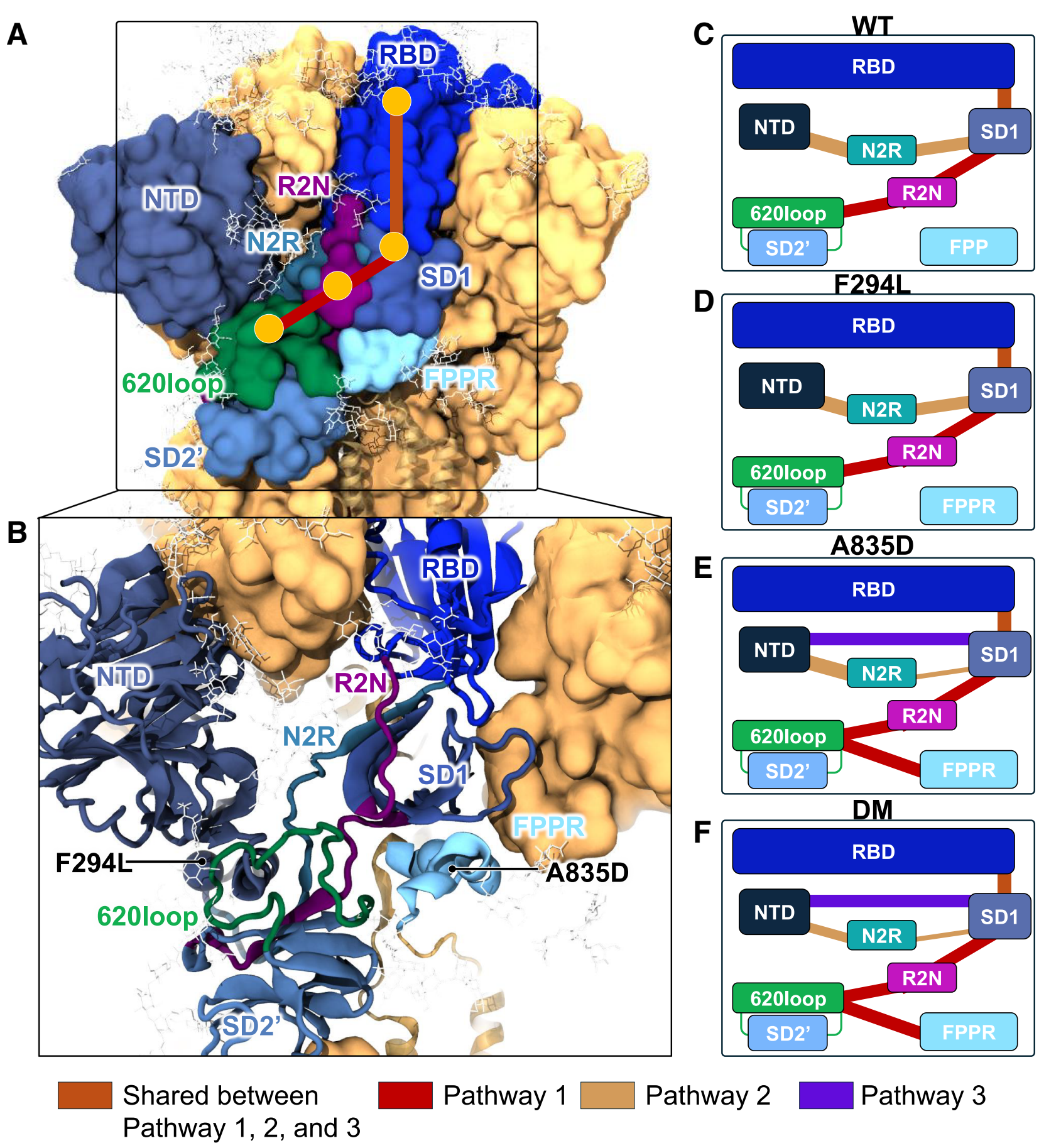

Figure 6. Dynamical network analysis reveals communication pathways across the SHC014 spike variants. (A) Surface representation of the SHC014 spike and domains within the allosteric routes. The path connecting the 620-loop to the RBD is depicted in gold spheres. (B) A cartoon representation and close view of domains participating in the primary allosteric communication pathways. (C–F) A residue-based graph network depicting how domains communicate with each other, weighted by their edges for WT, F294L, A835D, and DM, respectively. Each node represents a domain, and edge thickness corresponds to communication strength. Brown edges highlight the shared route between all three pathways; red represents pathway 1 in WT and F294L variants in the following order: 620-loop-to-SD2'-to-R2N-to-SD1-to-RBD, as well as A835D and DM, which extend the communication in the following order: FPPR-to-620-loop-to-SD2'-to-R2N-to-SD1-to-RBD among the variants. Light orange represents allosteric communication along pathway 2 in the following order: NTD-to-N2R-to-SD1-to-RBD, while purple depicts pathway 3 seen only in A835D and DM: NTD-to-SD1-to-RBD. Other nonprimary domain-domain communication pairs have been removed to improve visualization. SD2′ denotes the SD2 domain without the 620-loop.

indicating how the D835 mutation extends the baseline pathway, enabling FPPR to contribute to allosteric communication with the RBD (Figures 6E and 6F). Similarly, the D614G mutation, located far from the RBD, has been reported to reshape allosteric networks in the SARS-CoV-2 spike.[86] Moreover, the FPPR-to-620-loop-to-RBD crosstalk observed in A835D's and DM's pathway 1 is consistent with earlier findings (Figures 5D, S16A, and S16B) showing enhanced flexibility in the DM 620-loop compared to other variants. These results delineate how point mutations distant from the RBD, such as A835D with the FPPR, can modulate both structural dynamics and long-range allosteric communication in the spike.

Furthermore, the N2R linker has been proposed to facilitate communication in the SARS-CoV-2 spike S1, leading to RBD opening.[78,92] In all SHC014 spike variants considered here, we also identified a conserved communication route, hereafter referred to as pathway 2, running from NTD-to-N2R-to-SD1-to-RBD-to-RBM (Figures 6C–6F). Moreover, given increased edge weights for SD1-to-N2R nodes, pathway 2 is more prominent in WT and F294L than in other variants, indicating that information flowing from the NTD to the SD1 has to first pass through the N2R linker. However, we observed that in A835D and DM, a "shortcut" is established, hereafter referred to as pathway 3 (Figures 6E and 6F), where information can flow directly from the NTD to the SD1, which rebalances the communication within the three edges instead of the two in WT and F294L. Kearns et al.[86] reported distinct allosteric communication across human SARS-CoV-2 spike variants, with Delta and ancestral spikes primarily transmitting information between the NTD and RBD via the R2N and N2R linkers, respectively, whereas Omicron was capable of utilizing both. Similarly, SHC014 DM and A835D variants may be capable of using pathways 1, 2, and 3 to potentially enhance RBD opening dynamics and increase ACE2 binding efficiency, as we have seen in previous experimental work.[51]

Altogether, our multiple μs simulations reveal a mechanistic basis of how two point mutations (F294L and A835D) alter the structural dynamics and reshape allosteric communication of the SHC014 spike protein. We therefore hypothesize that mutations that strengthen inter-protomer interactions, and alter allosteric pathways, may stabilize the spike RBD in one-up state, a receptor-accessible conformation associated with spike activation and potentially lead to higher virulence, as observed in the SARS-CoV-2 D614G mutation.[47,50,81,86] Key functional outcomes of the F294L and A835D mutations in SHC014, including receptor binding, were experimentally characterized in our previous study[51] using enzyme-linked immunosorbent assay (ELISA) and biolayer interferometry (BLI), while cellular entry was determined using vesicular stomatitis virus (VSV)-based pseudovirus infection assays. The mechanistic relationships identified herein could be further evaluated using tissue or animal models to assess their impact on viral fitness and pathogenesis. In pre-zoonotic sarbecoviruses such as SHC014, these insights may help explain how mutations modulate spike activation and infectivity prior to zoonotic emergence, although whether these mechanisms generalize to SARS-CoV-2 variants remains to be determined.

CONCLUSION

In this work, we present multiple 1-μs-long explicitly solvated all-atom MD simulations of the fully glycosylated SHC014-CoV WT and variant spike ectodomains. Our previous work demonstrated that the acquisition of two novel mutations, F294L and A835D, in the NTD and FPPR, respectively, enhances viral infectivity by increasing RBD availability to the host cell receptor, ACE2.[51] Herein, we have observed that these mutations lead to the structural rearrangement of, and altered communication between, domains affecting the global motion of the spike protein. The WT RBM samples multiple conformations, which may increase susceptibility to premature S1 shedding. In contrast, the mutants, particularly the DM, exhibit narrower and unimodal RBM trimeric area distributions that reflect greater preorganization and structural compactness. This difference may contribute to the lower infectivity of WT relative to the mutants, consistent with our previous experimental ranking of SHC014 infectivity (DM > A835D > F294L > WT).[51] The structures of SL bat CoVs, including SHC014, have been resolved with the RBD in the down conformation.[51,89] Notably, we also observed that the DM RBD initiates increased propensity of opening even in conventional MD simulations, which is unusual given the timescales of spike opening (milliseconds to seconds), which typically require enhanced sampling methods to observe.[30,85,86,93–97] While the F294L and A835D mutations enhance ACE2 binding and cell entry, they also expose the SHC014-CoV spike to neutralizing antibodies such as ADG2, indicating a fitness trade-off and potential therapeutic avenue that may be exploited for the design of effective countermeasures to support pandemic preparedness efforts.

We observed that initiation of the RBD opening event in the DM may be allosterically modulated by the increased flexibility of the 620-loop and its crosstalk. Consistent with our findings, previous studies have shown that the Y623H mutation[89,98] within the 620-loop of SHC014-CoV led to an increase in viral infectivity compared to the WT, further affirming the role of this loop in RBD openness to enhance cell entry. The 620-loop in SHC014-CoV corresponds to the 630-loop in SARS-CoV-2 spike, which has similarly been shown to correlate with RBD motion.[86] At the atomic scale, the F294L mutation appears to disrupt the network of π-mediated interactions, thereby enhancing the flexibility of the N2R linker and contributing to marked accessibility of RBD to ACE2. Conversely, we found that the DM alleviates the dense negative packing around the FPPR region introduced by the A835D mutation to favor the RBD transitioning to the open state. Through dynamical network

analyses, we revealed three allosteric pathways via the R2N and N2R linkers, as well as the NTD, all converging at the SD1 domain, which acts as an allosteric hub that relays information to the RBD. While pathway 1 was present across all variants, it extended to include the FPPR region in the A835D and DM variants, suggesting that this pathway is strengthened in these mutants compared to WT and F294L. This finding illustrates how distant mutations, such as A835D in the FPPR, can reshape long-range allosteric communication within the spike. In contrast, pathway 2 requires information to pass through the N2R linker from the NTD to SD1. Although pathway 2 was observed in A835D and DM, it exhibited reduced communication compared to WT and F294L. Interestingly, a new route, termed pathway 3, emerged in A835D and DM, establishing a direct shortcut from the NTD to SD1 that may balance the communication in pathway 2. Altogether, our work provides atomistic insights into the structural dynamics and molecular adaptations governing WT and mutant SHC014-CoVs and probable therapeutic opportunities against SHC014 and related SL bat CoVs for pandemic preparedness efforts.

DATA AVAILABILITY

All simulation trajectories, simulation scripts, analysis scripts, and data files will be made accessible for download on the Amaro Lab website at https://amarolab.ucsd.edu/data.php.

ACKNOWLEDGMENTS

We are grateful for the Leadership Resource Allocation (LRAC-CHE23002) for the use of the Frontera Supercomputer provided by the Texas Advanced Computing Centre (TACC). Additional computing resources were provided by the Triton Shared Computing Cluster (TSCC) at the San Diego Supercomputer Centre. T.A.B. is supported by the Shurl and Kay Curci Foundation (https://curcifoundation.org/), the A.G. Leventis Foundation (https://www.leventisfoundation.org/), and Thermo Fisher Scientific Antibody Fellowships (https://www.thermofisher.com/us/en/home/life-science/antibodies/thermo-fisher-scientific-antibody-scholarship-program.html). Furthermore, this work was supported by National Institutes of Health (NIH; https://www.niaid.nih.gov/) grants R01AI132633 and K08AI180364 to K.C. and E.H.M., respectively. E.H.M. was additionally supported by the Institute for Clinical and Translational Research at Einstein and Montefiore (https://einsteinmed.edu/centers/ictr) (K12TR004411). A.L.T. was additionally supported by the NIH training grants T32GM149364 (Medical Scientist Training Program) at Albert Einstein College of Medicine. This work was funded in part by Welch Foundation grant number F-0003-19620604 (J.S.M.).

AUTHOR CONTRIBUTIONS

T.A.B. and F.L.K. designed and conducted all-atom conventional MD simulations. T.A.B., F.L.K., C.C.-T., and L.C. performed all computational analyses. A.L.T., C.M.A., and G.L. performed all viral assays and cryo-EM experimentation in a previous study,[51] which served as the basis of this work by identifying the F294L and A835D mutations and also resolved cryo-EM SHC014 WT spike structure (PDB: 9CAS) used herein. E.H.M., K.C., and J.S.M. guided all previous experiments,[51] and R.E.A. oversaw all simulations and analyses. T.A.B., F.L.K., and R.E.A. wrote the article, with contributions from all authors.

DECLARATION OF INTERESTS

K.C. is a member of the scientific advisory board and holds shares in Integrum Scientific, LLC. K.C. is a cofounder of and holds shares in Eitr Biologics, Inc.

SUPPLEMENTAL INFORMATION

Supplemental information can be found online at https://doi.org/10.1016/j.bpj.2026.04.023.

REFERENCES

1. Li, W., Z. Shi, …, L. F. Wang. 2005. Bats Are Natural Reservoirs of SARS-Like Coronaviruses. *Science.* 310:676–679. https://doi.org/10.1126/science.1118391.
2. Ruiz-Aravena, M., C. McKee, …, R. K. Plowright. 2022. Ecology, evolution and spillover of coronaviruses from bats. *Nat. Rev. Microbiol.* 20:299–314. https://doi.org/10.1038/s41579-021-00652-2.
3. Cui, J., F. Li, and Z. L. Shi. 2019. Origin and evolution of pathogenic coronaviruses. *Nat. Rev. Microbiol.* 17:181–192. https://doi.org/10.1038/s41579-018-0118-9.
4. Zhou, H., J. Ji, …, W. Shi. 2021. Identification of novel bat coronaviruses sheds light on the evolutionary origins of SARS-CoV-2 and related viruses. *Cell.* 184:4380–4391.e14. https://doi.org/10.1016/j.cell.2021.06.008.
5. Cohen, L. E., A. C. Fagre, …, D. J. Becker. 2023. Coronavirus sampling and surveillance in bats from 1996–2019: a systematic review and meta-analysis. *Nat. Microbiol.* 8:1176–1186. https://doi.org/10.1038/s41564-023-01375-1.
6. Temmam, S., K. Vongphayloth, …, M. Eloit. 2022. Bat coronaviruses related to SARS-CoV-2 and infectious for human cells. *Nature.* 604:330–336. https://doi.org/10.1038/s41586-022-04532-4.
7. Ge, X. Y., J. L. Li, …, Z. L. Shi. 2013. Isolation and characterization of a bat SARS-like coronavirus that uses the ACE2 receptor. *Nature.* 503:535–538. https://doi.org/10.1038/nature12711.
8. Letko, M., A. Marzi, and V. Munster. 2020. Functional assessment of cell entry and receptor usage for SARS-CoV-2 and other lineage B betacoronaviruses. *Nat. Microbiol.* 5:562–569. https://doi.org/10.1038/s41564-020-0688-y.
9. Hu, B., L. P. Zeng, …, Z. L. Shi. 2017. Discovery of a rich gene pool of bat SARS-related coronaviruses provides new insights into the origin of SARS coronavirus. *PLoS Pathog.* 13:e1006698. Drosten C. https://doi.org/10.1371/journal.ppat.1006698.
10. Ren, W., X. Qu, …, Z. Shi. 2008. Difference in Receptor Usage between Severe Acute Respiratory Syndrome (SARS) Coronavirus and SARS-Like Coronavirus of Bat Origin. *J. Virol.* 82:1899–1907. https://doi.org/10.1128/JVI.01085-07.
11. Liu, K., X. Pan, …, Q. Wang. 2021. Binding and molecular basis of the bat coronavirus RaTG13 virus to ACE2 in humans and other species. *Cell.* 184:3438–3451.e10. https://doi.org/10.1016/j.cell.2021.05.031.
12. Sheahan, T., B. Rockx, …, R. Baric. 2008. Mechanisms of Zoonotic Severe Acute Respiratory Syndrome Coronavirus Host Range Expansion in Human Airway Epithelium. *J. Virol.* 82:2274–2285. https://doi.org/10.1128/JVI.02041-07.
13. Menachery, V. D., B. L. Yount, …, R. S. Baric. 2015. A SARS-like cluster of circulating bat coronaviruses shows potential for human emergence. *Nat. Med.* 21:1508–1513. https://doi.org/10.1038/nm.3985.
14. Bosch, B. J., R. Van Der Zee, …, P. J. M. Rottier. 2003. The Coronavirus Spike Protein Is a Class I Virus Fusion Protein: Structural and Functional Characterization of the Fusion Core Complex. *J. Virol.* 77:8801–8811. https://doi.org/10.1128/JVI.77.16.8801-8811.2003.
15. Huang, Y., C. Yang, …, S. W. Liu. 2020. Structural and functional properties of SARS-CoV-2 spike protein: potential antivirus drug

development for COVID-19. *Acta Pharmacol. Sin.* 41:1141–1149. https://doi.org/10.1038/s41401-020-0485-4.

16. Peacock, T. P., D. H. Goldhill, …, W. S. Barclay. 2021. The furin cleavage site in the SARS-CoV-2 spike protein is required for transmission in ferrets. *Nat. Microbiol.* 6:899–909. https://doi.org/10.1038/s41564-021-00908-w.
17. Wrapp, D., N. Wang, …, J. S. McLellan. 2020. Cryo-EM structure of the 2019-nCoV spike in the prefusion conformation. *Science.* 367:1260–1263. https://doi.org/10.1126/science.abb2507.
18. Vankadari, N. 2020. Structure of Furin Protease Binding to SARS-CoV-2 Spike Glycoprotein and Implications for Potential Targets and Virulence. *J. Phys. Chem. Lett.* 11:6655–6663. https://doi.org/10.1021/acs.jpclett.0c01698.
19. Papa, G., D. L. Mallery, …, L. C. James. 2021. Furin cleavage of SARS-CoV-2 Spike promotes but is not essential for infection and cell-cell fusion. *PLoS Pathog.* 17:e1009246. Lee B. https://doi.org/10.1371/journal.ppat.1009246.
20. Bestle, D., M. R. Heindl, …, E. Böttcher-Friebertshäuser. 2020. TMPRSS2 and furin are both essential for proteolytic activation of SARS-CoV-2 in human airway cells. *Life Sci. Alliance.* 3:e202000786. https://doi.org/10.26508/lsa.202000786.
21. Essalmani, R., J. Jain, …, E. Decroly. 2022. Distinctive Roles of Furin and TMPRSS2 in SARS-CoV-2 Infectivity. *J. Virol.* 96:e00128. Heise MT. https://doi.org/10.1128/jvi.00128-22.
22. Wang, Q., Y. Zhang, …, J. Qi. 2020. Functional Basis of SARS-CoV-2 Entry by Using Human ACE2. *Cell.* 181:894–904.e9. https://doi.org/10.1016/j.cell.2020.03.045.
23. Jackson, C. B., M. Farzan, …, H. Choe. 2022. Mechanisms of SARS-CoV-2 entry into cells. *Nat. Rev. Mol. Cell Biol.* 23:3–20. https://doi.org/10.1038/s41580-021-00418-x.
24. Tortorici, M. A., A. C. Walls, …, D. Veesler. 2019. Structural basis for human coronavirus attachment to sialic acid receptors. *Nat. Struct. Mol. Biol.* 26:481–489. https://doi.org/10.1038/s41594-019-0233-y.
25. Watanabe, Y., J. D. Allen, …, M. Crispin. 2020. Site-specific glycan analysis of the SARS-CoV-2 spike. *Science.* 369:330–333. https://doi.org/10.1126/science.abb9983.
26. Walls, A. C., Y. J. Park, …, D. Veesler. 2020. Structure, Function, and Antigenicity of the SARS-CoV-2 Spike Glycoprotein. *Cell.* 181:281–292.e6. https://doi.org/10.1016/j.cell.2020.02.058.
27. Cai, Y., J. Zhang, …, B. Chen. 2020. Distinct conformational states of SARS-CoV-2 spike protein. *Science.* 369:1586–1592. https://doi.org/10.1126/science.abd4251.
28. Zhang, J., Y. Cai, …, B. Chen. 2021. Structural impact on SARS-CoV-2 spike protein by D614G substitution. *Science.* 372:525–530. https://doi.org/10.1126/science.abf2303.
29. Moreira, R. A., H. V. Guzman, …, A. B. Poma. 2020. Characterization of Structural and Energetic Differences between Conformations of the SARS-CoV-2 Spike Protein. *Materials.* 13:5362. https://doi.org/10.3390/ma13235362.
30. Sztain, T., S. H. Ahn, …, R. E. Amaro. 2021. A glycan gate controls opening of the SARS-CoV-2 spike protein. *Nat. Chem.* 13:963–968. https://doi.org/10.1038/s41557-021-00758-3.
31. Casalino, L., Z. Gaieb, …, R. E. Amaro. 2020. Beyond Shielding: The Roles of Glycans in the SARS-CoV-2 Spike Protein. *ACS Cent. Sci.* 6:1722–1734. https://doi.org/10.1021/acscentsci.0c01056.
32. Barros, E. P., L. Casalino, …, R. E. Amaro. 2021. The flexibility of ACE2 in the context of SARS-CoV-2 infection. *Biophys. J.* 120:1072–1084. https://doi.org/10.1016/j.bpj.2020.10.036.
33. V'kovski, P., A. Kratzel, …, V. Thiel. 2021. Coronavirus biology and replication: implications for SARS-CoV-2. *Nat. Rev. Microbiol.* 19:155–170. https://doi.org/10.1038/s41579-020-00468-6.
34. Li, F. 2016. Structure, Function, and Evolution of Coronavirus Spike Proteins. *Annu. Rev. Virol.* 3:237–261. https://doi.org/10.1146/annurev-virology-110615-042301.
35. Watanabe, Y., T. A. Bowden, …, M. Crispin. 2019. Exploitation of glycosylation in enveloped virus pathobiology. *Biochim. Biophys. Acta. Gen. Subj.* 1863:1480–1497. https://doi.org/10.1016/j.bbagen.2019.05.012.
36. Raman, R., K. Tharakaraman, …, R. Sasisekharan. 2016. Glycan–protein interactions in viral pathogenesis. *Curr. Opin. Struct. Biol.* 40:153–162. https://doi.org/10.1016/j.sbi.2016.10.003.
37. Shajahan, A., N. T. Supekar, …, P. Azadi. 2020. Deducing the N- and O-glycosylation profile of the spike protein of novel coronavirus SARS-CoV-2. *Glycobiology.* 30:981–988. https://doi.org/10.1093/glycob/cwaa042.
38. Pasala, C., S. Sharma, …, G. Chiosis. 2024. N-Glycosylation as a Modulator of Protein Conformation and Assembly in Disease. *Biomolecules.* 14:282. https://doi.org/10.3390/biom14030282.
39. Barton, M. I., S. A. MacGowan, …, P. A. Van Der Merwe. 2021. Effects of common mutations in the SARS-CoV-2 Spike RBD and its ligand, the human ACE2 receptor on binding affinity and kinetics. *eLife.* 10:e70658. https://doi.org/10.7554/eLife.70658.
40. Liu, Y., J. Liu, …, S. C. Weaver. 2022. The N501Y spike substitution enhances SARS-CoV-2 infection and transmission. *Nature.* 602:294–299. https://doi.org/10.1038/s41586-021-04245-0.
41. Ou, X., G. Xu, …, Z. Qian. 2023. Host susceptibility and structural and immunological insight of S proteins of two SARS-CoV-2 closely related bat coronaviruses. *Cell Discov.* 9:78. https://doi.org/10.1038/s41421-023-00581-9.
42. Kang, L., G. He, …, J. Weger-Lucarelli. 2021. A selective sweep in the Spike gene has driven SARS-CoV-2 human adaptation. *Cell.* 184:4392–4400.e4. https://doi.org/10.1016/j.cell.2021.07.007.
43. Zhang, S., Q. Liang, …, X. Wang. 2022. Loss of Spike N370 glycosylation as an important evolutionary event for the enhanced infectivity of SARS-CoV-2. *Cell Res.* 32:315–318. https://doi.org/10.1038/s41422-021-00600-y.
44. Koehler, M., A. Ray, …, D. Alsteens. 2021. Molecular insights into receptor binding energetics and neutralization of SARS-CoV-2 variants. *Nat. Commun.* 12:6977. https://doi.org/10.1038/s41467-021-27325-1.
45. Korber, B., W. M. Fischer, …, M. D. Wyles. 2020. Tracking Changes in SARS-CoV-2 Spike: Evidence that D614G Increases Infectivity of the COVID-19 Virus. *Cell.* 182:812–827.e19. https://doi.org/10.1016/j.cell.2020.06.043.
46. Plante, J. A., Y. Liu, …, P. Y. Shi. 2021. Spike mutation D614G alters SARS-CoV-2 fitness. *Nature.* 592:116–121. https://doi.org/10.1038/s41586-020-2895-3.
47. Yurkovetskiy, L., X. Wang, …, J. Luban. 2020. Functional Analysis of the D614G SARS-CoV-2 Spike Protein Variant. *Cell.* 183:739–751.e8. https://doi.org/10.1016/j.cell.2020.09.032.
48. Zhang, L., C. B. Jackson, …, H. Choe. 2020. SARS-CoV-2 spike-protein D614G mutation increases virion spike density and infectivity. *Nat. Commun.* 11:6013. https://doi.org/10.1038/s41467-020-19808-4.
49. Daniloski, Z., T. X. Jordan, …, N. E. Sanjana. 2021. The Spike D614G mutation increases SARS-CoV-2 infection of multiple human cell types. *eLife.* 10:e65365. https://doi.org/10.7554/eLife.65365.
50. Ozono, S., Y. Zhang, …, K. Tokunaga. 2021. SARS-CoV-2 D614G spike mutation increases entry efficiency with enhanced ACE2-binding affinity. *Nat. Commun.* 12:848. https://doi.org/10.1038/s41467-021-21118-2.
51. Tse, A. L., C. M. Acreman, …, E. H. Miller. 2024. Distinct pathways for evolution of enhanced receptor binding and cell entry in SARS-like bat coronaviruses. *PLoS Pathog.* 20:e1012704. Bloom JD. https://doi.org/10.1371/journal.ppat.1012704.
52. Schwede, T., J. Kopp, …, M. C. Peitsch. 2003. SWISS-MODEL: an automated protein homology-modeling server. *Nucleic Acids Res.* 31:3381–3385. https://doi.org/10.1093/nar/gkg520.
53. Jumper, J., R. Evans, …, D. Hassabis. 2021. Highly accurate protein structure prediction with AlphaFold. *Nature.* 596:583–589. https://doi.org/10.1038/s41586-021-03819-2.
54. Jacobson, M. P., D. L. Pincus, …, R. A. Friesner. 2004. A hierarchical approach to all-atom protein loop prediction. *Proteins.* 55:351–367. https://doi.org/10.1002/prot.10613.

55. Šali, A., and T. L. Blundell. 1993. Comparative Protein Modelling by Satisfaction of Spatial Restraints. *J. Mol. Biol.* 234:779–815. https://doi.org/10.1006/jmbi.1993.1626.

56. Bangaru, S., G. Ozorowski, …, A. B. Ward. 2020. Structural analysis of full-length SARS-CoV-2 spike protein from an advanced vaccine candidate. *Science.* 370:1089–1094. https://doi.org/10.1126/science.abe1502.

57. Gupta, R., and S. Brunak. 2002. Prediction of glycosylation across the human proteome and the correlation to protein function. *Pac. Symp. Biocomput.* 7:310–322.

58. Zhang, M., B. Gaschen, …, B. Korber. 2004. Tracking global patterns of N-linked glycosylation site variation in highly variable viral glycoproteins: HIV, SIV, and HCV envelopes and influenza hemagglutinin. *Glycobiology.* 14:1229–1246. https://doi.org/10.1093/glycob/cwh106.

59. Allen, J. D., D. P. Ivory, …, M. Crispin. 2023. The diversity of the glycan shield of sarbecoviruses related to SARS-CoV-2. *Cell Rep.* 42:112307. https://doi.org/10.1016/j.celrep.2023.112307.

60. Phillips, J. C., R. Braun, …, K. Schulten. 2005. Scalable molecular dynamics with NAMD. *J. Comput. Chem.* 26:1781–1802. https://doi.org/10.1002/jcc.20289.

61. Olsson, M. H. M., C. R. Søndergaard, …, J. H. Jensen. 2011. PROPKA3: Consistent Treatment of Internal and Surface Residues in Empirical pKa Predictions. *J. Chem. Theory Comput.* 7:525–537. https://doi.org/10.1021/ct100578z.

62. Sastry, G. M., M. Adzhigirey, …, W. Sherman. 2013. Protein and ligand preparation: parameters, protocols, and influence on virtual screening enrichments. *J. Comput. Aided Mol. Des.* 27:221–234. https://doi.org/10.1007/s10822-013-9644-8.

63. Guvench, O., E. R. Hatcher, …, A. D. MacKerell. 2009. CHARMM Additive All-Atom Force Field for Glycosidic Linkages between Hexopyranoses. *J. Chem. Theory Comput.* 5:2353–2370. https://doi.org/10.1021/ct900242e.

64. Huang, J., and A. D. MacKerell. 2013. CHARMM36 all-atom additive protein force field: Validation based on comparison to NMR data. *J. Comput. Chem.* 34:2135–2145. https://doi.org/10.1002/jcc.23354.

65. Humphrey, W., A. Dalke, and K. Schulten. 1996. VMD: visual molecular dynamics. *J. Mol. Graph.* 14:33–38. https://doi.org/10.1016/0263-7855(96)00018-5.

66. Jorgensen, W. L., J. Chandrasekhar, …, M. L. Klein. 1983. Comparison of simple potential functions for simulating liquid water. *J. Chem. Phys.* 79:926–935. https://doi.org/10.1063/1.445869.

67. Phillips, J. C., D. J. Hardy, …, E. Tajkhorshid. 2020. Scalable molecular dynamics on CPU and GPU architectures with NAMD. *J. Chem. Phys.* 153:044130. https://doi.org/10.1063/5.0014475.

68. Turq, P., F. Lantelme, and H. L. Friedman. 1977. Brownian dynamics: Its application to ionic solutions. *J. Chem. Phys.* 66:3039–3044. https://doi.org/10.1063/1.434317.

69. Feller, S. E., Y. Zhang, …, B. R. Brooks. 1995. Constant pressure molecular dynamics simulation: The Langevin piston method. *J. Chem. Phys.* 103:4613–4621. https://doi.org/10.1063/1.470648.

70. Darden, T., D. York, and L. Pedersen. 1993. Particle mesh Ewald: An $N \cdot \log(N)$ method for Ewald sums in large systems. *J. Chem. Phys.* 98:10089–10092. https://doi.org/10.1063/1.464397.

71. Ryckaert, J. P., G. Ciccotti, and H. J. C. Berendsen. 1977. Numerical integration of the cartesian equations of motion of a system with constraints: molecular dynamics of n-alkanes. *J. Comput. Phys.* 23:327–341. https://doi.org/10.1016/0021-9991(77)90098-5.

72. Michaud-Agrawal, N., E. J. Denning, …, O. Beckstein. 2011. MDAnalysis: a toolkit for the analysis of molecular dynamics simulations. *J. Comput. Chem.* 32:2319–2327. https://doi.org/10.1002/jcc.21787.

73. Gowers, R. J., M. Linke, …, O. Beckstein. 2016. MDAnalysis: A Python package for the rapid analysis of molecular dynamics simulations. *In* Benthall Rostrup Ed Proc 15th Python Sci Conf. SciPy. https://doi.org/10.25080/majora-629e541a-00e.

74. Case, D. A., H. M. Aktulga, …, K. M. Merz. 2023. AmberTools. *J. Chem. Inf. Model.* 63:6183–6191. https://doi.org/10.1021/acs.jcim.3c01153.

75. Case, D. A., H. M. Aktulga, …, P. A. Kollman. 2024. Amber 2024. *Univ Calif San Franc*. https://ambermd.org/index.php.

76. Van Wart, A. T., J. Durrant, …, R. E. Amaro. 2014. Weighted Implementation of Suboptimal Paths (WISP): An Optimized Algorithm and Tool for Dynamical Network Analysis. *J. Chem. Theory Comput.* 10:511–517. https://doi.org/10.1021/ct4008603.

77. Hagberg, Aric A., Daniel A. Schult, and J. Pieter. 2008. Swart., Exploring network structure, dynamics, and function using NetworkX. *In* Proceedings of the 7th Python in Science Conference (SciPy2008). G. Varoquaux, T. Vaught, and J. Millman, eds, pp. 11–15.

78. Gobeil, S. M. C., R. Henderson, …, P. Acharya. 2022. Structural diversity of the SARS-CoV-2 Omicron spike. *Mol. Cell.* 82:2050–2068.e6. https://doi.org/10.1016/j.molcel.2022.03.028.

79. Stalls, V., J. Lindenberger, …, P. Acharya. 2022. Cryo-EM structures of SARS-CoV-2 Omicron BA.2 spike. *Cell Rep.* 39:111009. https://doi.org/10.1016/j.celrep.2022.111009.

80. Hong, Q., W. Han, …, Y. Cong. 2022. Molecular basis of receptor binding and antibody neutralization of Omicron. *Nature.* 604:546–552. https://doi.org/10.1038/s41586-022-04581-9.

81. Gellenoncourt, S., N. Saunders, …, L. A. Chakrabarti. 2022. The Spike-Stabilizing D614G Mutation Interacts with S1/S2 Cleavage Site Mutations To Promote the Infectious Potential of SARS-CoV-2 Variants. *T. Gallagher, ed* 96:e0130122. https://doi.org/10.1128/jvi.01301-22.

82. Berger, I., and C. Schaffitzel. 2020. The SARS-CoV-2 spike protein: balancing stability and infectivity. *Cell Res.* 30:1059–1060. https://doi.org/10.1038/s41422-020-00430-4.

83. Piccoli, L., Y. J. Park, …, D. Veesler. 2020. Mapping Neutralizing and Immunodominant Sites on the SARS-CoV-2 Spike Receptor-Binding Domain by Structure-Guided High-Resolution Serology. *Cell.* 183:1024–1042.e21. https://doi.org/10.1016/j.cell.2020.09.037.

84. Shang, J., G. Ye, …, F. Li. 2020. Structural basis of receptor recognition by SARS-CoV-2. *Nature.* 581:221–224. https://doi.org/10.1038/s41586-020-2179-y.

85. Lu, M., P. D. Uchil, …, W. Mothes. 2020. Real-Time Conformational Dynamics of SARS-CoV-2 Spikes on Virus Particles. *Cell Host Microbe.* 28:880–891.e8. https://doi.org/10.1016/j.chom.2020.11.001.

86. Kearns, F. L., A. T. Bogetti, …, R. E. Amaro. 2025. D614G reshapes allosteric networks and opening mechanisms of SARS-CoV-2 spikes. *bioRxiv.* https://doi.org/10.1101/2025.03.07.642081.

87. Dokainish, H. M., and Y. Sugita. 2023. Structural effects of spike protein D614G mutation in SARS-CoV-2. *Biophys. J.* 122:2910–2920. https://doi.org/10.1016/j.bpj.2022.11.025.

88. Zhang, J., Y. Cai, …, B. Chen. 2022. Structural and functional impact by SARS-CoV-2 Omicron spike mutations. *Cell Rep.* 39:110729. https://doi.org/10.1016/j.celrep.2022.110729.

89. Qiao, S., and X. Wang. 2024. Structural determinants of spike infectivity in bat SARS-like coronaviruses RsSHC014 and WIV1. *J. Virol.* 98:e0034224. Gallagher T. https://doi.org/10.1128/jvi.00342-24.

90. Tse, A. L., G. Lasso, …, E. H. Miller. 2025. Bat sarbecovirus WIV1-CoV bears an adaptive mutation that alters spike dynamics and enhances ACE2 binding. *PLoS Pathog.* 21:e1013123. Ariën KK. https://doi.org/10.1371/journal.ppat.1013123.

91. Verkhivker, G. M. 2020. Molecular Simulations and Network Modeling Reveal an Allosteric Signaling in the SARS-CoV-2 Spike Proteins. *J. Proteome Res.* 19:4587–4608. https://doi.org/10.1021/acs.jproteome.0c00654.

92. Manrique, P. D., S. Chakraborty, …, S. Gnanakaran. 2023. Network analysis uncovers the communication structure of SARS-CoV-2 spike protein identifying sites for immunogen design. *iScience.* 26:105855. https://doi.org/10.1016/j.isci.2022.105855.

93. Zwier, M. C., J. L. Adelman, …, L. T. Chong. 2015. WESTPA: An Interoperable, Highly Scalable Software Package for Weighted Ensemble Simulation and Analysis. *J. Chem. Theory Comput.* 11:800–809. https://doi.org/10.1021/ct5010615.

94. Ovchinnikov, V., and M. Karplus. 2023. Free Energy Simulations of Receptor-Binding Domain Opening of the SARS-CoV-2 Spike Indicate a Barrierless Transition with Slow Conformational Motions. *J. Phys. Chem. B.* 127:8565–8575. https://doi.org/10.1021/acs.jpcb.3c05236.

95. Casalino, L., A. C. Dommer, …, R. E. Amaro. 2021. AI-driven multiscale simulations illuminate mechanisms of SARS-CoV-2 spike dynamics. *Int. J. High Perform. Comput. Appl.* 35:432–451. https://doi.org/10.1177/10943420211006452.

96. Laio, A., and F. L. Gervasio. 2008. Metadynamics: a method to simulate rare events and reconstruct the free energy in biophysics, chemistry and material science. *Rep. Prog. Phys.* 71:126601. https://doi.org/10.1088/0034-4885/71/12/126601.

97. Da Costa, C. H. S., C. A. B. De Freitas, …, M. S. Skaf. 2025. Binding Interactions of SARS-CoV-2 Spike Protein Variants: Omicron, XBB.1.9.2, and EG.5. *J. Chem. Inf. Model.* 65:7651–7667. https://doi.org/10.1021/acs.jcim.5c00308.

98. Tse, A. L., G. Lasso, …, E. H. Miller. 2025. Bat sarbecovirus WIV1-CoV bears an adaptive mutation that alters spike dynamics and enhances ACE2 binding. *PLoS Pathog.* 21:e1013123. https://doi.org/10.1101/2025.04.14.648681.

Biophysical Journal, Volume *125*

Supplemental information

Structural dynamics and allosteric communication of a SARS-like bat coronavirus spike glycoprotein

Toheeb A. Balogun, Fiona L. Kearns, Carla Calvó-Tusell, Alexandra L. Tse, Cory M. Acreman, Lorenzo Casalino, Gorka Lasso, Emily Happy Miller, Kartik Chandran, Jason S. McLellan, and Rommie E. Amaro

Supplementary Information for

Structural Dynamics and Allosteric Communication of a SARS-Like Bat Coronavirus Spike Glycoprotein

Toheeb A. Balogun[1], Fiona L. Kearns[1,2], Carla Calvó-Tusell[1], Alexandra L. Tse[3], Cory M. Acreman[4,5], Lorenzo Casalino[1], Gorka Lasso[3,6], Emily Happy Miller[3,7], Kartik Chandran[3], Jason S. McLellan[4], Rommie E. Amaro[1#]

1. Department of Molecular Biology, University of California, San Diego, La Jolla, California, United States of America

2. Current Address: Institute of Physical Chemistry, University of Freiburg, Albertstrasse 21, 79104 Freiburg, Germany

3. Department of Microbiology & Immunology, Albert Einstein College of Medicine, Bronx, New York, New York, United States of America

4. Department of Molecular Biosciences, The University of Texas at Austin, Austin, Texas, United States of America

5. Current Address: Division of Biology and Biological Engineering, California Institute of Technology, Pasadena, California, United States of America

6. Current Address: Division of Infection and Immunity, University College London, London, UK.

7. Department of Medicine, Albert Einstein College of Medicine, Bronx, New York, New York, United States of America

[#]Corresponding author: ramaro@ucsd.edu

Contents

Figure S1: Modeling of missing regions of SHC014 GP
Figure S2: Root mean square deviation (RMSD) of SHC014 variant simulations
Figure S3: Trimeric area calculation for NTD, RBD, RBM, SD2 and FPPR
Figure S4: RBD trimeric area
Figure S5: RBD-core distances
Figure S6: Accessible surface area of SHC014 spike domains
Figure S7: F294/L294-K266 contacts
Figure S8: F294/L294-K288 contacts
Figure S9: F294/L294-D275 contacts
Figure S10: A835/D835-R830 contacts
Figure S11: R830-D555 contacts
Figure S12: A835/D835-D555 contacts
Figure S13: Salt-bridge formation and A835-R830-D555 area

Figure S14: R830-N270 glycan contacts
Figure S15: R830-N590 glycan
Figure S16: Conformational flexibility of the 620-loop in SHC014 WT and mutants
Figure S17: Domain-domain communication
Figure S18: Allosteric communication pathways in SHC014 variants with non-primary pathways

Table S1: Complete details of SHC014 computational models and simulation information
Table S2: Summary of the full-length S protein all-atom MD simulations
Table S3. Glycan compositions for chains A, B, and C.
Table S4: Summary of average trimeric domain areas (Å²) for NTD, RBM, SD2, and FPPR in SHC014 WT and mutants.
Table S5: Statistical analysis of FPPR trimeric area differences across SHC014 spike variants

Movie S1: Contact among A835, D555, and R830.
Movie S2: Contact among D835, D555, and R830.
Movie S3: Contact among F294, K266, K288, and D275.
Movie S4: Contact among L294, K266, K288, and D275.
Movie S5: Dynamics of the DM 620-loop relative to the WT spike.

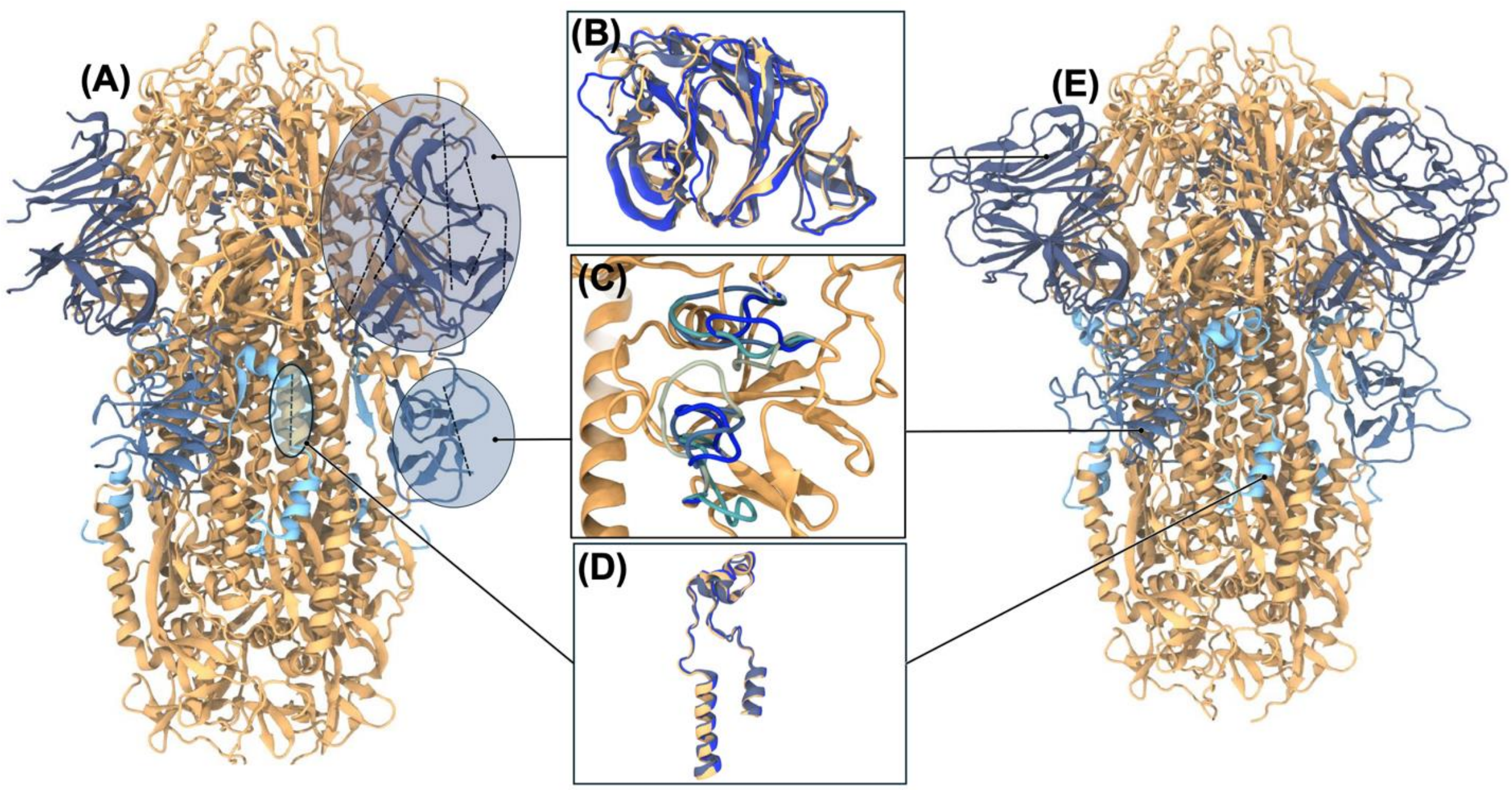

Figure S1: Modeling of missing regions of SHC014 GP (A) Wildtype cryoEM structure of SCH01with missing regions highlighted in broken lines with light filled sphere (B) NTD modeling using multiple protein structure prediction tools such as Alphafold (darkblue), Prime (blue) and SwissModel (Gold) (C) Modeling of missing loops in SD2 domain using ChimeraX (D) FPPR modeling using PDB 7JJI as template shown in blue, SwissModel (Gold) and Alphafold (darkblue) (E) SHC014 GP ectodomain with missing regions filled.

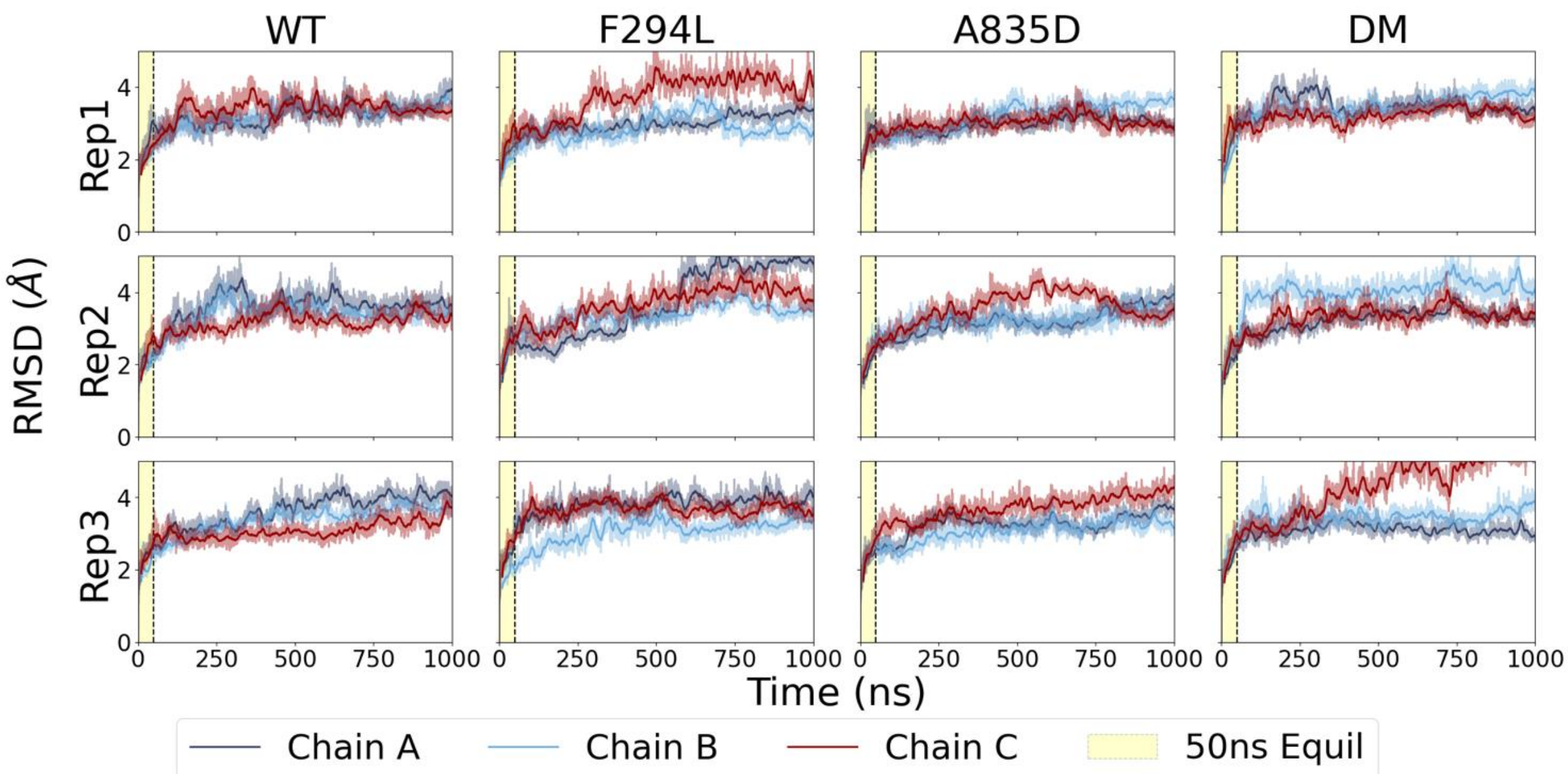

Figure S2: Root mean square deviation (RMSD) of SHC014 variants, the 50ns equilibration step before production run is shown in yellow.

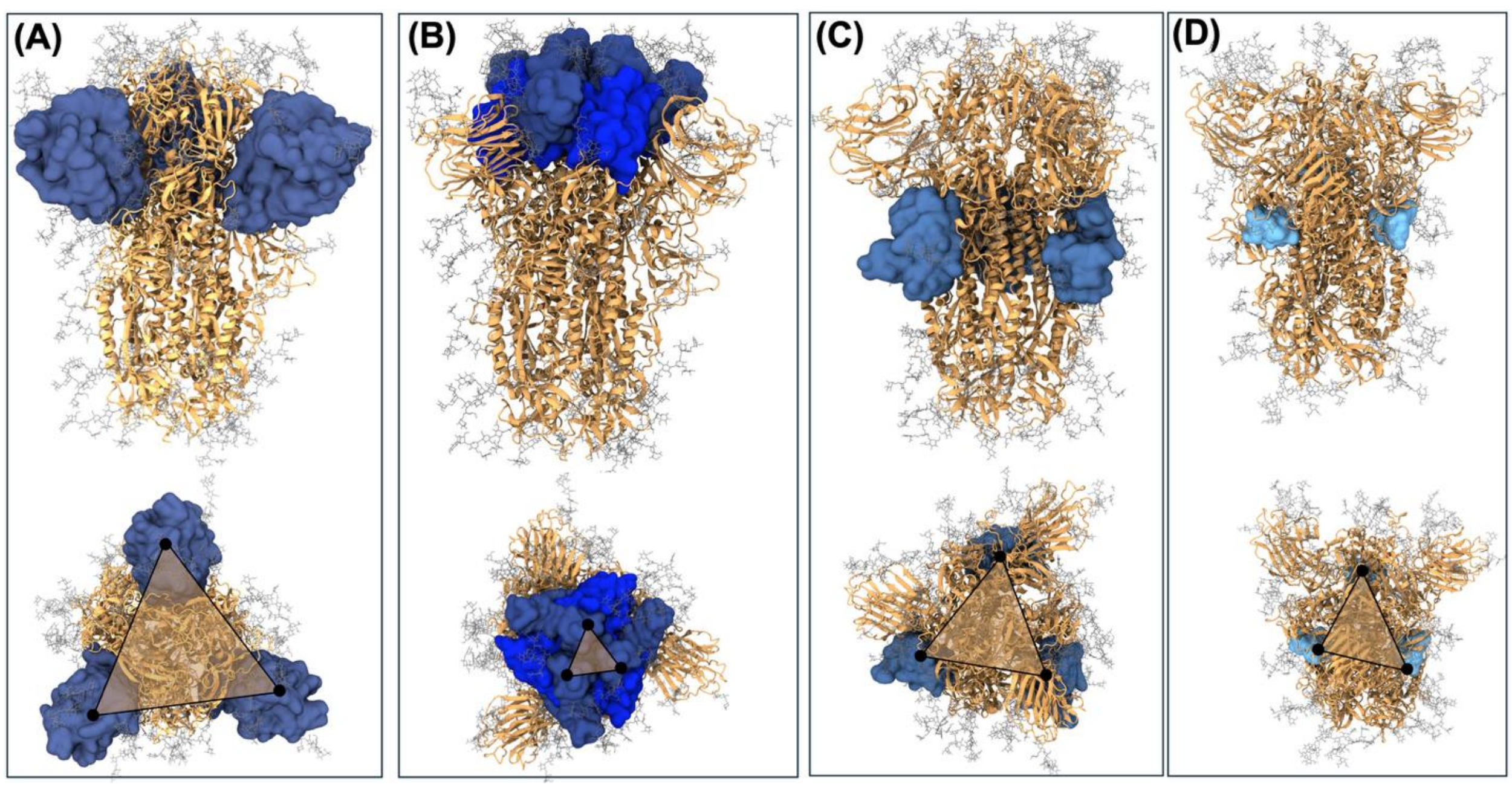

Figure S3: Trimeric area calculations of (A) NTD; N-terminal domain (B) RBM; Receptor binding domain (C) SD2; Sub-domain 2 (D) FPPR; Fusion Peptide Proximal Region

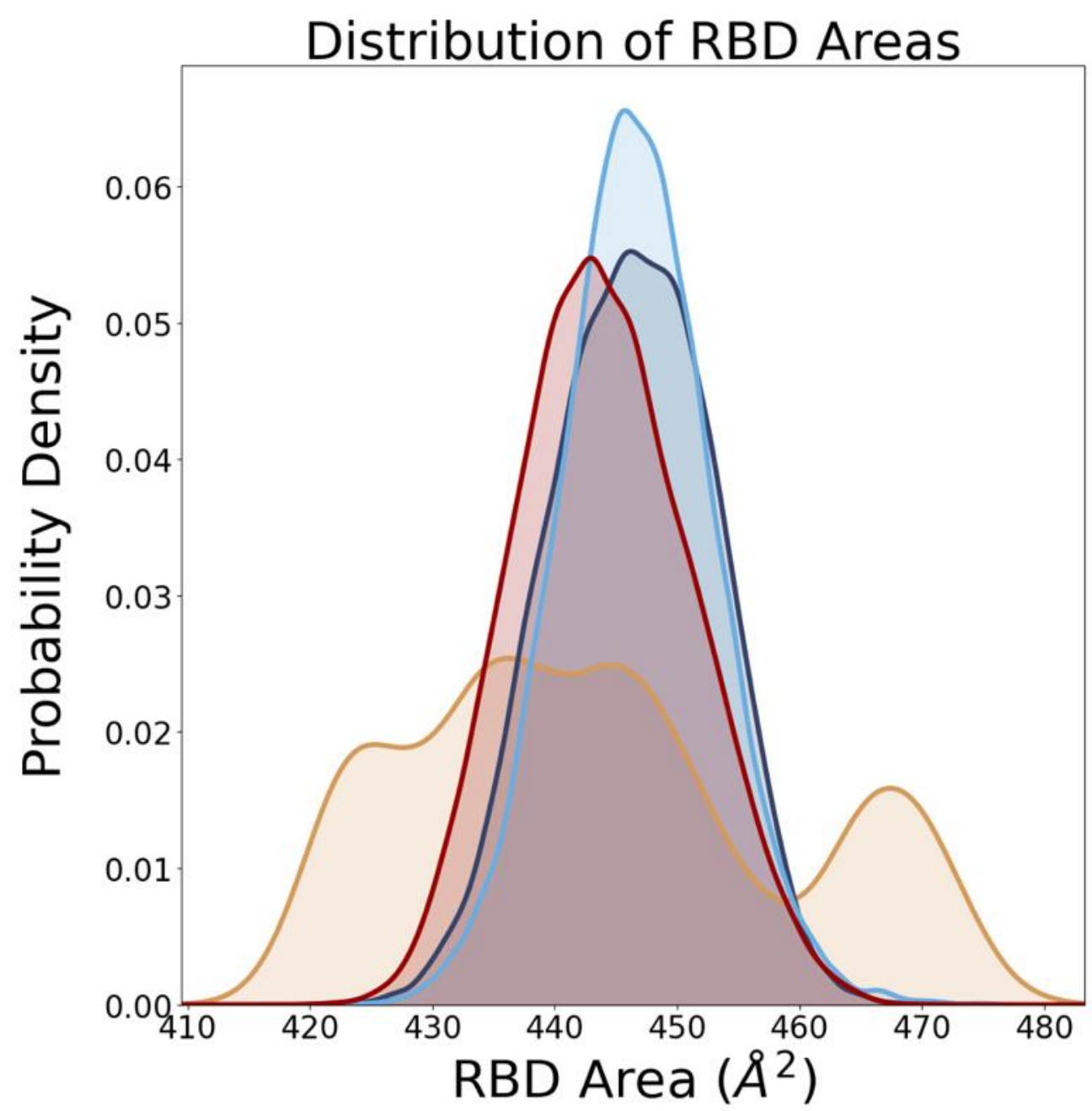

Figure S4: RBD trimeric area over the course of the simulations. WT is shown in gold, F294L in dark blue, A835D in cyan, and DM in red.

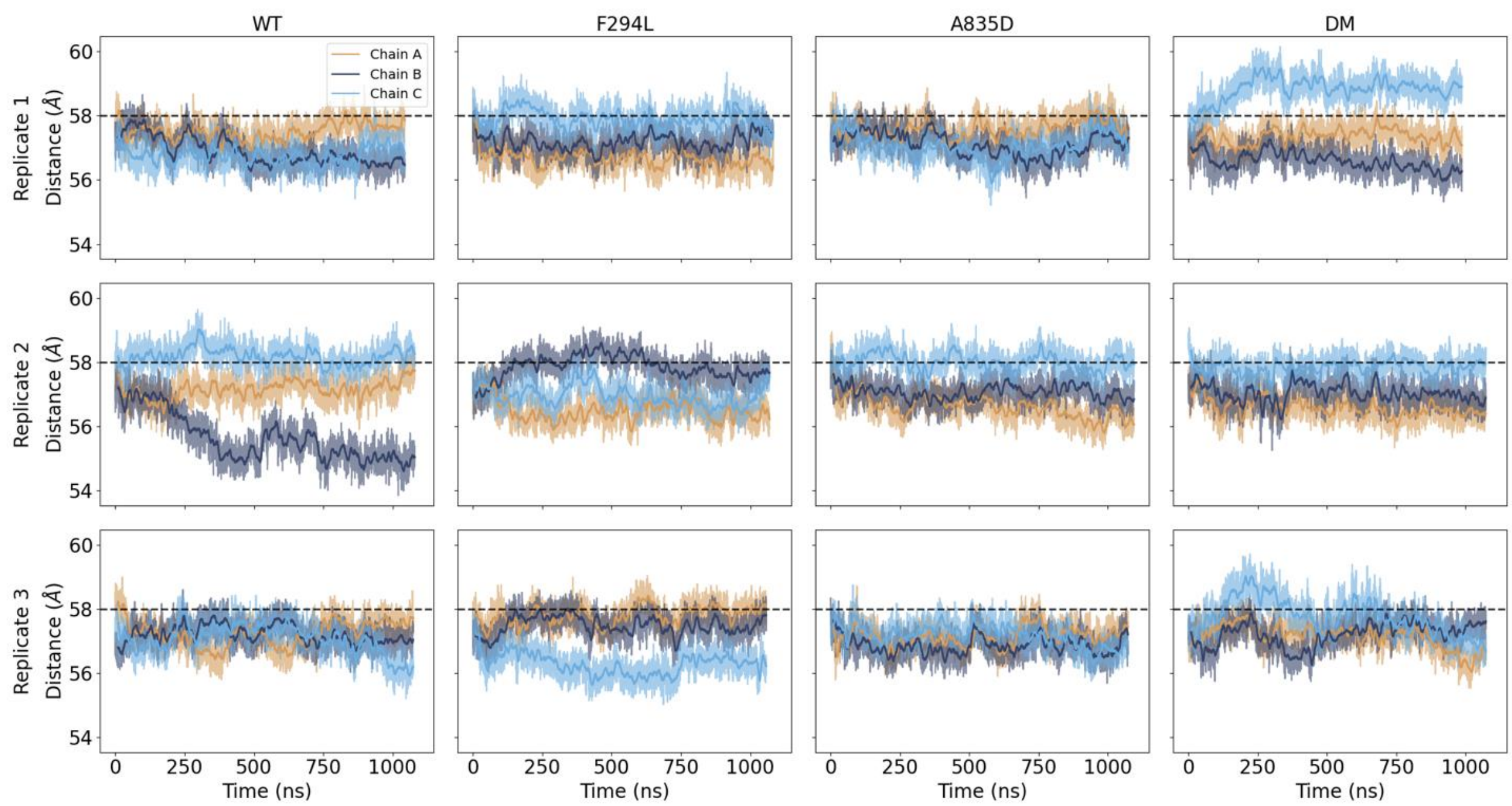

Figure S5: Time series analysis of RBD-core distances per replica across the variants depicting the propensity of RBD opening in each chain. Chain A is shown in gold, chain B in dark blue and chain C in cyan.

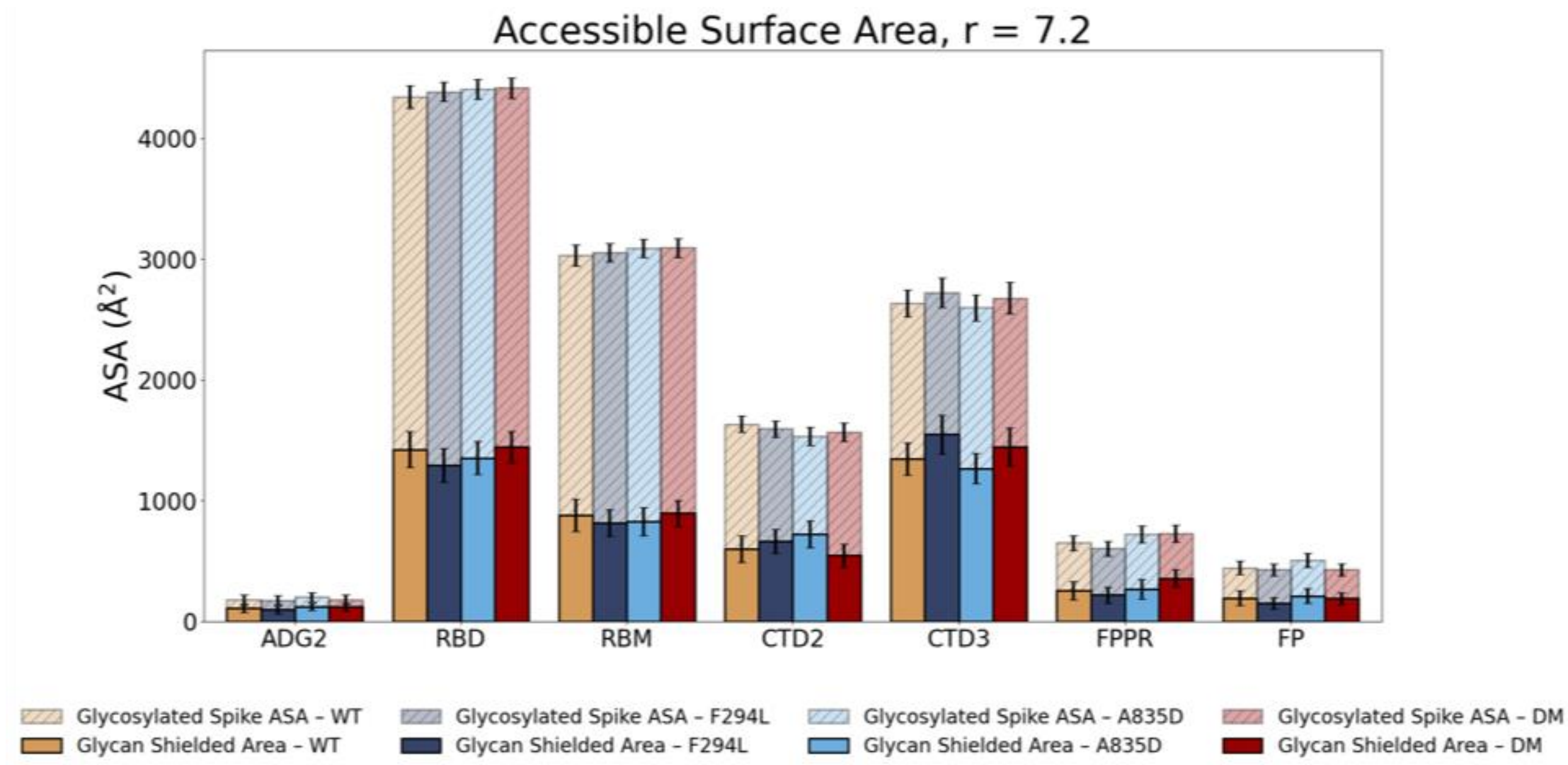

Figure S6. Accessible surface area of SHC014 spike domains with a probe radius of 7.2Å. ADG2 referred to the ADG2 binding site.

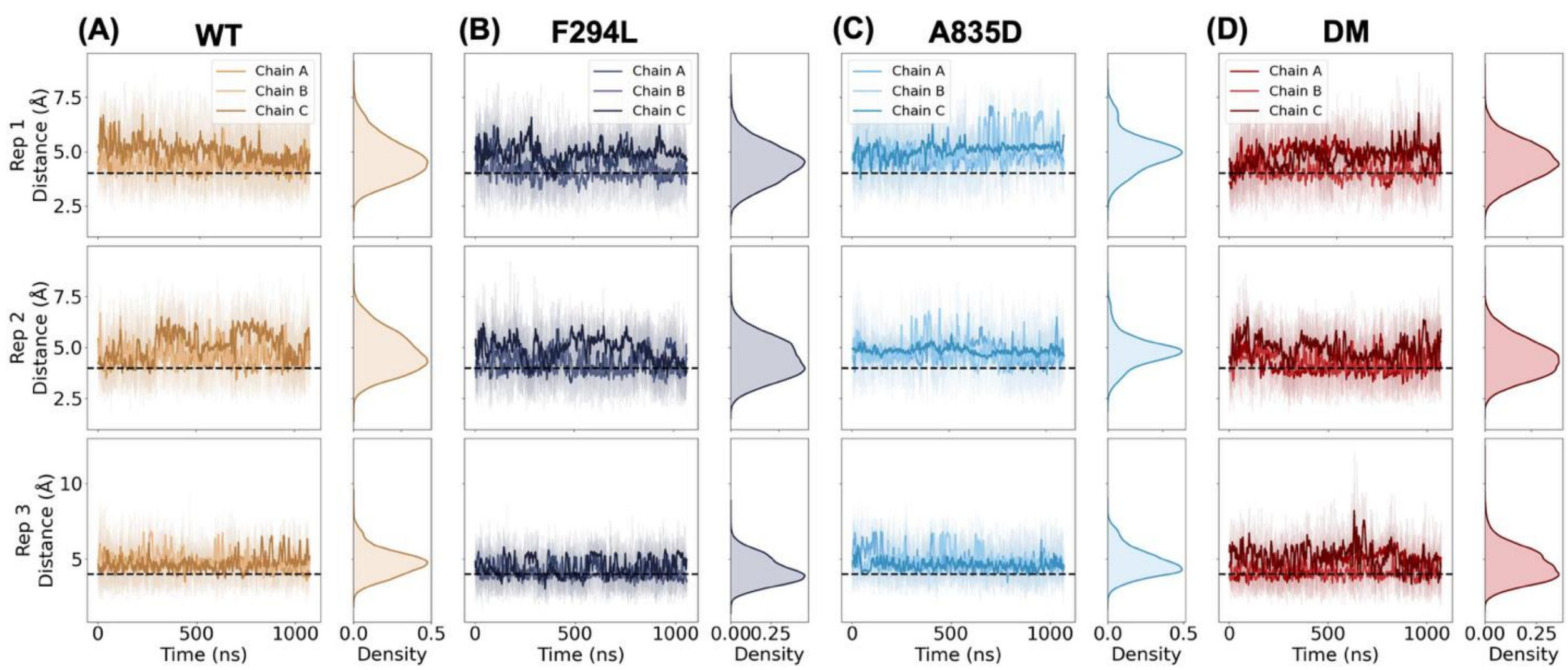

Figure S7: F294/L294-K266 contact analysis across the variants with a distance threshold of 4Å shown as dashed lines.

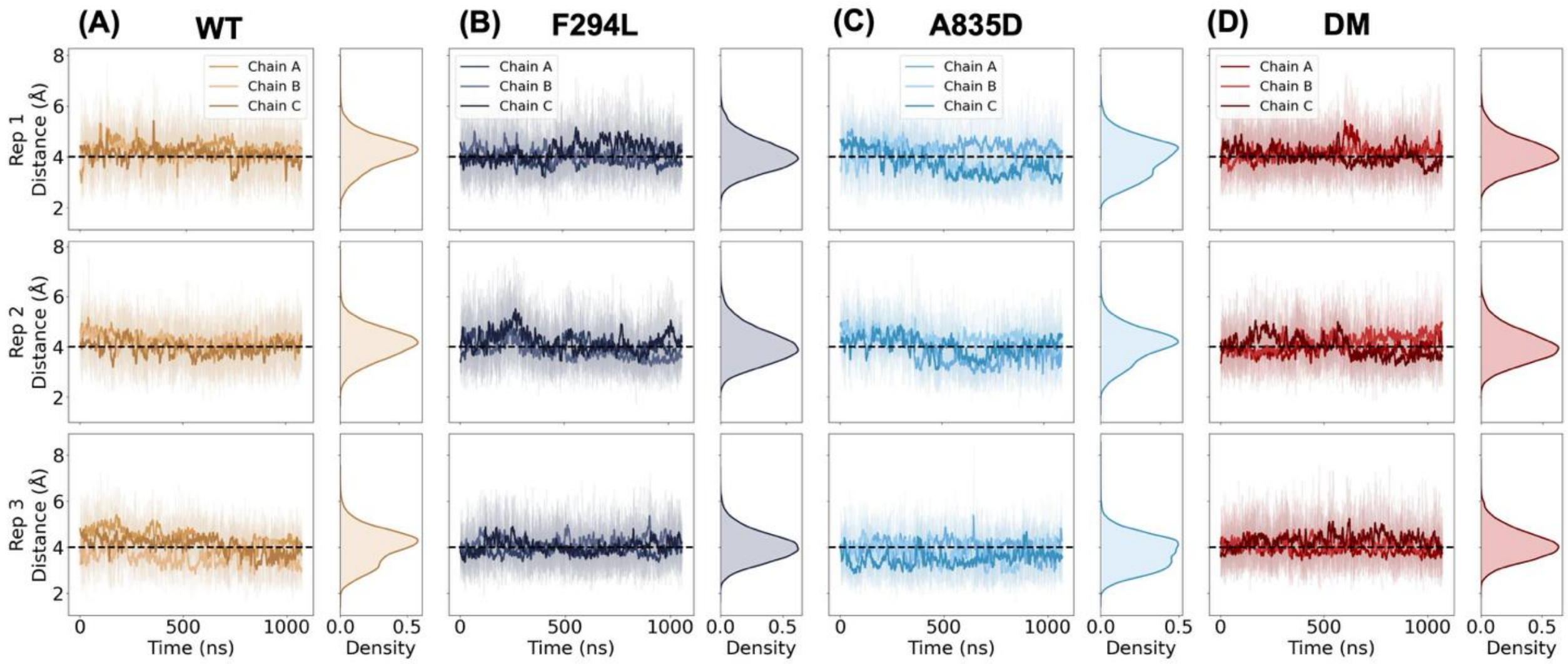

Figure S8: F294/L294-K288 contact analysis across the variants with a distance threshold of 4Å shown as dashed lines

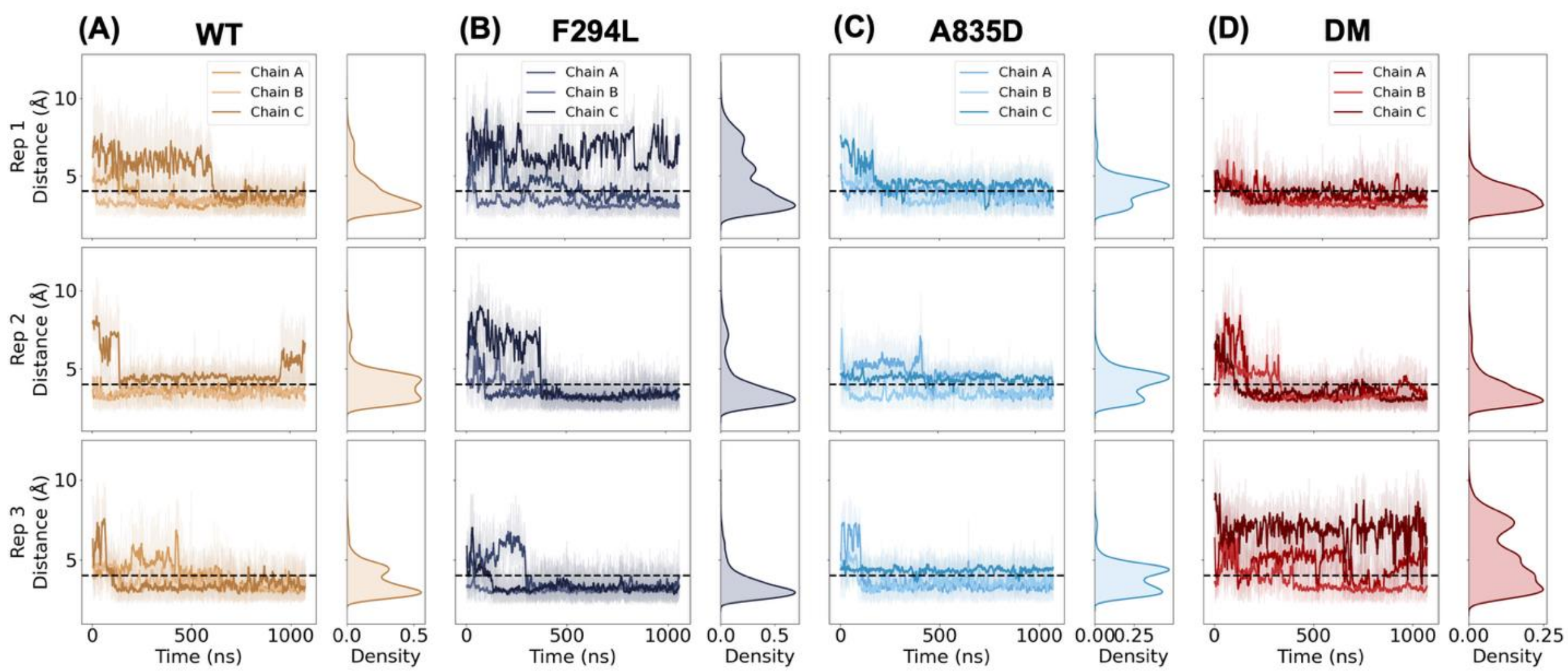

Figure S9: F294/L294-D275 contact analysis across the variants with a distance threshold of 4Å shown as dashed lines.

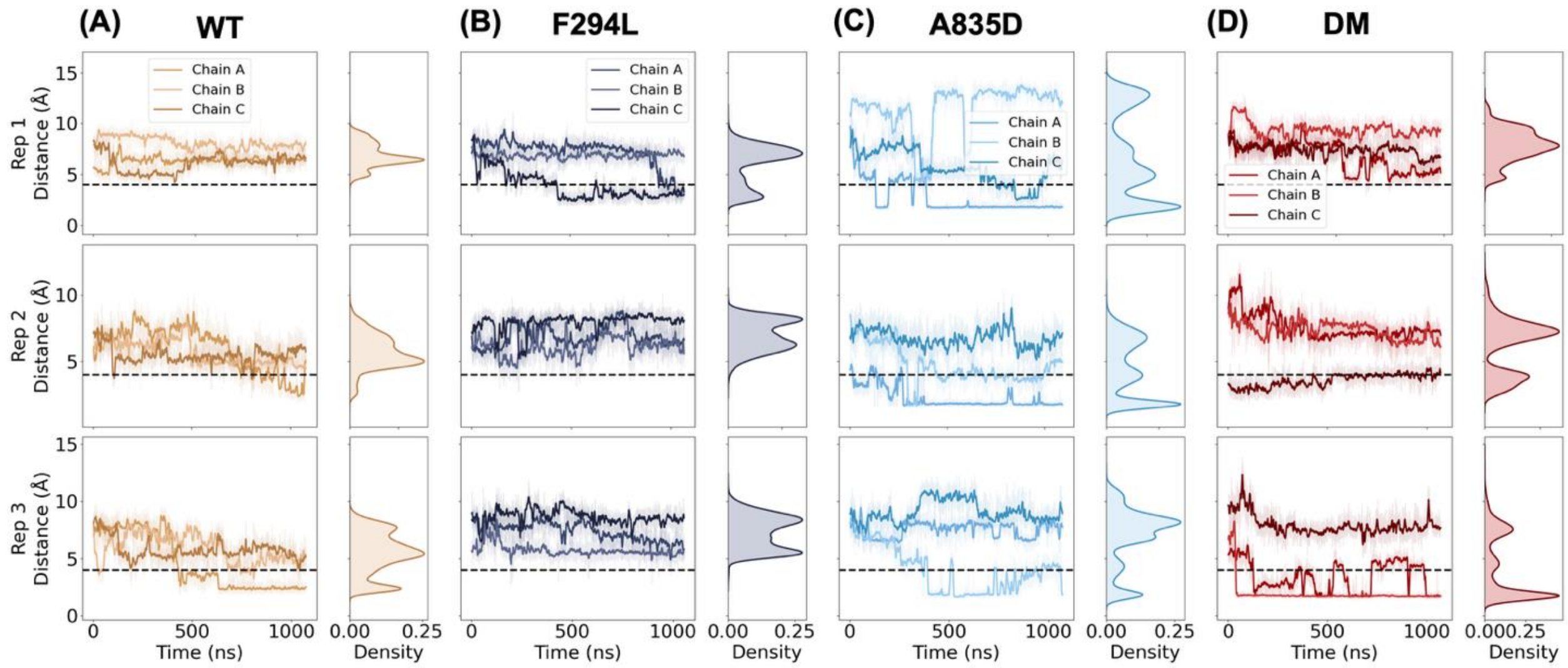

Figure S10: A835/D835-R830 contact analysis across the variants with a distance threshold of 4Å shown as dashed lines.

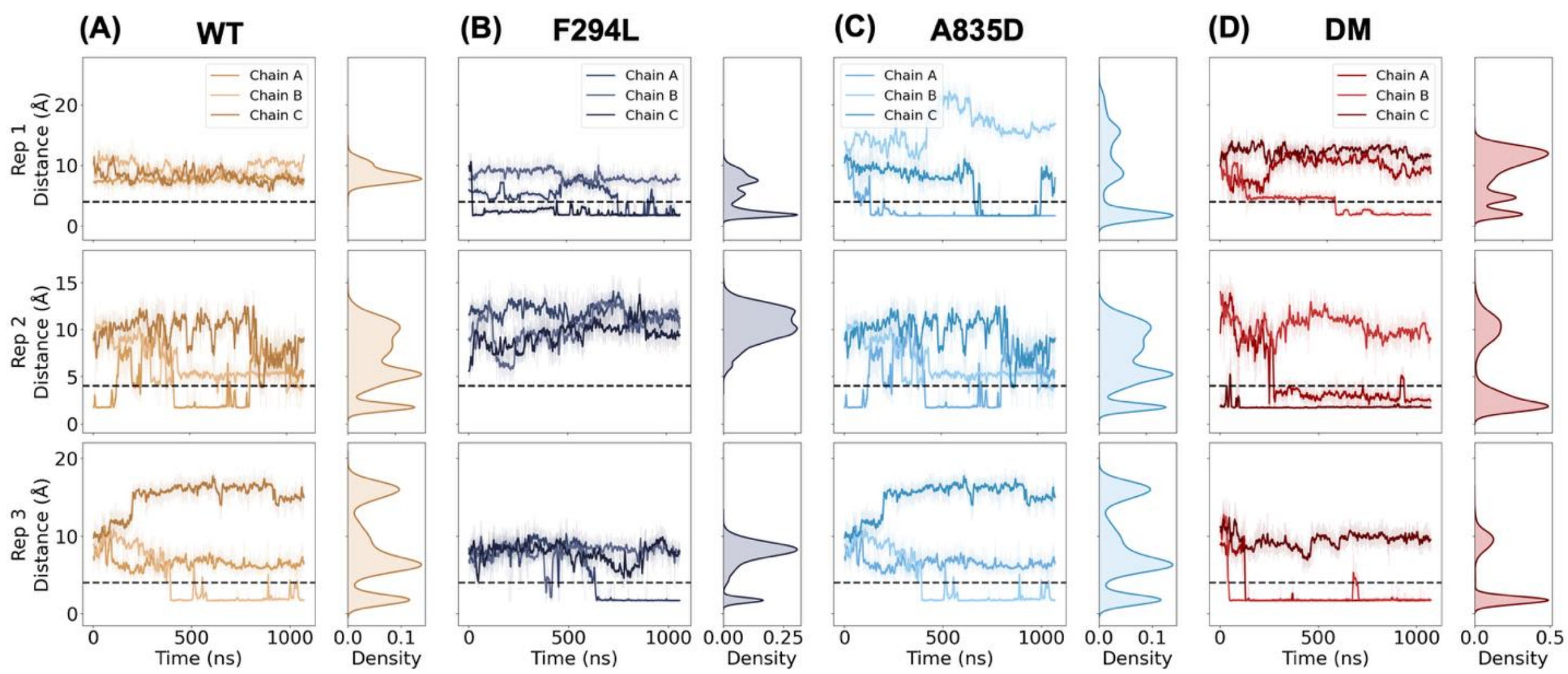

Figure S11: R830-D555 contact analysis across the variants with a distance threshold of 4Å shown as dashed lines.

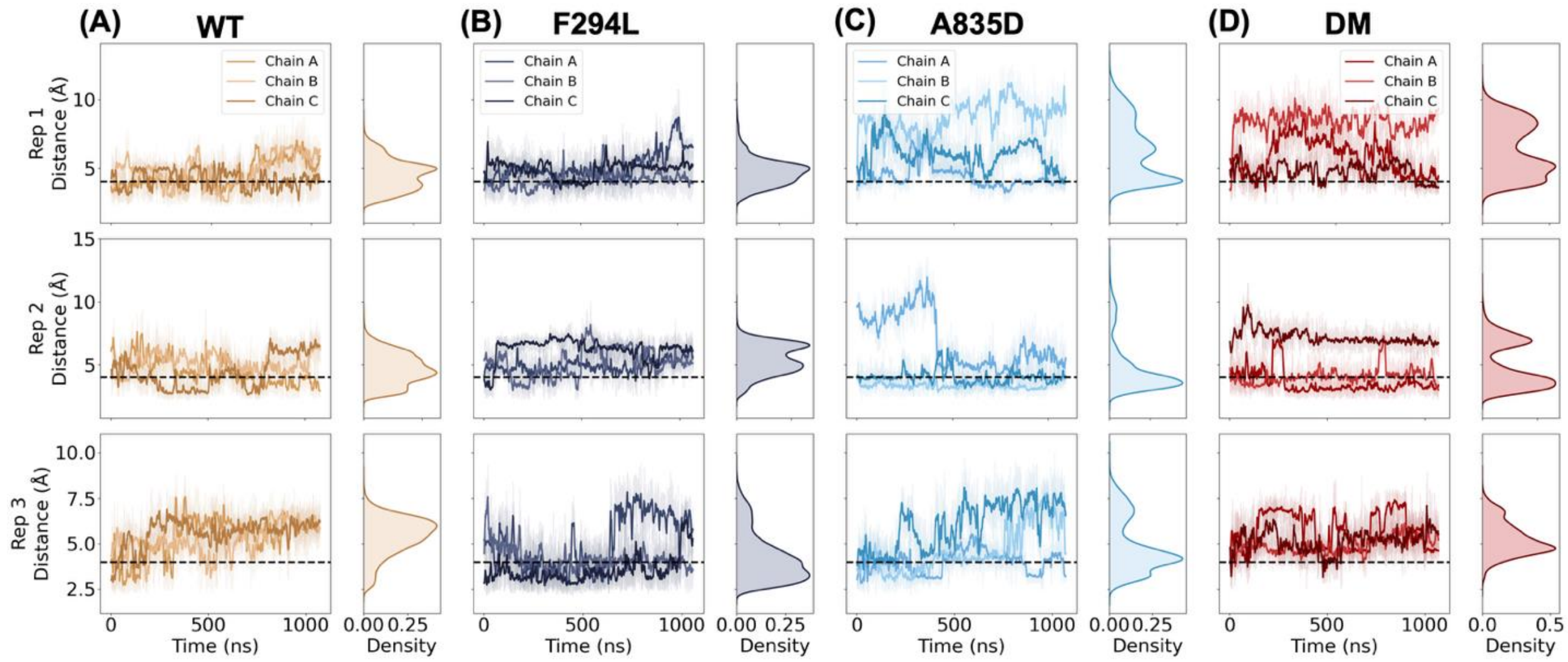

Figure S12: A835/D835-D555 contact analysis across the variants with a distance threshold of 4Å shown as dashed lines.

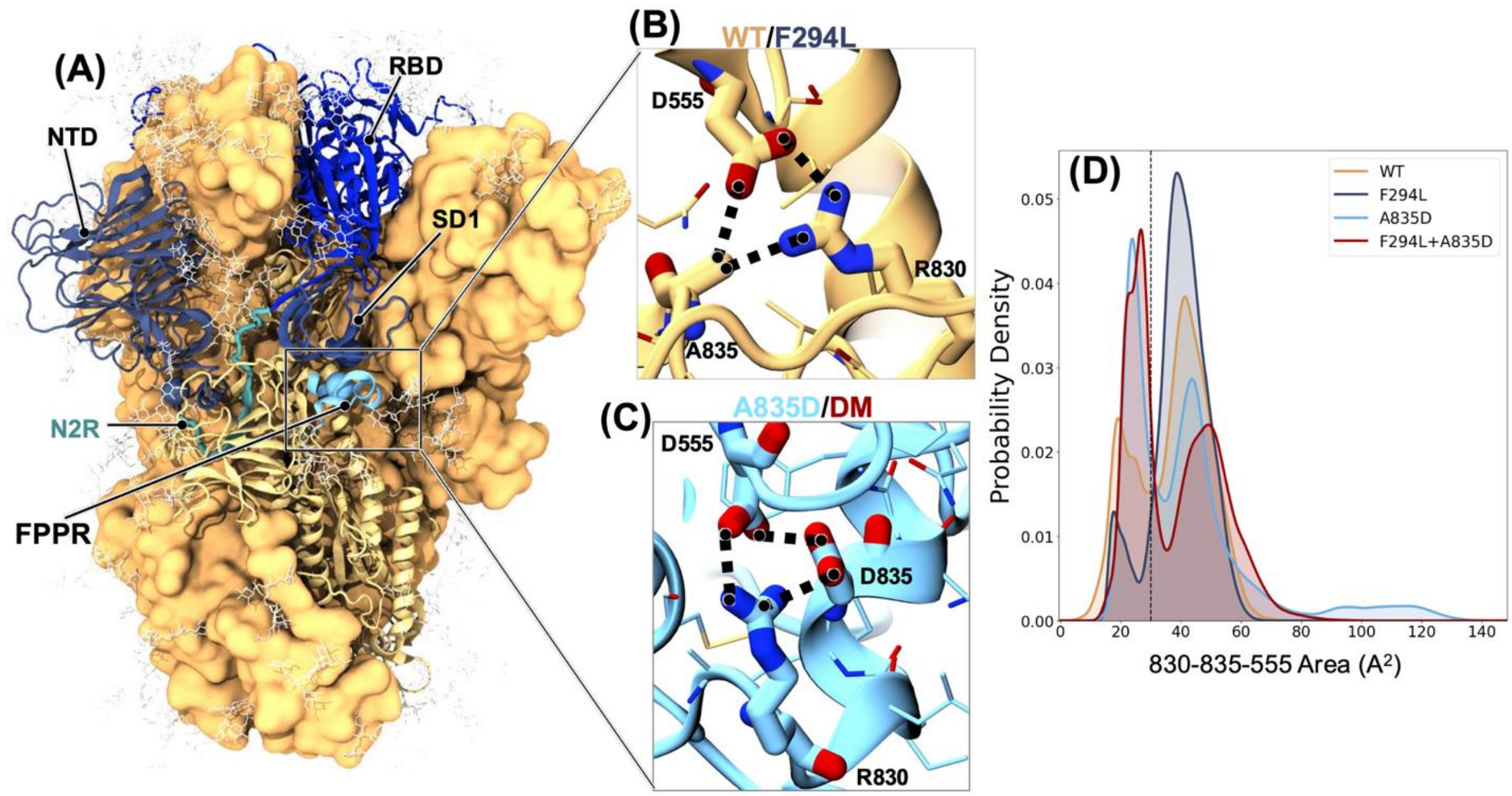

Figure S13. Salt bridge formation (A) Highlight of different domains in the SHC014 spike ectodomain, which includes the NTD: N-terminal domain; RBD: Receptor binding domain; SD1: Subdomain-1; N2R Linker and FPPR: Fusion peptide proximal region. Position 835 is shown as a cyan sphere matching the A835D color (B). Close view of A835-R830-D555 area (C) Close view of D835-R830-D555 area (D). Formation and persistence of the salt-bridge triad between R830-D555-X835 with a tight salt-bridge having a threshold of <30Å^2 shown as a broken line. X = A835 or D835.

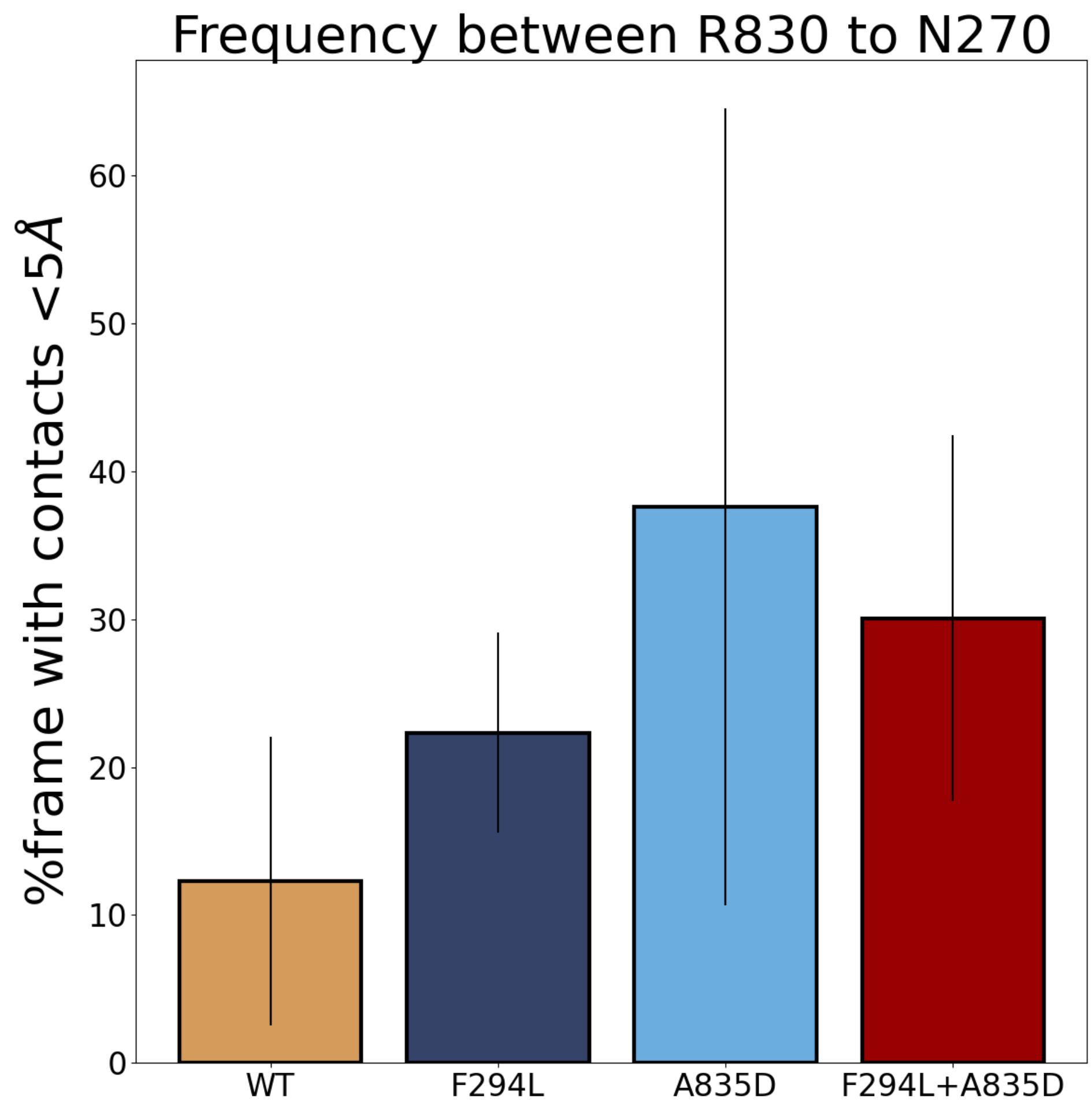

Figure S14. Frequency of contact across variants between R830 and glycan N270.

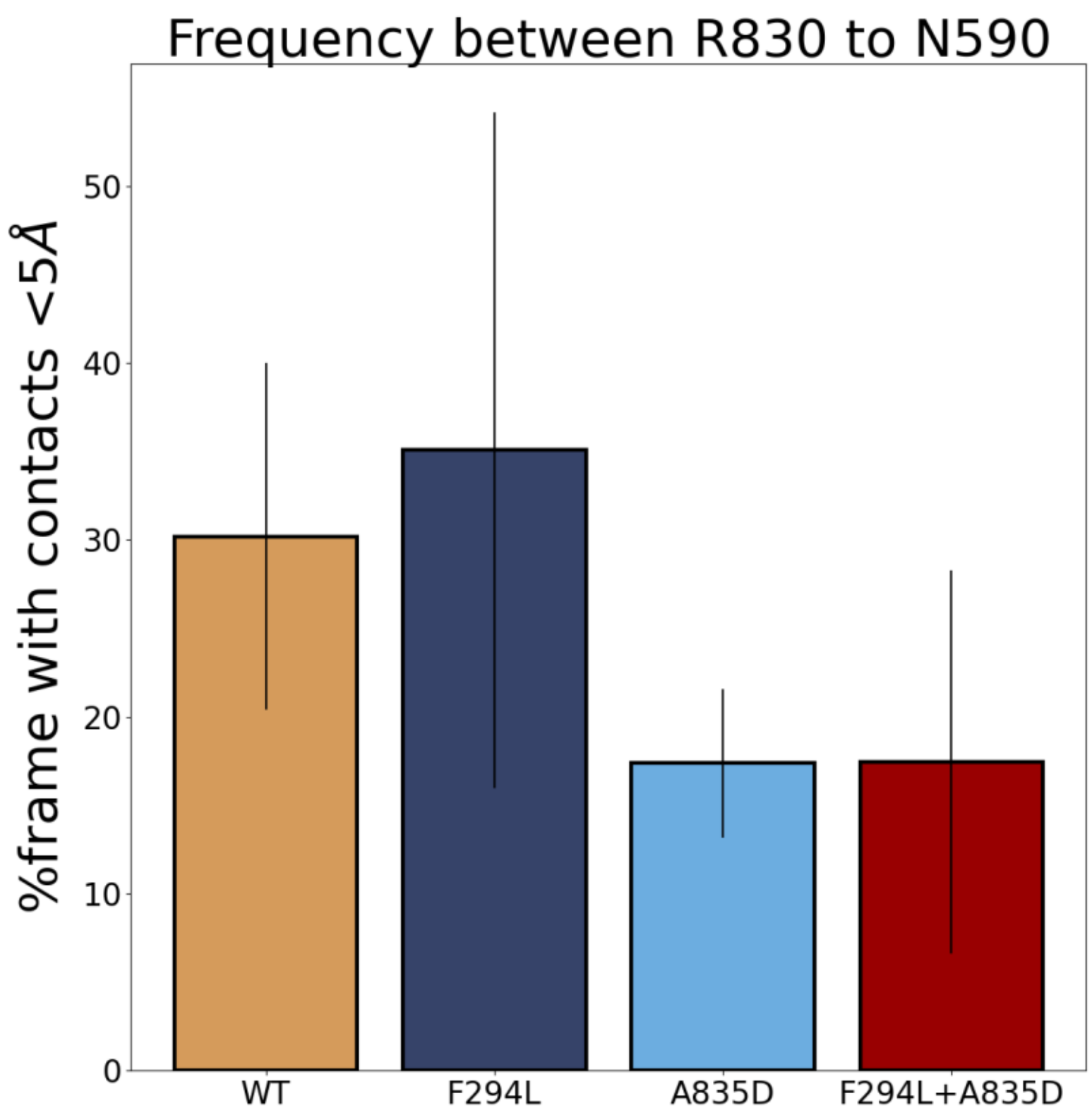

Figure S15. Frequency of contact across variants between R830 and glycan N590.

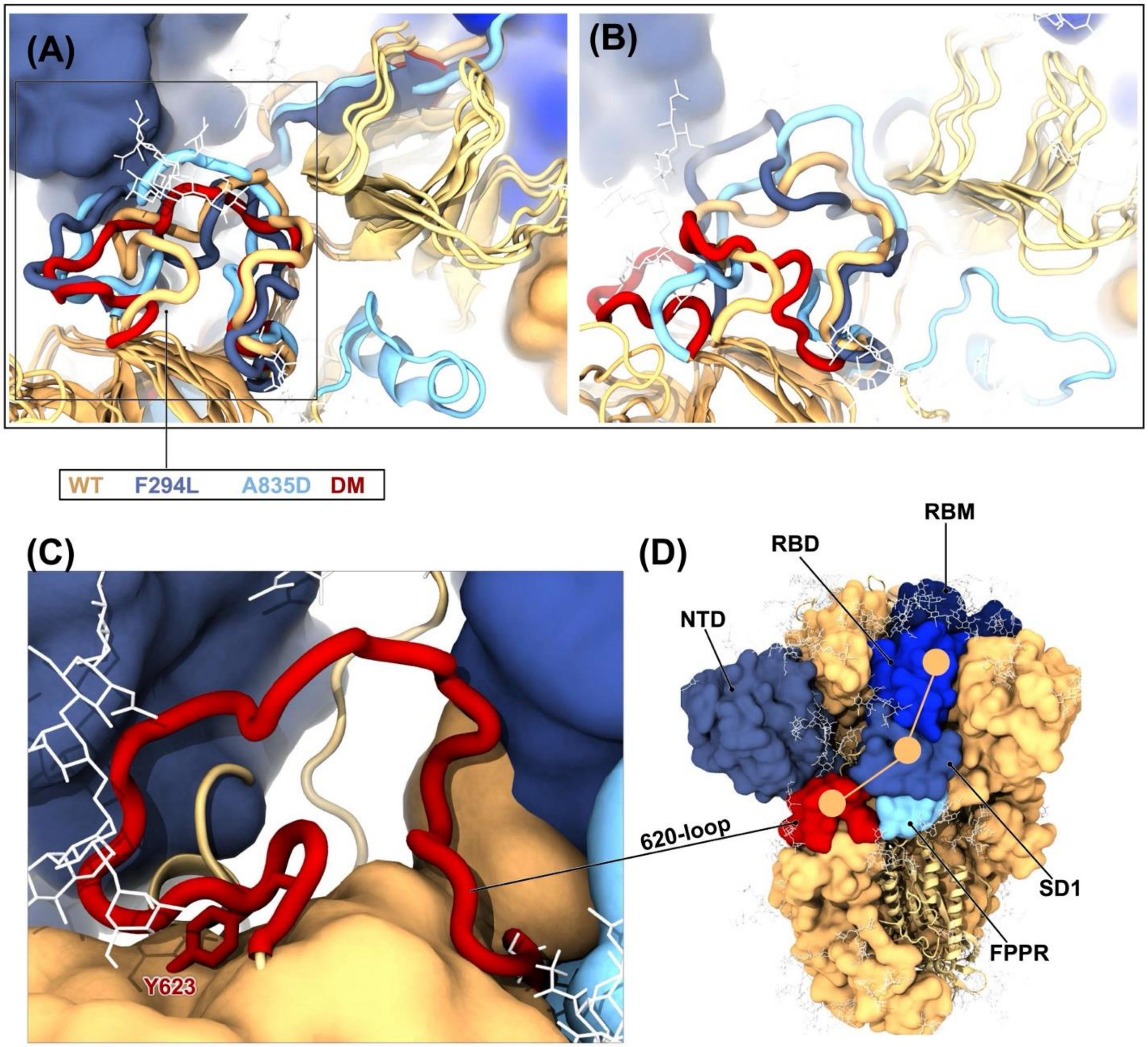

Figure S16. Conformational flexibility of the 620-loop across the variants (A). Similar conformation of the 620-loop across the variants (B). Varying degrees in the conformational dynamics of the 620-loop with the double mutant (shown in red), 620-loop showing the highest degree of flexibility (C). A close view of Y623 within the 620loop (D). A proposed model of allosteric communication is depicted by connecting circles from the 620loop through sub-domain-1 to the RBD.

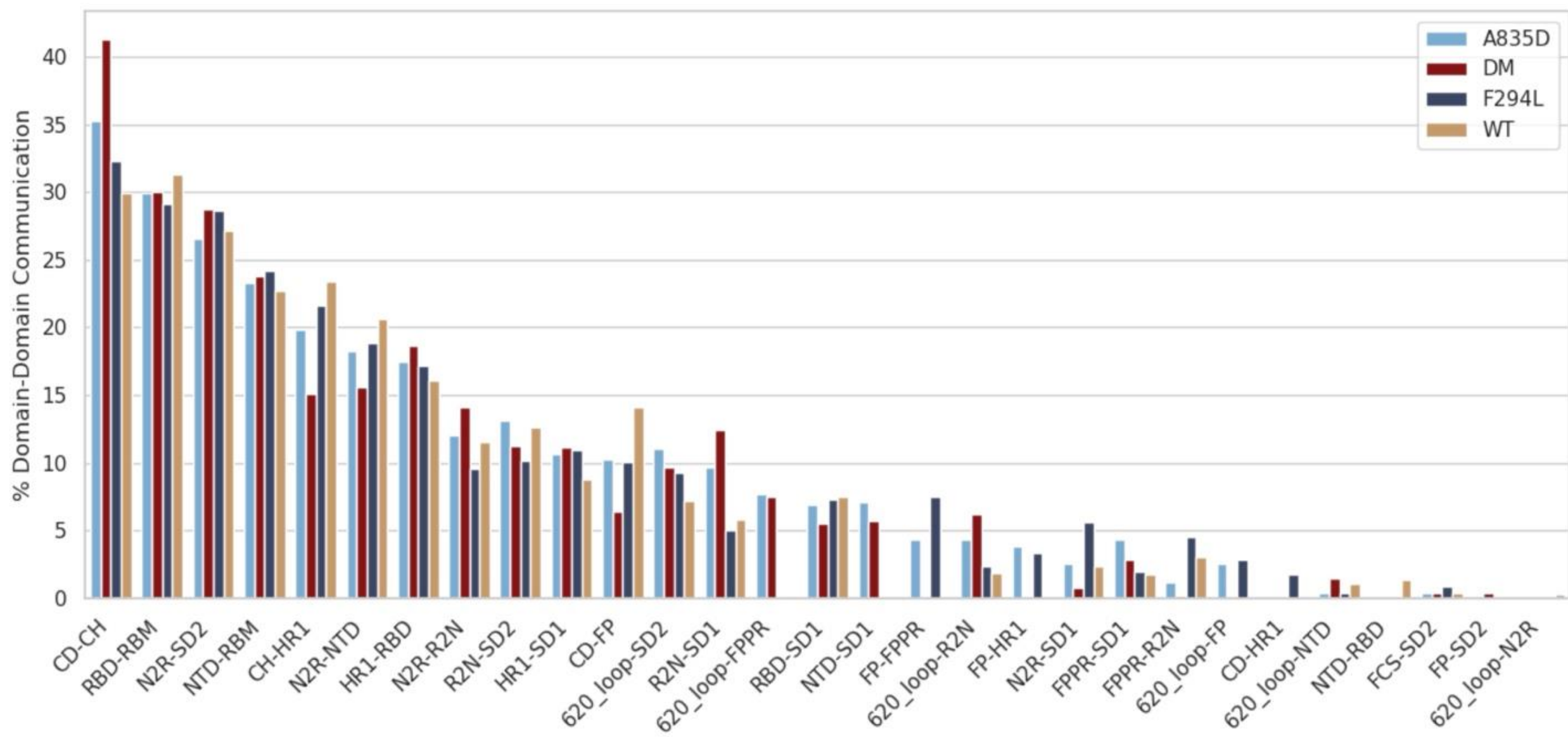

Figure S17. Domain-domain communication across the SHC014 spike variants.

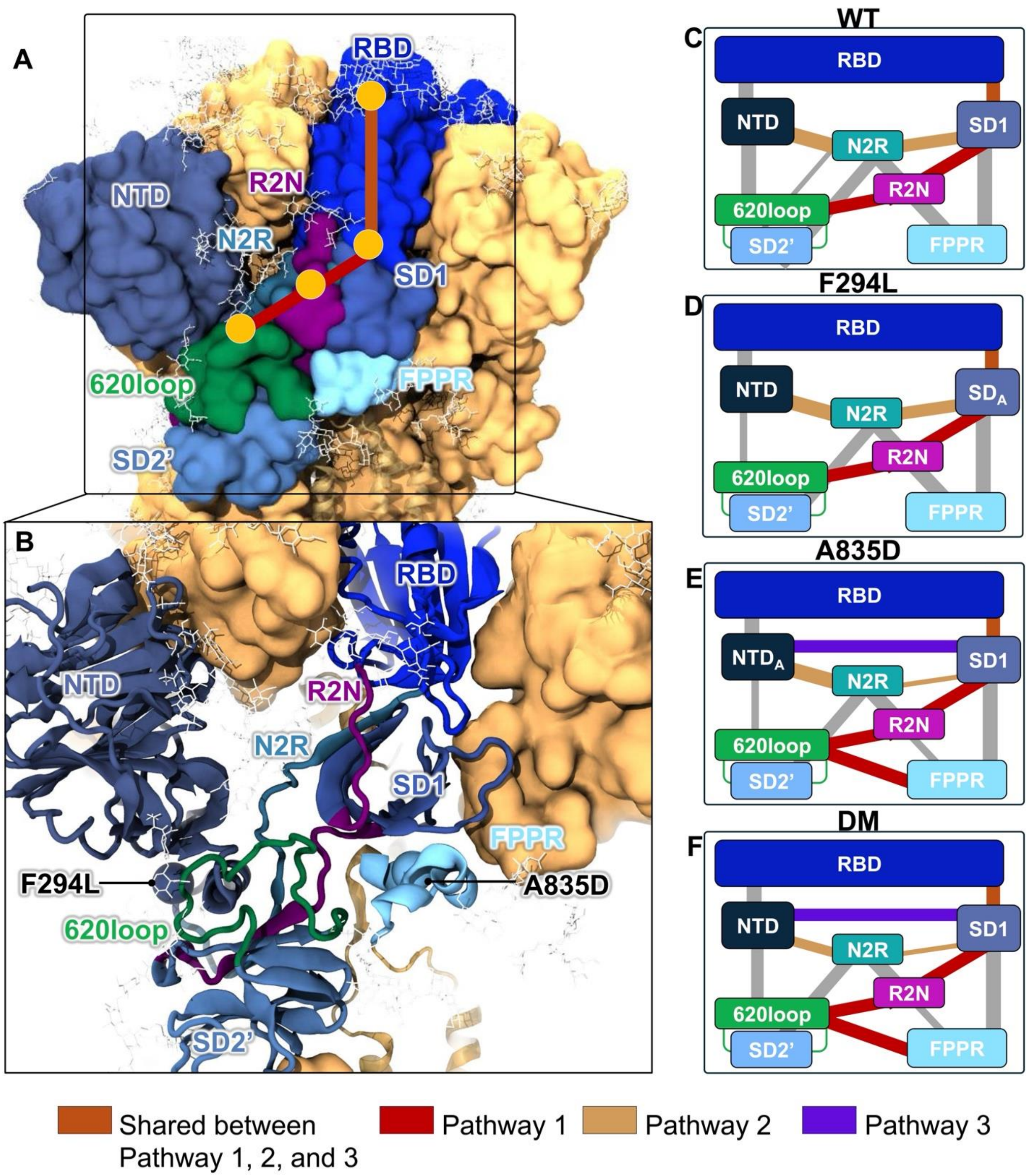

Figure S18. Allosteric communication pathways in SHC014 variants. Non-primary pathways are shown in gray in C-F.

Supplementary Tables

Table S1: Complete details of SHC014 computational models and simulation information:

	**WT**	**F294L**	**A835D**	**DM**
Total #atoms	972591	972585	972600	972588
#protein atoms/residues	50928 / 308316	50925 / 308315	50934 / 308319	50931 / 308316
#glycan atoms/glycans	11415 / 57	11415 / 57	11415 / 57	11415 / 57
#water atoms/residues	908598 / 302866	908595 / 302865	908598 / 302866	908589 / 302863
#Na/Cl ions	843 / 807	843 / 807	846 / 807	846 / 807
Disulfides per monomer/ Total disulfide bonds	15 / 45	15 / 45	15 / 45	15/45
System Dimensions (ÅxÅxÅ)	215.00 X 214.99 X 214.98	215.00 X 214.99 X 214.98	215.00 X 214.99 X 214.98	215.00 X 214.99 X 214.98

Table S2: Summary of the full-length S protein all-atom MD simulations.

**Variant**	**Rep1 (ns)**	**Rep2 (ns)**	**Rep3 (ns)**
WT	1042.3	1078.3	1074.2
F294L	1080.3	1068.8	1056.8
A835D	1073.8	1093.2	1072
DM	1075.6	1072	1073.1

Table S3. Glycan compositions for chains A, B, and C.

CHAIN A

#	SITE	TYPE	STRUCTURE	SEQUENCE
G1	N66	M5	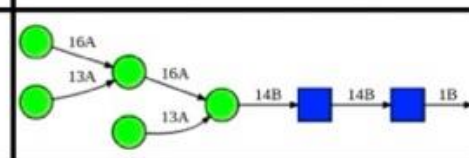	aDMan(1→6)[aDMan(1→3)]aDMan(1→6)<br>[aDMan(1→3)]bDMan(1→4)bDGlcNAc(1→4)bDGlcNAc(1→)PROA-66
G2	N110	FA2G2S1	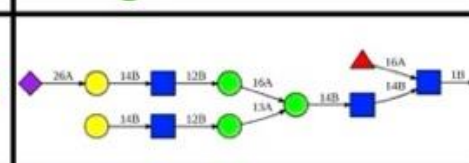	aDNeu5Ac(2→6)bDGal(1→4)bDGlcNAc(1→2)aDMan(1→6)<br>[bDGal(1→4)bDGlcNAc(1→2)aDMan(1→3)]bDMan(1→4)bDGlcNAc(1→4)<br>[aLFuc(1→6)]bDGlcNAc(1→)PROA-110
G3	N120	M5	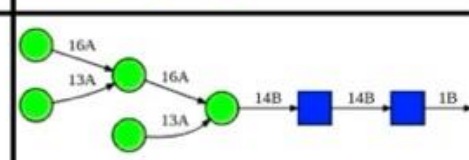	aDMan(1→6)[aDMan(1→3)]aDMan(1→6)<br>[aDMan(1→3)]bDMan(1→4)bDGlcNAc(1→4)bDGlcNAc(1→)PROA-120
G4	N145	FA2G2S1	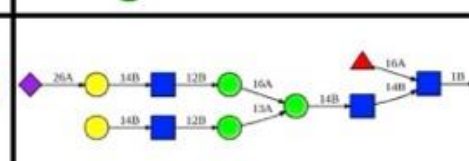	aDNeu5Ac(2→6)bDGal(1→4)bDGlcNAc(1→2)aDMan(1→6)<br>[bDGal(1→4)bDGlcNAc(1→2)aDMan(1→3)]bDMan(1→4)bDGlcNAc(1→4)<br>[aLFuc(1→6)]bDGlcNAc(1→)PROA-145
G5	N159	FA2G2S2	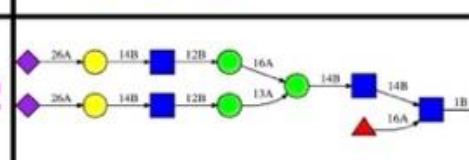	aDNeu5Ac(2→6)bDGal(1→4)bDGlcNAc(1→2)aDMan(1→6)<br>[aDNeu5Ac(2→6)bDGal(1→4)bDGlcNAc(1→2)aDMan(1→3)]bDMan(1→4)bDGlcNAc(1→4)<br>[aLFuc(1→6)]bDGlcNAc(1→)PROA-159
G6	N228	M8	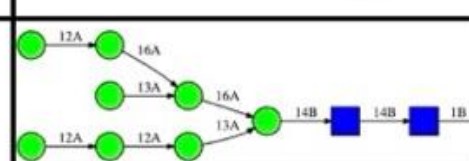	aDMan(1→2)aDMan(1→6)[aDMan(1→3)]aDMan(1→6)<br>[aDMan(1→2)aDMan(1→2)aDMan(1→3)]bDMan(1→4)bDGlcNAc(1→4)bDGlcNAc(1→)PROA-228
G7	N270	FA3	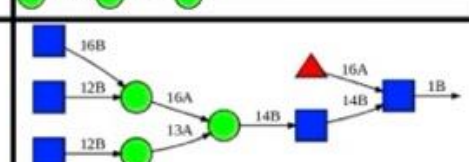	bDGlcNAc(1→6)[bDGlcNAc(1→2)]aDMan(1→6)<br>[bDGlcNAc(1→2)aDMan(1→3)]bDMan(1→4)bDGlcNAc(1→4)[aLFuc(1→6)]bDGlcNAc(1→)PROA-270
G8	N319	FA2	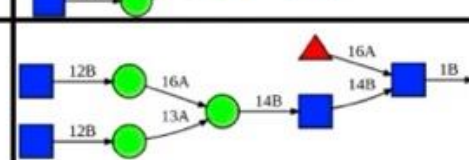	bDGlcNAc(1→2)aDMan(1→6)[bDGlcNAc(1→2)aDMan(1→3)]bDMan(1→4)bDGlcNAc(1→4)<br>[aLFuc(1→6)]bDGlcNAc(1→)PROA-319
G9	N331	FA2	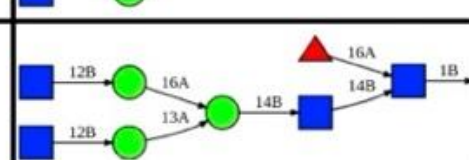	bDGlcNAc(1→2)aDMan(1→6)[bDGlcNAc(1→2)aDMan(1→3)]bDMan(1→4)bDGlcNAc(1→4)<br>[aLFuc(1→6)]bDGlcNAc(1→)PROA-331
G10	N358	FA2G2S1	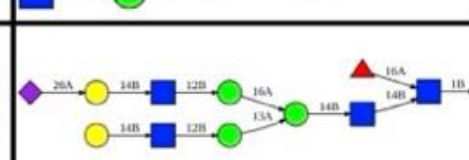	aDNeu5Ac(2→6)bDGal(1→4)bDGlcNAc(1→2)aDMan(1→6)<br>[bDGal(1→4)bDGlcNAc(1→2)aDMan(1→3)]bDMan(1→4)bDGlcNAc(1→4)<br>[aLFuc(1→6)]bDGlcNAc(1→)PROA-358
G11	N590	FA2	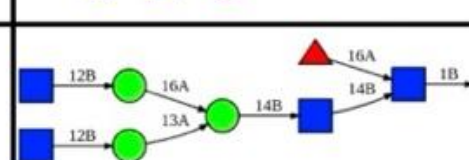	bDGlcNAc(1→2)aDMan(1→6)[bDGlcNAc(1→2)aDMan(1→3)]bDMan(1→4)bDGlcNAc(1→4)<br>[aLFuc(1→6)]bDGlcNAc(1→)PROA-590
G12	N603	A2	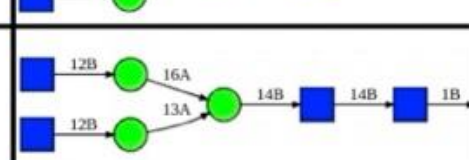	bDGlcNAc(1→2)aDMan(1→6)<br>[bDGlcNAc(1→2)aDMan(1→3)]bDMan(1→4)bDGlcNAc(1→4)bDGlcNAc(1→)PROA-603
G13	N692	M6	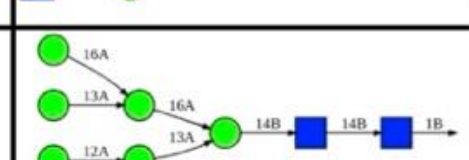	aDMan(1→6)[aDMan(1→3)]aDMan(1→6)<br>[aDMan(1→2)aDMan(1→3)]bDMan(1→4)bDGlcNAc(1→4)bDGlcNAc(1→)PROA-692
G14	N700	Hybrid G1	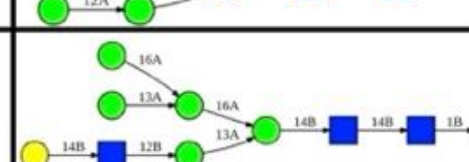	bDGal(1→4)bDGlcNAc(1→2)aDMan(1→3)[aDMan(1→6)<br>[aDMan(1→3)]aDMan(1→6)]bDMan(1→4)bDGlcNAc(1→4)bDGlcNAc(1→)PROA-700
G15	N784	M6	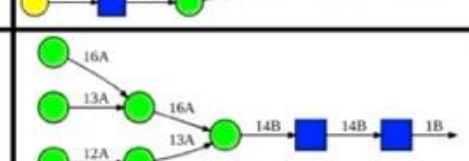	aDMan(1→6)[aDMan(1→3)]aDMan(1→6)<br>[aDMan(1→2)aDMan(1→3)]bDMan(1→4)bDGlcNAc(1→4)bDGlcNAc(1→)PROA-784
G16	N1057	FA2G2S1	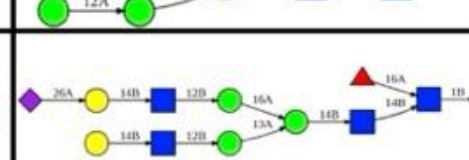	aDNeu5Ac(2→6)bDGal(1→4)bDGlcNAc(1→2)aDMan(1→6)<br>[bDGal(1→4)bDGlcNAc(1→2)aDMan(1→3)]bDMan(1→4)bDGlcNAc(1→4)<br>[aLFuc(1→6)]bDGlcNAc(1→)PROA-1057
G17	N1081	FA2	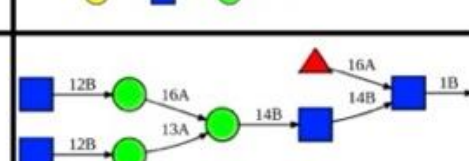	bDGlcNAc(1→2)aDMan(1→6)[bDGlcNAc(1→2)aDMan(1→3)]bDMan(1→4)bDGlcNAc(1→4)<br>[aLFuc(1→6)]bDGlcNAc(1→)PROA-1081
G18	N1117	FA1	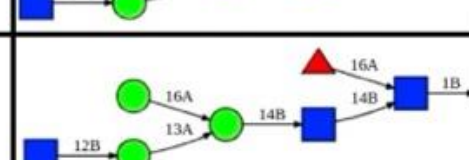	bDGlcNAc(1→2)aDMan(1→3)[aDMan(1→6)]bDMan(1→4)bDGlcNAc(1→4)<br>[aLFuc(1→6)]bDGlcNAc(1→)PROB-1117
G19	S311	O-glycan	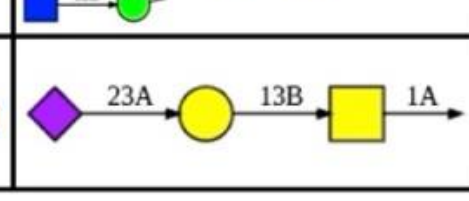	aDNeu5Ac(2→3)bDGal(1→3)aDGalNAc(1→)PROA-311

CHAIN B

#	SITE	TYPE	STRUCTURE	SEQUENCE
G20	N66	M5	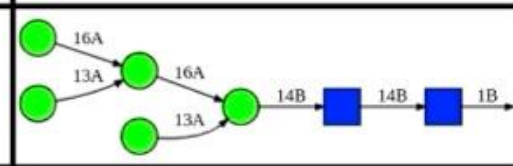	aDMan(1→6)[aDMan(1→3)]aDMan(1→6)<br>[aDMan(1→3)]bDMan(1→4)bDGlcNAc(1→4)bDGlcNAc(1→)PROB-66
G21	N110	FA2G2S2	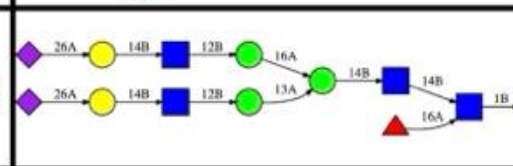	aDNeu5Ac(2→6)bDGal(1→4)bDGlcNAc(1→2)aDMan(1→6)<br>[aDNeu5Ac(2→6)bDGal(1→4)bDGlcNAc(1→2)aDMan(1→3)]bDMan(1→4)bDGlcNAc(1→4)<br>[aLFuc(1→6)]bDGlcNAc(1→)PROB-110
G22	N120	FA2	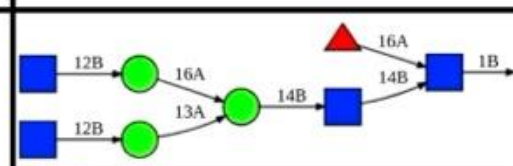	bDGlcNAc(1→2)aDMan(1→6)[bDGlcNAc(1→2)aDMan(1→3)]bDMan(1→4)bDGlcNAc(1→4)<br>[aLFuc(1→6)]bDGlcNAc(1→)PROB-120
G23	N145	FA3	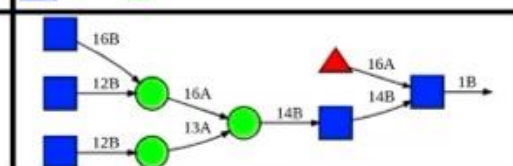	bDGlcNAc(1→6)[bDGlcNAc(1→2)]aDMan(1→6)<br>[bDGlcNAc(1→2)aDMan(1→3)]bDMan(1→4)bDGlcNAc(1→4)[aLFuc(1→6)]bDGlcNAc(1→)PROB-145
G24	N159	M5	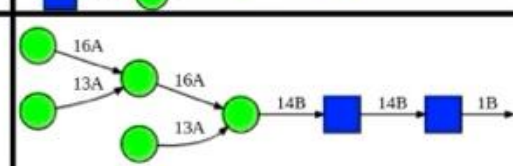	aDMan(1→6)[aDMan(1→3)]aDMan(1→6)<br>[aDMan(1→3)]bDMan(1→4)bDGlcNAc(1→4)bDGlcNAc(1→)PROB-159
G25	N228	M9	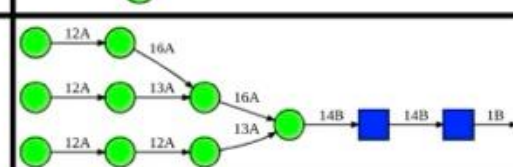	aDMan(1→2)aDMan(1→6)[aDMan(1→2)aDMan(1→3)]aDMan(1→6)<br>[aDMan(1→2)aDMan(1→2)aDMan(1→3)]bDMan(1→4)bDGlcNAc(1→4)bDGlcNAc(1→)PROB-228
G26	N270	FA3G3S1	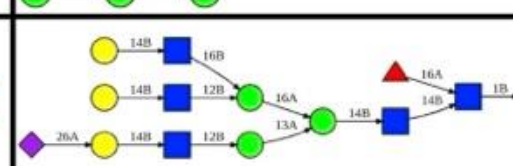	aDNeu5Ac(2→6)bDGal(1→4)bDGlcNAc(1→2)aDMan(1→3)[bDGal(1→4)bDGlcNAc(1→6)<br>[bDGal(1→4)bDGlcNAc(1→2)]aDMan(1→6)]bDMan(1→4)bDGlcNAc(1→4)<br>[aLFuc(1→6)]bDGlcNAc(1→)PROB-270
G27	N319	FA2	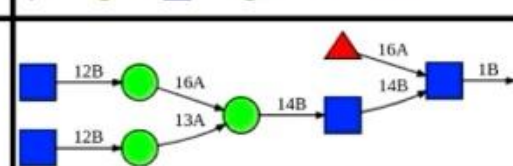	bDGlcNAc(1→2)aDMan(1→6)[bDGlcNAc(1→2)aDMan(1→3)]bDMan(1→4)bDGlcNAc(1→4)<br>[aLFuc(1→6)]bDGlcNAc(1→)PROB-319
G28	N331	FA1	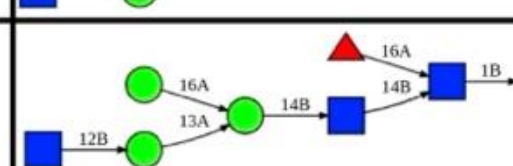	bDGlcNAc(1→2)aDMan(1→3)[aDMan(1→6)]bDMan(1→4)bDGlcNAc(1→4)<br>[aLFuc(1→6)]bDGlcNAc(1→)PROB-331
G29	N358	FA2G2S2	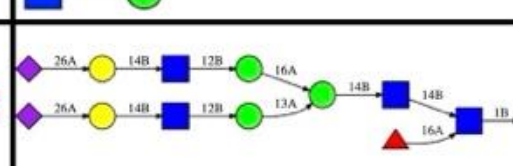	aDNeu5Ac(2→6)bDGal(1→4)bDGlcNAc(1→2)aDMan(1→6)<br>[aDNeu5Ac(2→6)bDGal(1→4)bDGlcNAc(1→2)aDMan(1→3)]bDMan(1→4)bDGlcNAc(1→4)<br>[aLFuc(1→6)]bDGlcNAc(1→)PROB-358
G30	N590	M5	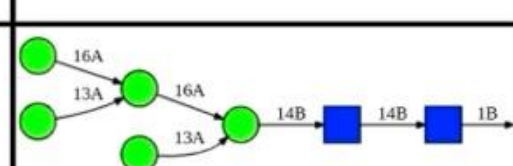	aDMan(1→6)[aDMan(1→3)]aDMan(1→6)<br>[aDMan(1→3)]bDMan(1→4)bDGlcNAc(1→4)bDGlcNAc(1→)PROB-590
G31	N603	FA2	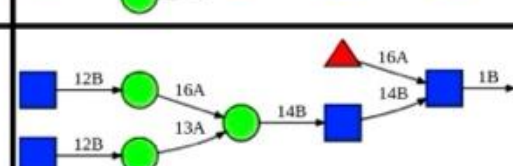	bDGlcNAc(1→2)aDMan(1→6)[bDGlcNAc(1→2)aDMan(1→3)]bDMan(1→4)bDGlcNAc(1→4)<br>[aLFuc(1→6)]bDGlcNAc(1→)PROB-603
G32	N692	M5	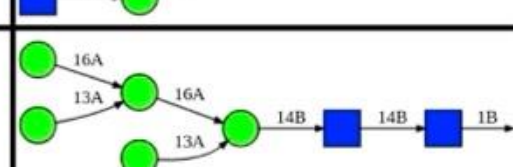	aDMan(1→6)[aDMan(1→3)]aDMan(1→6)<br>[aDMan(1→3)]bDMan(1→4)bDGlcNAc(1→4)bDGlcNAc(1→)PROB-692
G33	N700	M5	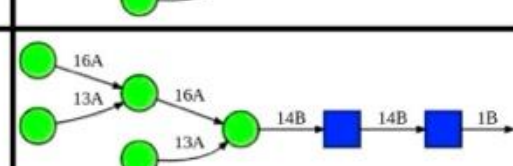	aDMan(1→6)[aDMan(1→3)]aDMan(1→6)<br>[aDMan(1→3)]bDMan(1→4)bDGlcNAc(1→4)bDGlcNAc(1→)PROB-700
G34	N784	M7	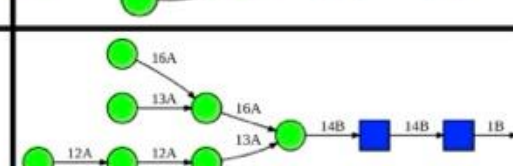	aDMan(1→2)aDMan(1→2)aDMan(1→3)[aDMan(1→6)<br>[aDMan(1→3)]aDMan(1→6)]bDMan(1→4)bDGlcNAc(1→4)bDGlcNAc(1→)PROB-784
G35	N1057	M5	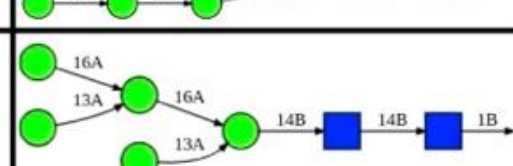	aDMan(1→6)[aDMan(1→3)]aDMan(1→6)<br>[aDMan(1→3)]bDMan(1→4)bDGlcNAc(1→4)bDGlcNAc(1→)PROB-1057
G36	N1081	A2	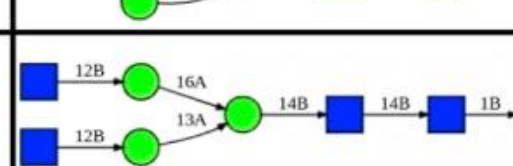	bDGlcNAc(1→2)aDMan(1→6)<br>[bDGlcNAc(1→2)aDMan(1→3)]bDMan(1→4)bDGlcNAc(1→4)bDGlcNAc(1→)PROB-1081
G37	N1117	FA3	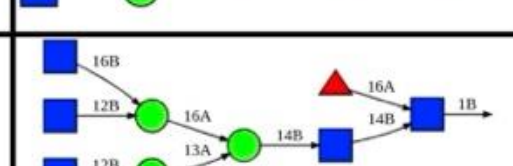	bDGlcNAc(1→6)[bDGlcNAc(1→2)]aDMan(1→6)<br>[bDGlcNAc(1→2)aDMan(1→3)]bDMan(1→4)bDGlcNAc(1→4)[aLFuc(1→6)]bDGlcNAc(1→)PROB-1117
G38	S311	O-glycan	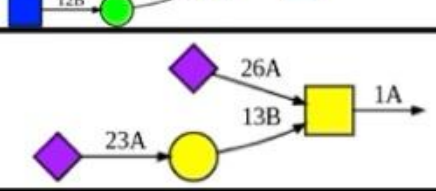	aDNeu5Ac(2→3)bDGal(1→3)[aDNeu5Ac(2→6)]aDGalNAc(1→)PROB-311

CHAIN C

#	SITE	TYPE	STRUCTURE	SEQUENCE
G39	N66	M5	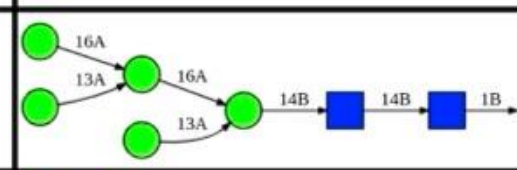	aDMan(1→6)[aDMan(1→3)]aDMan(1→6) [aDMan(1→3)]bDMan(1→4)bDGlcNAc(1→4)bDGlcNAc(1→)PROC-66
G40	N110	M8	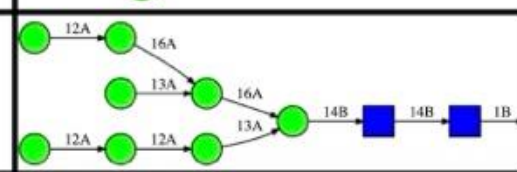	aDMan(1→2)aDMan(1→6)[aDMan(1→3)]aDMan(1→6) [aDMan(1→2)aDMan(1→2)aDMan(1→3)]bDMan(1→4)bDGlcNAc(1→4)bDGlcNAc(1→)PROC-110
G41	N120	M5	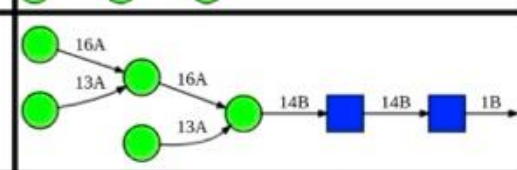	aDMan(1→6)[aDMan(1→3)]aDMan(1→6) [aDMan(1→3)]bDMan(1→4)bDGlcNAc(1→4)bDGlcNAc(1→)PROC-120
G42	N145	FA2	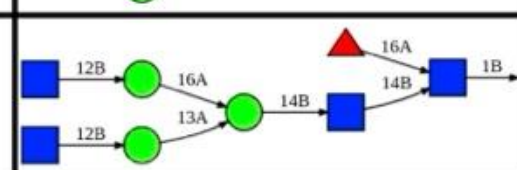	bDGlcNAc(1→2)aDMan(1→6)[bDGlcNAc(1→2)aDMan(1→3)]bDMan(1→4)bDGlcNAc(1→4) [aLFuc(1→6)]bDGlcNAc(1→)PROC-145
G43	N159	FA2G2S1	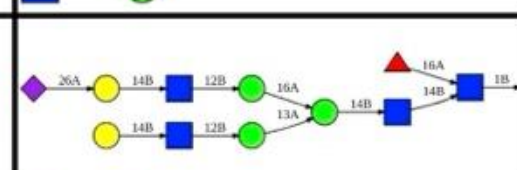	aDNeu5Ac(2→6)bDGal(1→4)bDGlcNAc(1→2)aDMan(1→6) [bDGal(1→4)bDGlcNAc(1→2)aDMan(1→3)]bDMan(1→4)bDGlcNAc(1→4) [aLFuc(1→6)]bDGlcNAc(1→)PROC-159
G44	N228	M9	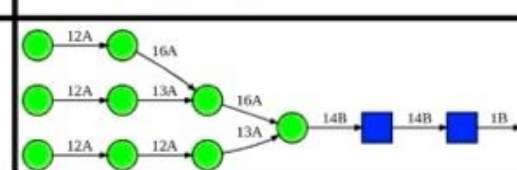	aDMan(1→2)aDMan(1→6)[aDMan(1→2)aDMan(1→3)]aDMan(1→6) [aDMan(1→2)aDMan(1→2)aDMan(1→3)]bDMan(1→4)bDGlcNAc(1→4)bDGlcNAc(1→)PROC-228
G45	N270	A2	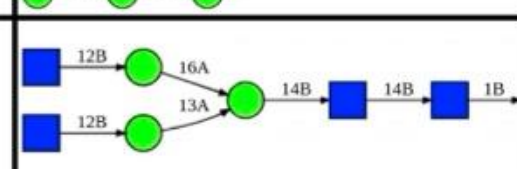	bDGlcNAc(1→2)aDMan(1→6) [bDGlcNAc(1→2)aDMan(1→3)]bDMan(1→4)bDGlcNAc(1→4)bDGlcNAc(1→)PROC-270
G46	N319	FA3G3S1	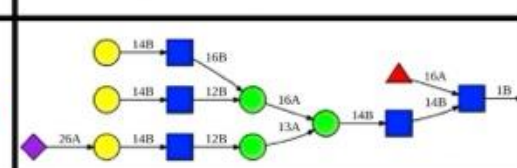	aDNeu5Ac(2→6)bDGal(1→4)bDGlcNAc(1→2)aDMan(1→3)[bDGal(1→4)bDGlcNAc(1→6) [bDGal(1→4)bDGlcNAc(1→2)]aDMan(1→6)]bDMan(1→4)bDGlcNAc(1→4) [aLFuc(1→6)]bDGlcNAc(1→)PROC-319
G47	N331	FA2	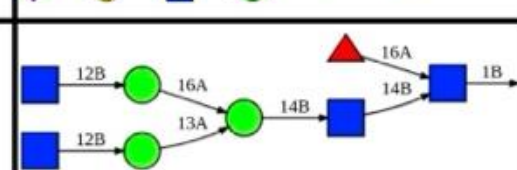	bDGlcNAc(1→2)aDMan(1→6)[bDGlcNAc(1→2)aDMan(1→3)]bDMan(1→4)bDGlcNAc(1→4) [aLFuc(1→6)]bDGlcNAc(1→)PROC-331
G48	N358	FA3G3S1	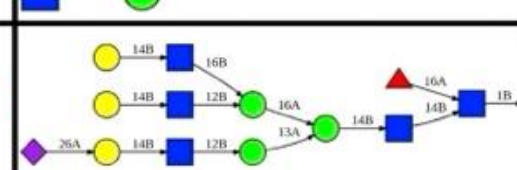	aDNeu5Ac(2→6)bDGal(1→4)bDGlcNAc(1→2)aDMan(1→3)[bDGal(1→4)bDGlcNAc(1→6) [bDGal(1→4)bDGlcNAc(1→2)]aDMan(1→6)]bDMan(1→4)bDGlcNAc(1→4) [aLFuc(1→6)]bDGlcNAc(1→)PROC-358
G49	N590	M5	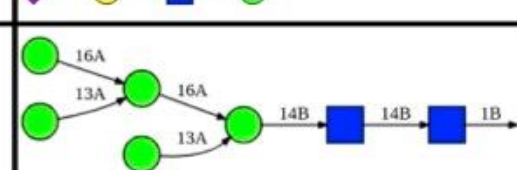	aDMan(1→6)[aDMan(1→3)]aDMan(1→6) [aDMan(1→3)]bDMan(1→4)bDGlcNAc(1→4)bDGlcNAc(1→)PROC-590
G50	N603	FA2	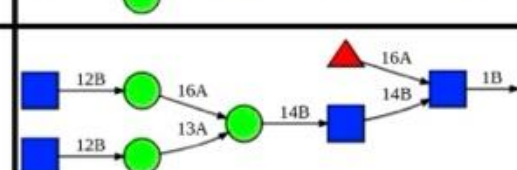	bDGlcNAc(1→2)aDMan(1→6)[bDGlcNAc(1→2)aDMan(1→3)]bDMan(1→4)bDGlcNAc(1→4) [aLFuc(1→6)]bDGlcNAc(1→)PROC-603
G51	N692	M5	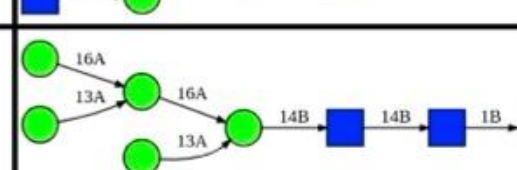	aDMan(1→6)[aDMan(1→3)]aDMan(1→6) [aDMan(1→3)]bDMan(1→4)bDGlcNAc(1→4)bDGlcNAc(1→)PROC-692
G52	N700	M6	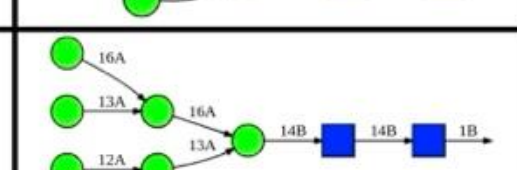	aDMan(1→6)[aDMan(1→3)]aDMan(1→6) [aDMan(1→2)aDMan(1→3)]bDMan(1→4)bDGlcNAc(1→4)bDGlcNAc(1→)PROC-700
G53	N784	M5	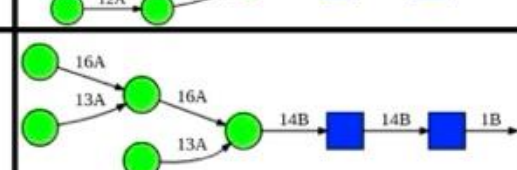	aDMan(1→6)[aDMan(1→3)]aDMan(1→6) [aDMan(1→3)]bDMan(1→4)bDGlcNAc(1→4)bDGlcNAc(1→)PROC-784
G54	N1057	M5	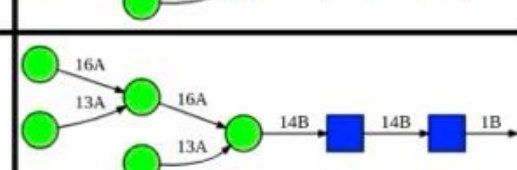	aDMan(1→6)[aDMan(1→3)]aDMan(1→6) [aDMan(1→3)]bDMan(1→4)bDGlcNAc(1→4)bDGlcNAc(1→)PROC-1057
G55	N1081	Hybrid G1S1	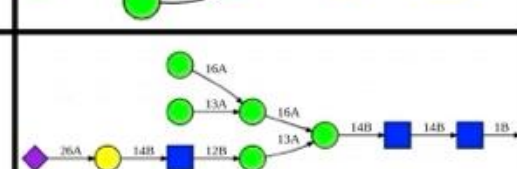	aDNeu5Ac(2→6)bDGal(1→4)bDGlcNAc(1→2)aDMan(1→3)[aDMan(1→6) [aDMan(1→3)]aDMan(1→6)]bDMan(1→4)bDGlcNAc(1→4)bDGlcNAc(1→)PROC-1081
G56	N1117	FA2	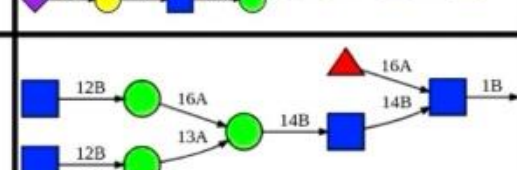	bDGlcNAc(1→2)aDMan(1→6)[bDGlcNAc(1→2)aDMan(1→3)]bDMan(1→4)bDGlcNAc(1→4) [aLFuc(1→6)]bDGlcNAc(1→)PROC-1117
G57	S311	O-glycan	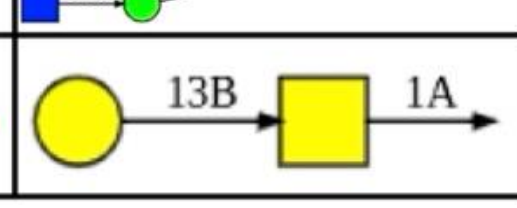	bDGal(1→3)aDGalNAc(1→)PROC-323

Table S4: Summary of average trimeric domain areas (Å²) for NTD, RBM, SD2, and FPPR in SHC014 WT and mutans. Values represent mean ± standard deviation across three independent 1-μs simulation replicates.

Variant	Domains			
	NTD (A^2)	RBM (A^2)	SD2 (A^2)	FPPR (A^2)
WT	2772.9 ± 36.0	661.3 ± 46.0	1640.1 ± 21.9	1233.1 ± 42.9
F294L	2766.8 ± 49.5	667.3 ± 7.5	1649.0 ± 15.0	1262.9 ± 38.7
A835D	2746.0 ± 40.1	674.7 ± 15.4	1626.5 ± 38.1	1380.8 ± 57.9
DM	2716.4 ± 29.3	663.2 ± 6.5	1670.4 ± 37.7	1351.8 ± 23.4

Table S5: Statistical analysis of FPPR trimeric area differences across SHC014 spike variants. One-way ANOVA results (F = 8.2078, p = 0.0080) indicate overall differences among variants. Pairwise comparisons were performed using Welch's t-test, and corresponding p-values are reported.

FPPR One Way ANOVA: F = 8.2078; p-value = 0.0080	
Variant	FPPR Domain (T-test p-value)
WT vs F294L	0.4225
WT vs A835D	0.0273
WT vs DM	0.0231
F294L vs A835D	0.0506
F294L vs DM	0.0366
A835D vs DM	0.4878
